# Overcoming naphthoquinone deactivation: rhodium-catalyzed C-5 selective C–H iodination as a gateway to functionalized derivatives†
†Electronic supplementary information (ESI) available: Experimental procedures and characterisation data for all compounds are provided. CCDC 1441683–1441694. For ESI and crystallographic data in CIF or other electronic format see DOI: 10.1039/c6sc00302h


**DOI:** 10.1039/c6sc00302h

**Published:** 2016-03-02

**Authors:** Guilherme A. M. Jardim, Eufrânio N. da Silva Júnior, John F. Bower

**Affiliations:** a School of Chemistry , University of Bristol , Bristol , BS8 1TS , UK . Email: john.bower@bris.ac.uk; b Institute of Exact Sciences , Department of Chemistry , Federal University of Minas Gerais , Belo Horizonte , MG 31270-901 , Brazil . Email: eufranio@ufmg.br

## Abstract

We report a Rh-catalyzed method for the C-5 selective C–H iodination of naphthoquinones and show that complementary C-2 selective processes can be achieved under related conditions. C–C bond forming derivatizations of the C-5 iodinated products provide a gateway to previously inaccessible A-ring analogues. The present study encompasses the first catalytic directed *ortho*-functionalizations of simple (non-bias) naphthoquinones. The strategic considerations outlined here are likely to be applicable to C–H functionalizations of other weakly coordinating and/or redox sensitive substrates.

## Abstract 2

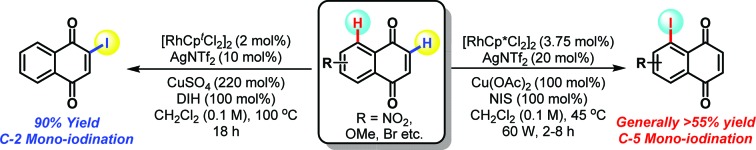
Rh-catalyzed C-5 selective C–H iodination of naphthoquinones provides a gateway to previously inaccessible A-ring analogues. C-2 selective processes can be achieved under related conditions.

## Introduction

**Scheme 1:**
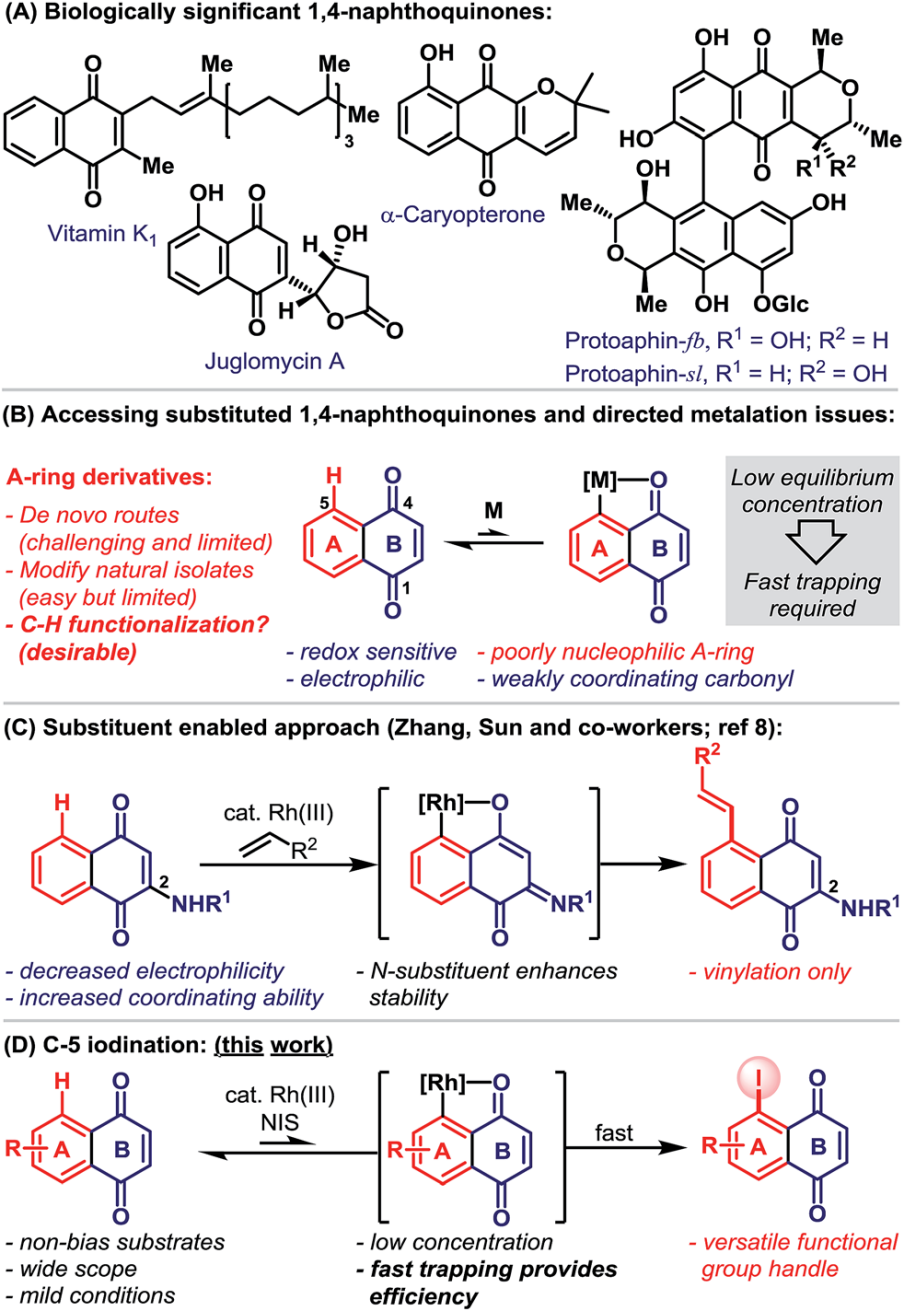


1,4-Naphthoquinones function as redox cyclers and alkylating agents in a wide range of biological processes.^1^ For example, vitamin K encompasses a family of 2-methyl-1,4-naphthoquinones that act as cofactors for the post-translational carboxylation of glutamic acid residues, a process that is essential to blood coagulation and bone metabolism.1*e* Other notable naphthoquinones include the juglomycins,2*a* dimeric pyranonaphthoquinones, such as protoaphin-fb and protoaphin-sl2b–e and the lapachones (Scheme 1A).2*f* Because of their biological importance, significant efforts are devoted to the synthesis and evaluation of a wide range of naphthoquinone derivatives.^3^ While methods for the modification of the quinone B-ring are reasonably well established,^4^ flexible protocols that allow the direct functionalization of the benzenoid A-ring are rare (Scheme 1B).^5^ This situation is exacerbated by the limitations associated with *de novo* naphthoquinone construction.^6^ Consequently, medicinal studies on A-ring analogues are, in the main, limited to simple derivatives of natural isolates.

Catalytic directed *ortho*-C–H metalation has emerged as a powerful platform for the preparation of diverse aromatic compounds.^7^ However, its application to the modification of naphthoquinones is limited by (a) their susceptibility to reduction, (b) their high electrophilicity, (c) the low nucleophilicity of the benzenoid A-ring and (d) the weak coordinating ability of the B-ring carbonyls (Scheme 1B). Indeed, we are aware of only one protocol that enables catalytic carbonyl directed C–H functionalization of the naphthoquinone A-ring (Scheme 1C).^8^ Here, Rh(iii)-catalyzed C-5 vinylation required the strategic installation of a C-2 amino substituent, which was crucial for facilitating cyclometalation.

Carbonyl directed *ortho*-metalation with late transition metals is usually a reversible process,^7^,^9^ where the equilibrium depends upon both the nucleophilicity of the arene and the coordinating strength of the directing group (Scheme 1B). Neither aspect is favorable for naphthoquinones, so an efficient process must necessarily rely upon fast trapping of the metalated intermediate. In considering this, we sought to introduce a synthetically versatile handle under conditions that avoid nucleophilic or reducing reagents. Consequently, we were drawn to the *ortho*-C–H iodination protocol reported by Glorius and co-workers,^9^ which involves the trapping of cyclometalated aryl-Rh(iii) complexes with NIS. This reagent is highly reactive, oxidative and also non-nucleophilic, such that it seemed well suited to C-5 selective iodination. In this report, we outline the development and scope of this protocol, which provides, for the first time, a C–H activation-based gateway to diverse A-ring analogues (Scheme 1D). Additionally, we disclose that, in certain cases, fine tuning of the Rh-system enables a complete switch to C-2 selective iodination.

## Results and discussion

Preliminary studies involved exposing naphthoquinone **1a** to a variety of electrophilic iodine sources in the presence of *in situ* generated cationic Rh(iii)-systems (Table 1). These experiments revealed that achieving both high conversion and high C-5 regioselectivity was likely to be challenging. [RhCp*Cl_2_]_2_/AgNTf_2_ in combination with NIS and Cu(OAc)_2_ resulted in only a 39% yield of **2a**, albeit with 10 : 1 selectivity over C-2 regioisomer **3a** (entry 1). Other electrophilic iodine sources were less effective; for example, DIH led to substantial quantities of C-2 adduct **3a** and bis-iodinated product **4** (entry 2). Extensive optimization efforts were undertaken to identify an effective system, and, in part, these studies focussed on the use of other acetate ligated Lewis acids in combination with a range of Rh(iii)-precatalysts (entries 3–8).^10^ However none of these systems were especially effective, with the key issue being competitive degradation of the iodinating agent under the reaction conditions. To resolve this, microwave conditions were investigated.^11^ Pleasingly, by heating at 65 W (50 °C), a 54% yield of **2a** was obtained after just 2.5 hours (entry 9). Under these conditions, the major byproduct was bis-iodinated adduct **4**, which was formed in 14% yield. Further refinement led to the conditions outlined in entry 10, which deliver **2a** in 69% yield and with high selectivity over **3a** and **4**.^12^ The conversion of **1a** to **2a** has been achieved previously in 34% overall yield, but this required a substrate specific 5 step sequence *via* a potentially hazardous diazonium intermediate.^13^ Thus, the efficiency and generality (*vide infra*) of the new protocol described here is both notable and synthetically enabling.

**Table 1:** Selected optimization results

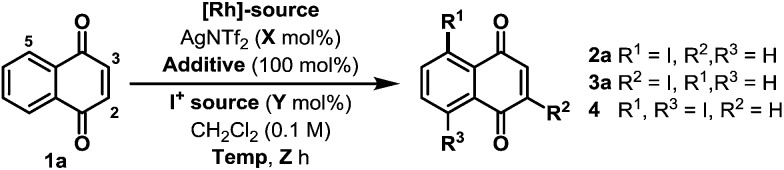								
Entry	Rh-source	*X*	Additive	I^+^ source	*Y*	Temp/°C	*Z*	**2a** : **3a** : **4**
1	[RhCp*Cl_2_]_2_ (2.5%)	10	Cu(OAc)_2_	NIS	220	120	22	39 : 8 : 0
2	[RhCp*Cl_2_]_2_ (2.5%)	10	Cu(OAc)_2_	DIH	140	120	22	4 : 14 : 10
3	[RhCp*Cl_2_]_2_ (2.5%)	10	Cu(OPiv)_2_	NIS	140	120	22	14 : 0 : 0
4	[RhCp*Cl_2_]_2_ (2.5%)	10	Zn(OAc)_2_	NIS	140	120	22	18 : 0 : 0
5	[RhCp*Cl_2_]_2_ (4%)	10	Cu(OAc)_2_	NIS	120	100	16	44 : 5 : 0
6	[RhCp*^i-pr^Cl_2_]_2_ (4%)	10	Cu(OAc)_2_	NIS	120	100	16	39 : 8 : 0
7	[RhCp*^CF_3_^Cl_2_]_2_ (4%)	10	Cu(OAc)_2_	NIS	120	100	16	3 : 0 : 0
8	[RhCp*(OAc)_2_] (4%)	10	Cu(OAc)_2_	NIS	120	100	16	32 : 2 : 0
9	[RhCp*Cl_2_]_2_ (4%)	10	Cu(OAc)_2_	NIS	120 50^*a*^	(65 W)	2.5	54 : 0 : 14
10	[RhCp*Cl_2_]_2_ (3.75%)	20	Cu(OAc)_2_	NIS	100 45^*a*^	(60 W)	2	69 : 0 : 5

^*a*^External temperature of the reaction vessel. NIS = *N*-iodosuccinimide. DIH = 1,3-diiodo-5,5-dimethylhydantoin. Cp*^i-Pr^ = isopropyl tetramethylcyclopentadienyl. Cp*^CF_3_^ = trifluoromethyl tetramethylcyclopentadienyl.

The scope of the new process is outlined in Table 2. Naphthoquinones **1b–e** possessing C-8 substitution underwent efficient iodination to provide targets **2b–e** in good to excellent yield. As expected, the efficiency of the process decreases as the benzenoid ring becomes more electron deficient, and, accordingly, nitro adduct **2f** was generated in only 24% yield. Systems with substituents at C-6 or C-7 can potentially deliver two different regioisomeric products. Perhaps as a result of secondary coordination by the methoxy group, naphthoquinone **1g** afforded selectively adduct **2g**, wherein iodination has occurred at the more hindered *ortho*-position. Conversely, methyl-substituted system **1h** favored iodination at the less hindered *ortho*-site to deliver iodide **2h** in 63% yield. Halogen substituents are tolerated and bromo- and chloro-naphthoquinones **1j–l** were converted to targets **2j–l** in synthetically useful yields. For **2j**, the high *ortho*-regioselectivity may reflect the higher basicity of the C-4 carbonyl of **1j**. In all cases, only trace quantities (≤5%) of bis-iodinated and C-2/3 iodinated products were observed (*cf.***4** and **3a**), and no significant iodination occurred in the absence of Rh-catalyst. Structural assignments were based on detailed NMR analysis (DEPT, COSY, HSQC, HMBC) and X-ray structures of **2a–c**, **2f**, **2j** and **2k**.^14^

**Table 2:** Scope of the C-5 selective iodination protocol^*a*^

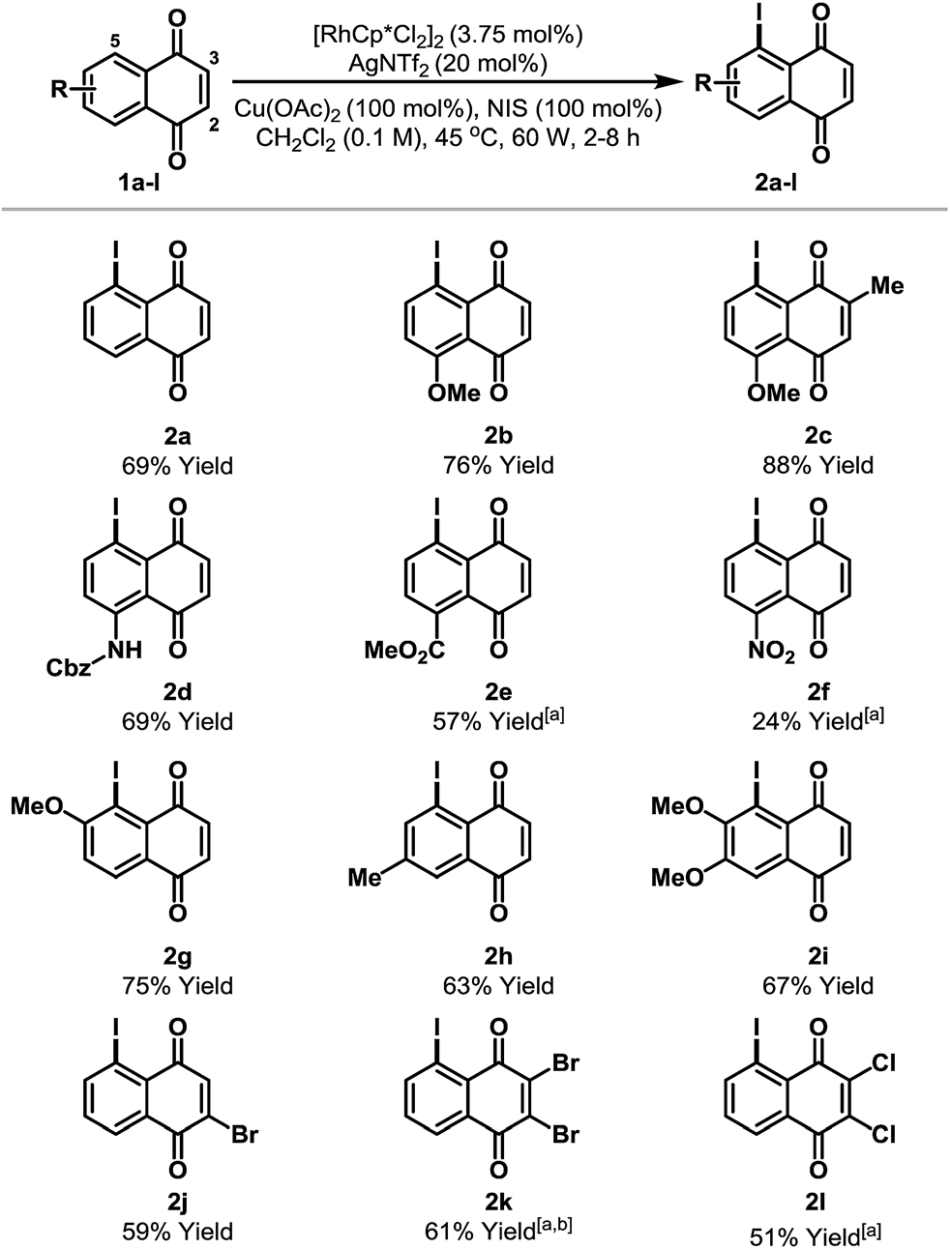		

^*a*^<5% bis-iodination was observed in all cases. NIS = *N*-iodosuccinimide. [a] 5 mol% [RhCp*Cl_2_]_2_ and 27 mol% AgNTf_2_ were used. [b] Run at 65 °C and 75 W.

**Scheme 2:**
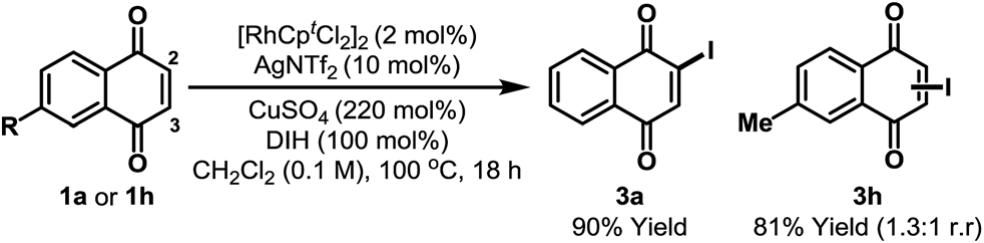
C-2 selective iodination. DIH = 1,3-diiodo-5,5-dimethylhydantoin. Cp^*t*^ = 1,3-di-*tert*-butylcyclopentadienyl.

During optimization we noted that the regioselectivity of iodination is strongly influenced by the nature of the Lewis acidic additive. Acetate based systems consistently provided high selectivity for C-5, likely *via* a Rh-acetate promoted concerted metalation–deprotonation mechanism (*cf.*Table 1, entry 8).^15^ By switching from Cu(OAc)_2_ to CuSO_4_ we were able to develop a complementary C-2 selective iodination protocol (Scheme 2).^10^,^16^ Under optimized conditions, iodination of **1a** generated **3a** in 90% yield and with complete regioselectivity. Perez and co-workers have shown that morpholine–iodine complex can convert **1a** to **3a**, but in only 35% yield and as a mixture of products.^17^ For unsymmetrical systems, such as **1h**, C-2 *vs.* C-3 selectivity was not readily controlled and **3h** was formed as a mixture of these two regioisomers.^18^ At the present stage, C-3 selective iodination of systems possessing substitution at C-2 is not feasible, perhaps due to steric inhibition of the C–H metalation event.

**Fig. 1:**
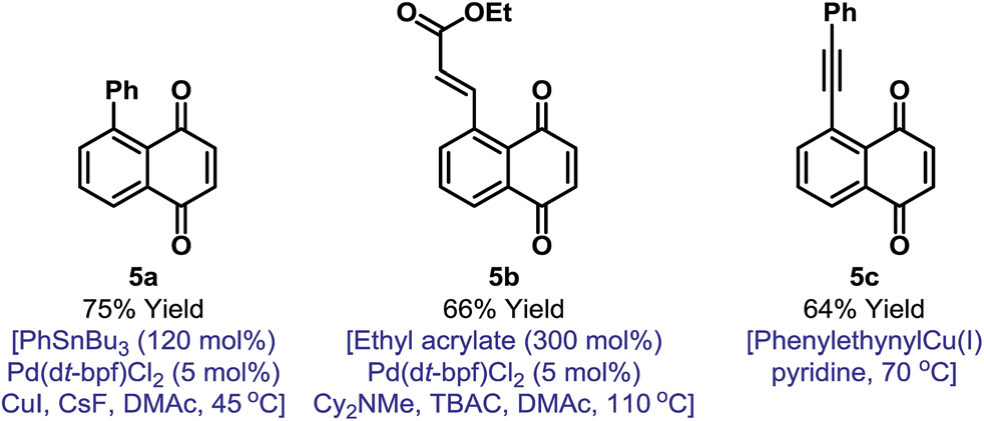
C–C bond forming derivatizations of **2a**. TBAC = tetra-*n*-butylammonium chloride, DMAc = *N*,*N*-dimethylacetamide.

In principle, the activation mode employed here should enable other selective naphthoquinone C–H functionalizations. However, as outlined in the introduction, efficient processes likely require highly reactive and non-reducing coupling partners. Accordingly, we have been unable to achieve direct C–H activation based C–C bond formations.^19^ However, the iodinated products described here provide a gateway to this important goal (Fig. 1). Because of the synthetic inaccessibility of A-ring halogenated naphthoquinones, Pd-catalyzed cross-couplings involving the benzenoid ring have not been developed. This aspect is challenging because the quinone moiety can act as an oxidant or ligand for Pd.^20^ For example, arylation of **2a** could not be achieved under Suzuki conditions and only decomposition products were observed. After extensive investigation, we established that mild Stille cross-couplings^21^ are effective and, using this approach, arylated derivative **5a** was isolated in high yield. Heck reactions are another promising avenue and Pd(0)-catalyzed reaction of **2a** with ethyl acrylate delivered **5b** in 66% yield.^22^ To date, alkynylation under Sonogashira conditions has not been fruitful but the use of stoichiometric alkynyl copper(i) reagents is feasible and this allowed the isolation of **5c** in 64% yield.^13^ The studies outlined in Fig. 1 validate short and diversifiable entries to previously challenging naphthoquinone targets.

**Scheme 3:**
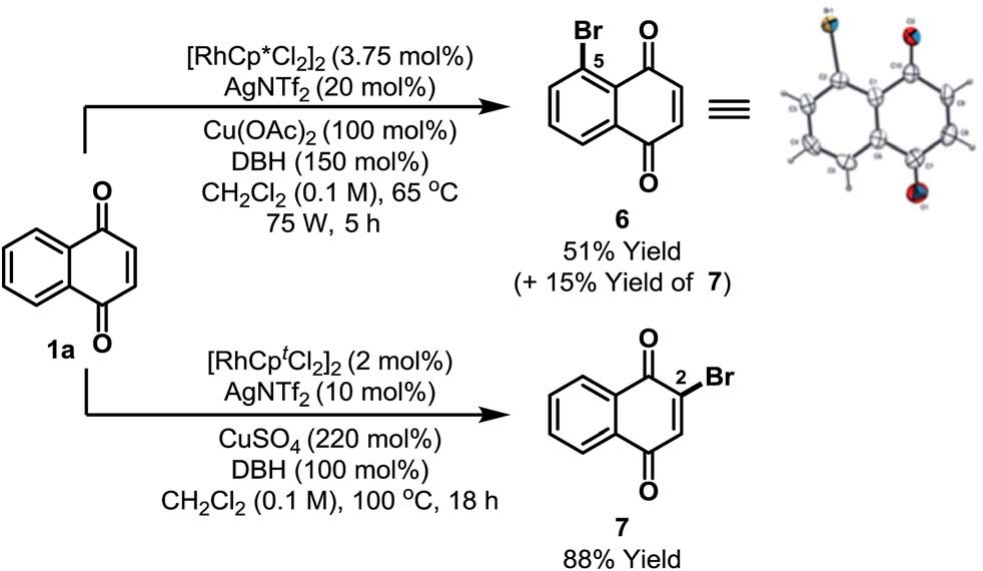
C-5 and C-2 selective bromination. DBH = 1,3-dibromo-5,5-dimethylhydantoin. Cp^*t*^ = 1,3-di-*tert*-butylcyclopentadienyl.

Preliminary results suggest that other highly electrophilic reagents might also be effective for C-5 selective functionalization (Scheme 3). Using DBH, Rh-catalyzed C-5 selective bromination of **1a** proceeded in 66% yield to afford a 7 : 2 mixture of C-5 and C-2 bromides **6**/**7**; the structure of **6** was confirmed by single crystal X-ray diffraction.^13^ The most direct previous entry to **6** involved oxidation of 2-bromonaphthalene, but this afforded the target in only 15% yield and as a complex mixture of isomers.^23^ Alternative C-2 selective bromination can be achieved by adapting the conditions outlined in Scheme 2 and this enabled the selective formation of **7** in 88% yield from **1a**.

## Conclusions

In conclusion, we report an efficient and reliable methodology for C-5 selective C–H iodination of naphthoquinoidal compounds and show that complementary C-2 selective processes can be achieved under related conditions. To the best of our knowledge, the present study provides the first method for catalytic directed *ortho*-functionalization of simple (non-bias) naphthoquinones. The iodinated products are amenable to C–C bond forming derivatizations and this enables flexible modifications to the naphthoquinone A-ring. The chemistry opens up new avenues for biological investigation and is likely to be of wide general interest. In broader terms, the strategic considerations outlined here may guide the development of catalytic C–H functionalizations involving other weakly coordinating and/or redox sensitive substrates.

## Supplementary Material

Supplementary information

Crystal structure data
