# Supplementary material for: Overcoming naphthoquinone deactivation: rhodium-catalyzed C-5 selective C–H iodination as a gateway to functionalized derivatives
Source: Chem Sci. 2016 Mar 2;7(6):3780–4. doi: 10.1039/c6sc00302h (PMC6013821; doi:10.1039/c6sc00302h)
Supplement: Supplementary file 1 [file SC-007-C6SC00302H-s001.pdf]

Electronic Supplementary Material (ESI) for Chemical Science.
This journal is © The Royal Society of Chemistry 2016

Overcoming Naphthoquinone Deactivation: Rhodium-Catalyzed C-5 Selective C-H Iodination as a Gateway to Functionalized Derivatives

Guilherme A. M. Jardim,[a,b] Eufrânio N. da Silva Júnior[a]* and John F. Bower[b]*

[a]Institute of Exact Sciences, Department of Chemistry, Federal University of Minas Gerais, CEP 31270-901, Belo Horizonte, MG, Brazil

[b]School of Chemistry, University of Bristol, Bristol, BS8 1TS, United Kingdom

Corresponding authors: eufranio@ufmg.br and john.bower@bris.ac.uk

Contents

A) General experimental details 2

B) Synthesis of substrates and known compounds 3

C) General microwave procedure for the iodination reactions 15

D) General thermal procedure for halogenation at the C-2 position 27

E) Procedures for derivatization reactions 30

F) Copies of NMR spectra of novel compounds 33

General Experimental Details

Starting materials sourced from commercial suppliers were used as received unless otherwise stated. All reagents requiring purification were purified using standard laboratory techniques according to methods published by Perrin, Armarego, and Perrin (Pergamon Press, 1966). Catalytic reactions were run under an atmosphere of dry nitrogen or argon; glassware, syringes and needles were either flame dried immediately prior to use or placed in an oven (200 °C) for at least 2 h and allowed to cool either in a desiccator or under an atmosphere of nitrogen or argon; liquid reagents, solutions or solvents were added via syringe through rubber septa; solid reagents were added inside a glovebox. All optimization reactions were filtered through a sinter funnel charged with a pad of celite and silica for copper removal. Coupling partners for the Heck and Stille reactions were distilled before use. Anhydrous solvents were obtained by distillation using standard procedures or by passage through drying columns supplied by Anhydrous Engineering Ltd. Anhydrous dichloromethane (CH_2Cl_2) was purged with argon for 10 minutes prior use. Flash column chromatography (FCC) was performed using silica gel (Aldrich 40-63 µm, 230-400 mesh). Thin layer chromatography (TLC) was performed using aluminium backed 60 F254 silica plates. Visualization was achieved by UV fluorescence or a basic $KMnO_4$ solution and heat. Proton nuclear magnetic resonance spectra (NMR) were recorded using either a Varian 400 MHz or Varian 500 MHz. ^{13}C NMR spectra were recorded at 100 MHz or 125 MHz as stated. Chemical shifts (δ) are given in parts per million (ppm). Peaks are described as singlets (s), doublets (d), double doublets (dd), triplets (t) double triplets (dt), quartets (q), heptets (hept) and multiplets (m). ^{1}H and ^{13}C NMR spectra were referenced to the appropriate residual solvent peak. Coupling constants (*J*) are quoted to the nearest 0.5 Hz. All assignments of NMR spectra were based on 2D NMR data ($DEPT^{135}$, COSY, HSQC and HMBC). In situ yields were determined by employing 1,4-dinitrobenzene as an internal standard. Mass spectra were recorded using a Brüker Daltonics FT-ICRMS Apex 4e 7.0T FT-MS (ESI^+ mode) and Shimadzu GCMS QP2010+ (EI^+ mode). Infrared spectra were recorded on a Perkin Elmer Spectrum One FTIR spectrometer as thin films or solids compressed on a diamond plate. Melting points were determined using Stuart SMP30 melting point apparatus and are uncorrected.

Synthesis of Substrates and Known Compounds:

1,4-Naphthoquinone (**1a**) was purchased from Alfa Aesar and purified via reduced pressure sublimation using a cold finger sublimation apparatus (50 °C, 0.9 mbar) and stored in a glovebox to prevent contact with moisture. All commercially available naphthoquinones and further commercial chemicals were purchased from Sigma Aldrich, Alfa Aesar, Strem Chemicals and Santa Cruz Biotechnology. $[RhCp^{*}Cl_2]_2$ and $[RhCp^{t}Cl_2]_2$ were purchased from Sigma Aldrich.

General procedure for the synthesis of substituted Cp ligands:

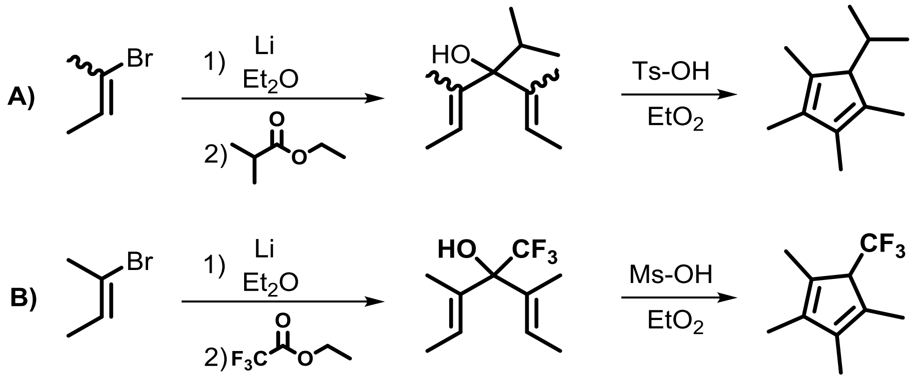

A 100 mL 2-necked round-bottomed flask equipped with a magnetic stir bar and a reflux condenser was charged with lithium wire (44.8 mmol, 310 mg, cut into 1 cm lengths) and dry diethyl ether (1.5 mL). An initial portion of 2-bromo-2-butene (10.0 mmol, 1.0 mL, mixture of isomers used in reaction A, pure *E*-isomer used in reaction B) was added to the stirred solution via syringe dropwise over the course of several minutes. At this point, the reaction initiated, as indicated by the evolution of heat and bubbling. Additional dry diethyl ether (15 mL) was added, and additional 2-bromo-2-butene (13.0 mmol, 1.3 mL) was added slowly to keep the reaction at reflux. After the addition was complete, stirring was continued for an additional 1h. The reaction mixture was then cooled to 0 °C in an ice bath and ethyl isopropylate (reaction A, 11.2 mmol, 1.5 mL) or ethyl trifluoroacetate (reaction B, 11.2 mmol, 1.3 mL) diluted in dry diethyl ether (2 mL) was added dropwise. The reaction mixture was poured into saturated aqueous NH_4Cl (30 mL) and extracted with diethyl ether (5 × 20 mL). The combined organic layers were dried over Na_2SO_4, and the solvent was removed under reduced

pressure to obtain the Nazarov adducts as yellow oils. *The products were used without further purification in the next step.* Under an inert atmosphere the crude Nazarov adducts were added quickly via syringe to a solution of p-toluenesulfonic acid (reaction A, 10.0 mmol, 1.90 g) or methanesulfonic acid (reaction B, 92.5 mmol, 6 mL) in diethyl ether (15 mL). The mixture was stirred for 1h after which it was quenched with saturated aqueous Na_2CO_3 (30 mL). The organic layer was separated and the aqueous phase was extracted with diethyl ether (3 × 50 mL). The combined organic layers were dried over Na_2SO_4, and the solvent was removed by rotary evaporation. The crude products were purified via reduced pressure distillation. **5-isopropyl-1,2,3,4-tetramethylcyclopenta-1,3-diene:** 1.16 g, 63% yield, distilled at 78 °C, 10 mBar as a colorless oil; **HRMS(EI$^+$):** 164.1566 $[M]^+$. Cald. for $[C_{12}H_{20}]$: 164.1565; *^{1}H NMR analysis showed a mixture of 3 isomers.* **1,2,3,4-tetramethyl-5-(trifluoromethyl)cyclopenta-1,3-diene:** 1.15 g, 54% yield, distilled at 86 °C, 10 mBar as a light yellow oil; **HRMS(EI$^+$):** 190.0965 $[M]^+$. Cald. for $[C_{10}H_{13}F_3]$: 190.0969; *^{1}H NMR analysis showed a mixture of 2 major isomers.* Data are consistent with those reported in the literature.[1,2,3]

General procedure for the synthesis of $[RhCp^{*i\text{-}Pr}Cl_2]_2$ and $[RhCp^{*CF3}Cl_2]_2$:

A 100 ml reaction tube was charged with $RhCl_3.XH_2O$ (1.00 mmol, 209 mg), the requisite diene (1.50 mmol) and methanol (50 mL). The tube was sealed with a screw cap and the mixture was heated at 75 °C for 48h. The mixture was cooled to room temperature and methanol was removed under reduced pressure to yield the crude product. This was washed sequentially with pentane (50 mL) and dry diethyl ether (3 × 50 mL). The resulting crystalline powder was dried under high vacuum overnight. Recrystallization from petroleum ether/chloroform afforded analytically pure crystals for characterization by single crystal X-ray diffraction.

$[RhCp^{*i\text{-}Pr}Cl_2]_2$

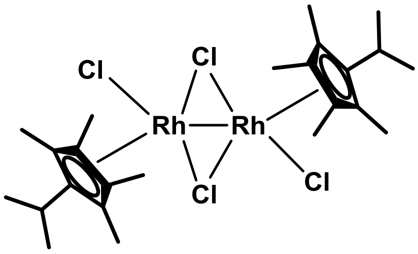

The product was obtained by the general procedure described above. Reaction with 5-isopropyl-1,2,3,4-tetramethylcyclopenta-1,3-diene (1.50 mmol, 246 mg) afforded [RhCp$^{*i\text{-Pr}}$Cl$_2$]$_2$ (484 mg, 72% yield) as deep red crystals; **m.p. (°C)** = 250.0 (degradation) (Petrol/CHCl$_3$); **HRMS (EI$^+$):** 671.9831 [M]$^+$. Cald. for [$C_{24}H_{38}Cl_4Rh_2$]: 671.9838; **^{1}H NMR (500 MHz, CDCl$_3$) *δ*:** 2.57 (hept, *J* = 7.1 Hz, 2H), 1.69 (s, 12H), 1.57 (s, 12H), 1.26 (d, *J* = 7.1 Hz, 6H); **^{13}C NMR (125 MHz, CDCl$_3$) *δ*:** 97.5 (d, $J^{\text{Rh-C}}$ = 8.9 Hz), 95.2 (d, $J^{\text{Rh-C}}$ = 10.0 Hz), 94.0 (d, $J^{\text{Rh-C}}$ = 9.1 Hz), 24.9, 20.6, 10.4, 9.5. *The structure of the product was confirmed by X-ray diffraction, as shown below.*

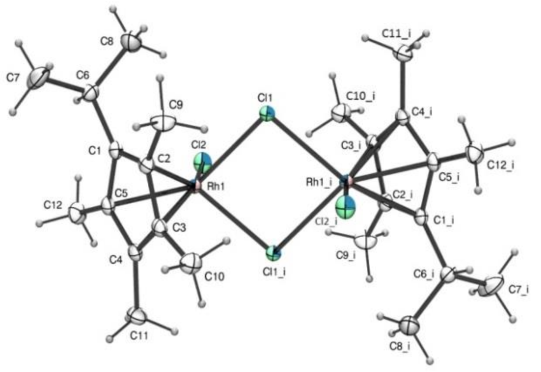

Figure 1: Crystal structure of [RhCp$^{*i\text{-Pr}}$Cl$_2$]$_2$.

[RhCp^{*CF3}Cl$_2$]$_2$

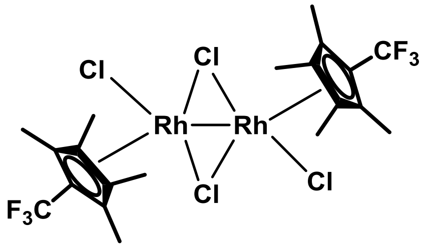

The product was obtained by the general procedure described above. Reaction with 1,2,3,4-tetramethyl-5-(trifluoromethyl)cyclopenta-1,3-diene (1.50 mmol, 285 mg) afforded [RhCp^{*CF3}Cl$_2$]$_2$ (499 mg, 69% yield) as purple crystals; **m.p. (°C)** = 250.0 (degradation) (Petrol/CHCl$_3$); **HRMS (EI$^+$):** 723.8639 [M]$^+$. Cald. for [$C_{20}H_{24}Cl_4F_6Rh_2$]: 723.8646; **^{1}H NMR (500 MHz, CDCl$_3$) *δ*:** 1.93 (q, $J^{\text{F-H}}$ = 0.9 Hz, 12H), 1.74 (s, 12H); **^{13}C NMR (125 MHz, CDCl$_3$) *δ*:** 124.2 (q, $J^{\text{F-C}}$ = 274.1 Hz, CF$_3$),

101.4 (d, $J^{Rh\text{-}C}$ = 8.1 Hz), 97.3 (d, $J^{Rh\text{-}C}$ = 8.1 Hz), 10.1 (q, $J^{F\text{-}C}$ = 2.2 Hz), 9.5; **^{19}F NMR (400 MHz, $CDCl_3$) δ:** -54.8 (s, 3F). *The structure of the product was confirmed by X-ray diffraction, as shown below.* Data are consistent with those reported in the literature.[4]

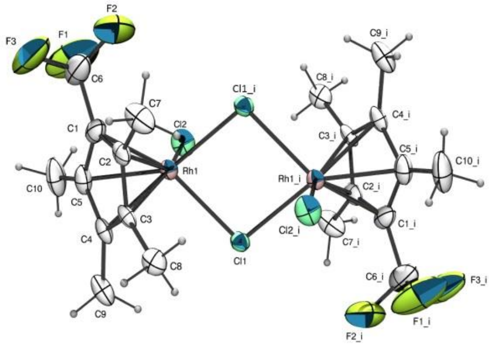

Figure 2: Crystal structure of $[RhCp^{*CF3}Cl_2]_2$.

$[RhCp^*(OAc)_2(H_2O)]$

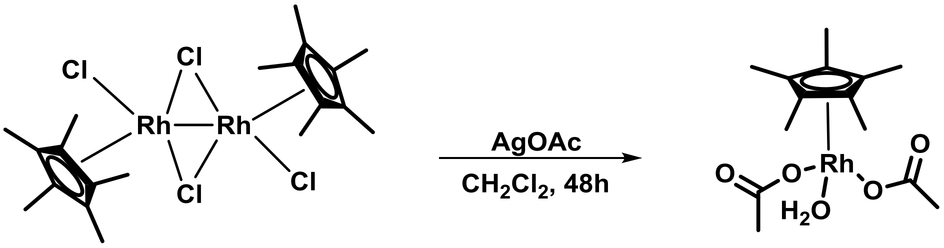

A 50 mL 2-necked round-bottomed flask equipped with a magnetic stir bar was charged with $[RhCp^*Cl_2]_2$ (0.77 mmol, 473 mg) and silver acetate (3.74 mmol, 625 mg). The flask was evacuated, flushed with N_2 and CH_2Cl_2 (30 mL) was added via syringe. The reaction mixture was stirred at room temperature for 48h. The mixture was then filtered and the solvent was removed under reduced pressure. The resulting gummy solid was washed with pentane (50 mL) and dried under vacuum overnight to afford $[RhCp^*(OAc)_2(H_2O)]$ (239 mg, 64% yield) as a deep orange gummy solid. Crystallization from petroleum ether/chloroform afforded analytically pure crystals for characterization; **m.p. (°C)** = 250.0 (degradation) (Petrol/$CHCl_3$); **HRMS (EI$^+$):** 374.0589 $[M]^+$. Cald. for $[C_{14}H_{23}O_5Rh]$: 374.0601; **^{1}H NMR (500 MHz, $CDCl_3$) δ:** 1.91 (s, 6H), 1.65 (s, 15H) ; **^{13}C NMR (125 MHz, $CDCl_3$) δ:** 181.2, 98.4 (d, $J^{Rh\text{-}C}$ = 7.4

Hz), 90.9 (d, J^{Rh-C} = 9.7 Hz), 24.1, 8.8. *The structure of the product was confirmed by X-ray diffraction, as shown below.* Data are consistent with those reported in the literature.[5]

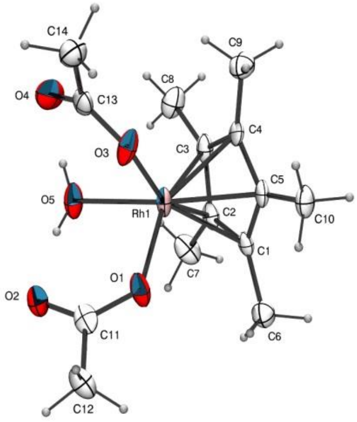

Figure 3: Crystal structure of [RhCp*(OAc)$_2$(H$_2$O)].

<u>General procedure for the synthesis of substrates 1b and 1c:</u>

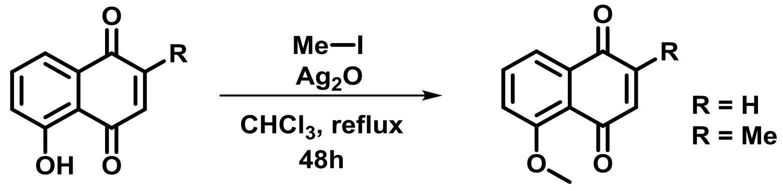

To a solution of the corresponding naphthoquinone (1.00 mmol) in $CHCl_3$ (30 mL) was added Ag_2O (2.00 mmol, 463 mg) and iodomethane (2.00 mmol, 125 µL). The reaction was then heated at reflux for 48h. After cooling to room temperature, the mixture was filtered through a pad of celite and the solvent was removed under reduced pressure. The crude product was purified by FCC, under the conditions noted.

5-Methoxy-1,4-naphthoquinone (1b)

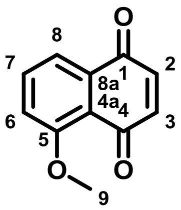

5-Hydroxy-1,4-naphthoquinone (1.00 mmol, 174 mg) was used. Purification by FCC (hexane/ethyl acetate 5:1) afforded **1b** (137 mg, 73% yield) as yellow crystals; **m.p. (°C)** = 181.7-181.9 (Petrol/CH_2Cl_2); **^{1}H NMR (400 MHz, $CDCl_3$) δ:** 7.77-7.60 (m, 2H), 7.29 (d, J = 8.0 Hz, 1H), 6.84 (s, 2H), 3.98 (s, 3H); **^{13}C NMR (100 MHz, $CDCl_3$) δ:** 185.2, 184.3, 159.6, 140.9, 136.2, 135.0, 134.0, 119.7, 119.1, 117.9, 56.5. Data are consistent with those reported in the literature.[6]

5-Methoxy-2-methyl-1,4-naphthoquinone (1c)

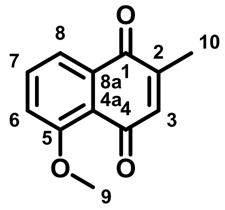

5-Hydroxy-2-methyl-1,4-naphthoquinone (1.00 mmol, 188 mg) was used. Purification by FCC (hexane/ethyl acetate 5:1) afforded **1c** (158 mg, 78% yield) as yellow crystals; **m.p. (°C)** = 94.8-95.1 (Petrol/CH_2Cl_2); **^{1}H NMR (400 MHz, $CDCl_3$) δ:** 7.75 (d, J = 7.7 Hz, 1H), 7.65 (t, J = 8.4 Hz, 1H), 7.28 (d, J = 8.4 Hz, 1H), 6.73 (s, 1H), 4.00 (s, 3H), 2.13 (s, 3H); **^{13}C NMR (100 MHz, $CDCl_3$) δ:** 185.7, 184.4, 159.4, 145.4, 137.9, 134.6, 134.4, 120.0, 119.4, 117.7, 56.5, 15.8. Data are consistent with those reported in the literature.[7]

Benzyl (5,8-dioxo-5,8-dihydronaphthalen-1-yl)carbamate (1d)

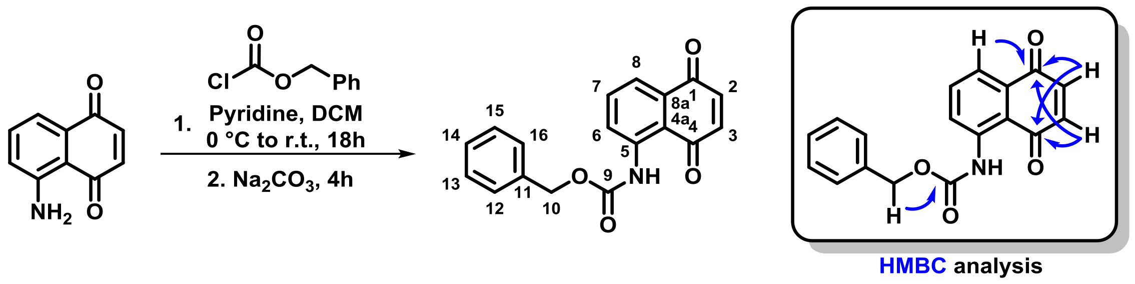

To a solution of 5-amino-1,4-naphthoquinone (1.00 mmol, 173 mg) in CH_2Cl_2 (10 ml) was added pyridine (1.30 mmol, 105 µL). The reaction was cooled to 0 °C and a solution of benzyl chloroformate (1.00 mmol, 143 µL) in CH_2Cl_2 (5 mL) was added over a 30 minutes period. The resulting mixture was warmed to room temperature and

stirred for a further 18h. Saturated aqueous Na_2CO_3 (10 mL) was added and the mixture was stirred for another 4h. The organic layer was separated, dried with anhydrous Na_2SO_4 and concentrated under reduced pressure. Purification of the residue by FCC (hexane/EtOAc 10:1) afforded **1d** (197 mg, 64% yield) as an orange powder; **m.p (°C)** = 117.2-117.9 (Petrol/CH_2Cl_2); **IR (solid, cm^{-1}) *ν*:** 3253 (m) 2927 (w), 1730 (s), 1579 (s), 1256 (s), 743 (m); **HRMS (ESI$^+$):** 330.0740 $[M+Na]^+$. Cald. for $[C_{18}H_{13}NNaO_4^+]$: 330.0737; **^{1}H NMR (400 MHz, $CDCl_3$) *δ*:** 11.44 (s, N-H), 8.84 (d, *J* = 8.5 Hz, C**6**-H), 7.78 (d, *J* = 6.3 Hz, C**8**-H), 7.71 (t, *J* = 8.5 Hz, C**7**-H), 7.51-7.30 (m, C(**12-16**)-H), 6.93 (d, *J* = 10.3 Hz, C**2**-H), 6.90 (d, *J* = 10.3 Hz, C**3**-H), 5.25 (s, C**10**-H_2); **^{13}C NMR (100 MHz, $CDCl_3$) *δ*:** 188.6 (C**4**), 184.5 (C**1**), 153.6 (C**9**), 141.6 (C**5**), 140.0 (C**3**), 137.8 (C**2**), 135.8 (C**11**), 135.5 (C**7**), 132.3 (C**8a**), 128.6 (C**12**, C**16**), 128.4 (C**14**), 128.3 (C**13**, C**15**) , 124.6 (C**6**), 121.2 (C**8**), 115.8 (C**4a**), 67.3 (C**10**). *The structural assignment of the product was supported by HMBC analysis, as indicated above.*

Penta-2,4-dienoic acid

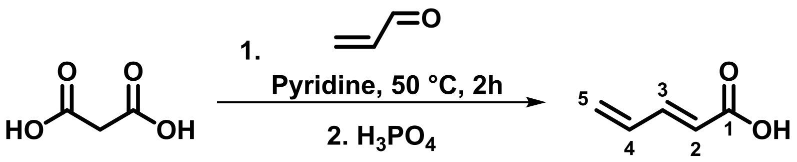

Malonic acid (288 mmol, 30.0 g) was added portion wise to pyridine (50 mL). Once all the malonic acid had dissolved, acrylaldehyde (21 mL, 315 mmol) was added dropwise over 3 minutes. The mixture was the heated at 55 °C for 2 h. After cooling to 4 °C, the pH was adjusted to 3 with H_3PO_4 (85% in H_2O). The mixture was extracted with diethyl ether (3 x 50 mL), dried with Na_2SO_4 and the solvent was removed under reduced pressure. The crude product was recrystallized from petroleum ether to afford penta-2,4-dienoic acid (15.3 g, 54% yield) as a pale yellow powder; **m.p. (°C)** = 66.5-68.3 (Petrol/CH_2Cl_2); **^{1}H NMR (400 MHz, $CDCl_3$) *δ*:** 11.8 (s, 1H), 7.36 (q, *J* = 15.6 Hz, 1H), 6.56-6.39 (m, 1H), 5.92 (d, *J* = 15.6 Hz, 1H), 5.67 (dd, *J* = 16.9, 1.0 Hz, 1H), 5.56 (d, *J* = 9.8, 1H); **^{13}C NMR (100 MHz, $CDCl_3$) *δ*:** 172.6, 147.1, 134.5, 126.8, 121.3. Data are consistent with those reported in the literature.[8]

5,8-Dioxo-1,4,4a,5,8,8a-hexahydronaphthalene-1-carboxylic acid

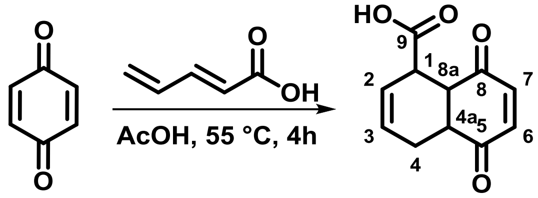

Penta-2,4-dienoic acid (102 mmol, 10.0 g) and benzoquinone (95.0 mmol, 10.3 g) were dissolved in acetic acid (100 mL). The solution was then heated at 55 °C for 4h. After cooling to room temperature, the solvent was removed under reduced pressure and the crude product was suspended in ethanol (100 mL) and filtered. The precipitate was washed with water (50 mL), ethanol (100 mL) and diethyl ether (50 mL) and dried under reduced pressure to afford 5,8-dioxo-1,4,4a,5,8,8a-hexahydronaphthalene-1-carboxylic acid (8.80 g, 45%) as a silver powder; **m.p. (°C)** = 153.5-155.0 (Petrol/CH_2Cl_2); **^{1}H NMR (400 MHz, DMSO-*d6*) *δ*:** 12.3 (s, 1H), 6.76 (d, *J* = 10.2 Hz, 1H), 6.65 (d, *J* = 10.2 Hz, 1H), 6.02 (d, *J* = 9.6 Hz, 1H), 5.62-5.59 (m, 1H), 4.04 (t, *J* = 5.1 Hz, 1H), 3.27-3.16 (m, 1H), 2.23-2.15 (m, 1H), 2.08-1.86 (m, 1H); **^{13}C NMR (100 MHz, DMSO-*d6*) *δ*:** 200.7, 198.3, 173.8, 172.8, 140.2, 138.7, 125.0, 124.5, 48.3, 47.2, 25.9. Data are consistent with those reported in the literature.[9]

Methyl 5,8-dimethoxy-1,4-dihydronaphthalene-1-carboxylate

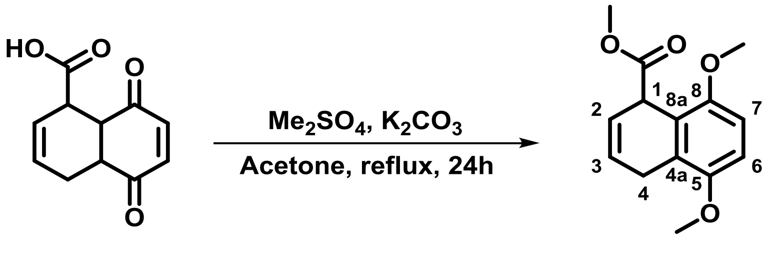

5,8-Dioxo-1,4,4a,5,8,8a-hexahydronaphthalene-1-carboxylic acid (14.6 mmol, 3.01 g) and K_2CO_3 (79.7 mmol, 11.0 g) were suspended in acetone (40 mL). To this solution was added dimethyl sulphate (4 mL) dropwise over 5 minutes. The mixture was then heated to reflux for 24h. After cooling to room temperature, the precipitate was filtered, washing with acetone, and the filtrate was evaporated under reduced pressure to afford methyl 5,8-dimethoxy-1,4-dihydronaphthalene-1-carboxylate (3.22 g, 89% yield) as a colourless powder. The product was used without further purification; **m.p. (°C) =** 99.5-101,3 (Acetone); **^{1}H NMR (400 MHz, $CDCl_3$) *δ*:** 6.73 (dd, *J* = 8.8, 2.0 Hz, 1H), 6.69

(dd, J = 8.8, 2.0 Hz, 1H), 6.08 (d, J = 10.1 Hz, 1H), 5.91 (d, J = 7.8 Hz, 1H), 4.54-4.46 (m, 1H), 3.80 (s, 3H), 3.76 (s, 3H), 3.69 (s, 3H), 3.44-3.20 (m, 2H); **^{13}C NMR (100 MHz, $CDCl_3$) δ:** 173.4, 151.0, 150.8, 126.8, 124.5, 122.2, 121.8, 108.3, 107.5, 55.8, 55.6, 52.1, 42.3, 24.5. Data are consistent with those reported in the literature.[12]

Methyl 5,8-dimethoxy-1-naphthoate

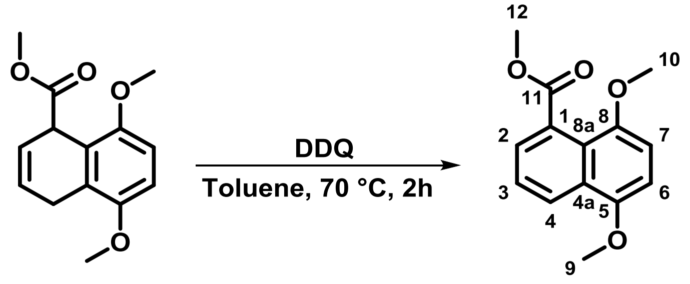

Methyl 5,8-dimethoxy-1,4-dihydronaphthalene-1-carboxylate (11.5 mmol, 2.86 g) and DDQ (13.2 mmol, 2.99 g) were dissolved in toluene (30 mL). The mixture was then heated at 70 °C for 2h. The mixture was cooled to room temperature and solvent was removed under reduced pressure. The residue was dissolved in CH_2Cl_2 (50 mL), washed sequentially with aqueous NaOH (100 mL, 0.5 M) and saturated aqueous Na_2SO_3 (100 mL), and dried with Na_2SO_4. The organic portion was concentrated under reduced pressure and the crude product was purified by FCC (hexane/ethyl acetate 10:1) to afford methyl 5,8-dimethoxy-1-naphthoate (2.04 g, 72% yield) as an off-white solid; **m.p. (°C) =** 104.5-104.9 (CH_2Cl_2); **^{1}H NMR (400 MHz, $CDCl_3$) δ:** 8.31 (dd, J = 7.7, 2.1 Hz, 1H), 7.53-7.42 (m, 2H), 6.80 (d, J = 8.4 Hz, 1H), 6.75 (d, J = 8.4 Hz, 1H), 3.97 (s, 3H), 3.96 (s, 3H), 3.90 (s, 3H); **^{13}C NMR (100 MHz, $CDCl_3$) δ:** 172.0, 149.8, 148.6, 129.3, 126.6, 125.2, 124.8, 123.7, 122.4, 106.0, 104.1, 56.8, 55.8, 52.3. Data are consistent with those reported in the literature.[10]

Methyl 5,8-dioxo-5,8-dihydronaphthalene-1-carboxylate (1e)

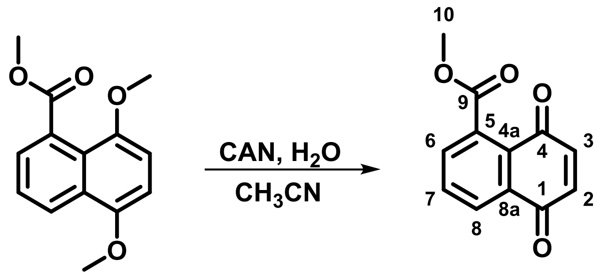

Methyl 5,8-dimethoxy-1-naphthoate (4.00 mmol, 985 mg) was dissolved in acetonitrile (15 mL) and the solution was cooled to 4 °C. A solution of cerium ammonium nitrate (12.4 mmol, 6.80 g) in water (15 mL) was then added dropwise over 1 minute. The mixture was stirred at room temperature for 30 minutes prior to extraction with ethyl acetate (50 mL). The organic layer was dried with Na_2SO_4 and evaporated under reduced pressure. Purification of the residue by FCC (hexane/ethyl acetate 19:1) afforded **1e** (761 mg, 88% yield) as yellow crystals; **m.p. (°C)** = 92.3-93.0 (Petrol/CH_2Cl_2); **^{1}H NMR (400 MHz, $CDCl_3$) *δ*:** 8.17 (dd, *J* = 7.8, 1.3 Hz, 1H), 7.79 (t, *J* = 7.7 Hz, 1H), 7.68 (dd, *J* = 7.8, 1.3 Hz, 1H), 6.98 (d, *J* = 10.5 Hz, 1H), 6.97 (d, *J* = 10.5 Hz, 1H), 3.98 (s, 3H); **^{13}C NMR (100 MHz, $CDCl_3$) *δ*:** 172.0, 149.8, 148.6, 129.3, 126.6, 125.2, 124.8, 123.7, 122.4, 106.0, 104.1, 56.8, 55.8, 52.3. Data are consistent with those reported in the literature.[11]

5-Nitro-1,4-naphthoquinone and 6-Nitro-1,4-naphthoquinone (1f)

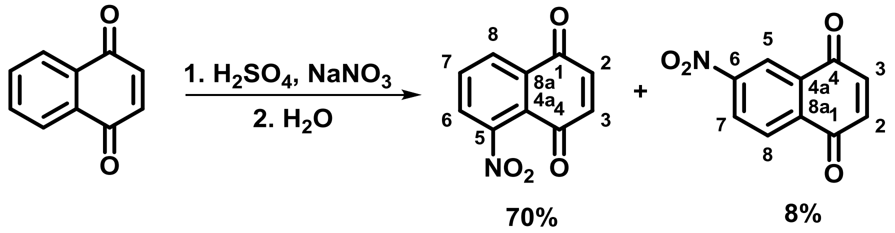

1,4-Naphthoquinone (25.3 mmol, 4.00 g) was added portionwise, over 10 minutes, to vigorously stirred concentrated sulfuric acid (50 ml) at 0 °C. A solution of $NaNO_3$ (164 mmol, 14.0 g) in concentrated sulfuric acid (16 mL) was then added over 3 minutes. The iced bath was removed and the mixture was stirred at room temperature for 1h. The reaction mixture was then heated at 40 °C for 20 minutes. The mixture was cooled to room temperature, and the resulting orange solution was poured onto ice (250 g). This gave a yellow precipitate, which was filtered, washed with water and dried under vacuum. The crude product was dissolved in CH_2Cl_2 (50 mL) and filtered through a pad of silica gel. The filtrate was concentrated *in vacuo* and the residue was crystallized from hexane/ CH_2Cl_2 (5:1). The crystals were washed with pentane (50 mL) to afford the product (4.00 g, 78% yield, 8:1 *ortho:meta*) as a mixture of isomers (determined by ^{1}H NMR analysis). These isomers were separated by FCC (petrol/ethyl acetate 10:1). **5-nitro-1,4-naphthoquinone:** 3.49 g, 68% yield as yellow needles; **m.p. (°C)** = 166.5-167.1 (Petrol/CH_2Cl_2); **^{1}H NMR (400 MHz, $CDCl_3$) *δ*:** 8.29 (dd, *J* = 7.8, 1.3 Hz, 1H),

7.91 (t, J = 7.9 Hz, 1H), 7.74 (dd, J = 7.9, 1.3 Hz, 1H), 7.06 (d, J = 10.4 Hz, 1H), 7.02 (d, J = 10.4 Hz, 1H); **^{13}C NMR (100 MHz, CDCl$_3$) δ:** 182.6, 181.3, 148.3, 139.1, 138.1, 134.7, 132.7, 128.9, 127.5, 122.9. **6-nitro-1,4-naphthoquinone:** 360 mg, 7% yield as light yellow needles; **m.p. (°C)** = 147.1-148.2 (Petrol/CH_2Cl_2); **^{1}H NMR (400 MHz, CDCl$_3$) δ:** 8.89 (d, J = 2.3 Hz, 1H), 8.57 (dd, J = 8.2, 2.4 Hz, 1H), 8.30 (d, J = 8.1 Hz, 1H), 7.15 (d, J = 10.5 Hz, 1H), 7.12 (d, J = 10.6 Hz, 1H); **^{13}C NMR (100 MHz, CDCl$_3$) δ:** 183.2, 182.7, 151.1, 139.2, 138.9, 135.2, 133.0, 128.4, 128.0, 121.8. Data for both compounds are consistent with those reported in the literature.[12]

5-Amino-1,4-naphthoquinone

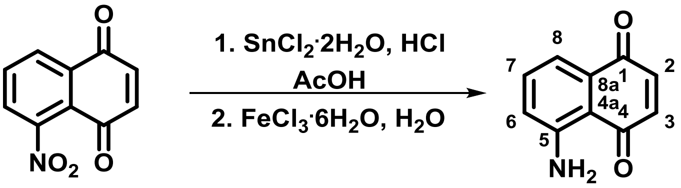

A solution of 5-nitro-1,4-naphthoquinone (5.00 mmol, 1.02 g) in AcOH (35 mL) was heated to 50 °C in a 250 mL round bottom flask. Then, a solution of $SnC1_2 \cdot 2H_2O$ (26.6 mmol, 6.00 g) in concentrated HCl (10 mL) was added to the mixture. The mixture was heated at 70 °C for 40 minutes and cooled to room temperature. A solution of $FeCl_3 \cdot 6H_2O$ (32.2 mmol, 8.70 g) in cold H_2O (10 mL) was added to the mixture and stirring was continued for 30 minutes. The mixture was poured onto ice (100 g) and at 4 °C overnight. The resulting precipitate was filtered, washed with water and dried under reduced pressure to afford 5-amino-1,4-naphthoquinone (528 mg, 61% yield) as purple crystals; **m.p. (°C)** = 189.1-190.4 (Petrol/CH_2Cl_2); **^{1}H NMR (400 MHz, CDCl$_3$) δ:** 7.47-7.39 (m, 2H), 6.98-6.92 (m, 1H), 6.88 (d, J = 10.3 Hz, 1H), 6.84 (d, J = 10.3 Hz, 1H), 6.67 (s, 2H); **NMR (100 MHz, CDCl$_3$) δ:** 187.1, 185.4, 150.1, 140.6, 137.3, 134.6, 132.9, 123.2, 117.0, 112.3. Data are consistent with those reported in the literature.[10]

General procedure for the synthesis of substrates 1g and 1i:

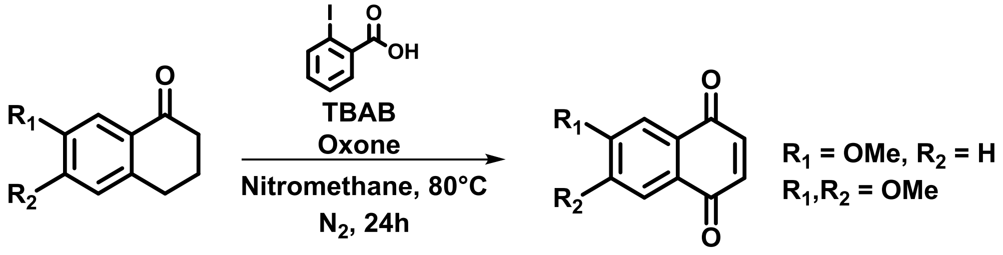

To an oven dried re-sealable reaction tube were added the corresponding tetralone (1.00 mmol), 2-iodobenzoic acid (0.20 mmol, 59.2 mg), tetrabutylammonium bromide (0.05 mmol, 15.3 mg) and Oxone (5.00 mmol, 3.10 g). The flask was placed under a N_2 atmosphere and nitromethane (10 mL) was added via syringe. The reaction was heated at 80 °C for 24h. The mixture was then cooled to room temperature, filtered through a pad of celite and the solvent was removed under reduced pressure. The crude product was purified by FCC, under the conditions noted.

6-Methoxy-1,4-naphthoquinone (1g)

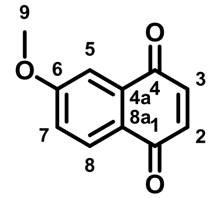

7-Methoxy-3,4-dihydronaphthalen-1(2*H*)-one (1.00 mmol, 176 mg) was used. Purification by FCC (hexane/ethyl acetate 5:1) afforded **1g** (128 mg, 68% yield) as a yellow powder; **m.p. (°C)** = 112.8-113.1 (Petrol/CH_2Cl_2); **^{1}H NMR (400 MHz, $CDCl_3$) *δ*:** 8.02 (d, *J* = 8.8 Hz, 1H), 7.50 (d, *J* = 1.7 Hz, 1H), 7.21 (dd, *J* = 8.7, 2.4 Hz, 1H), 6.94 (d, *J* = 10.4 Hz, 1H), 6.93 (d, *J* = 10.4 Hz, 1H) 3.95 (s, 3H); **^{13}C NMR (100 MHz, $CDCl_3$) *δ*:** 185.2, 184.1, 164.1, 139.0, 138.2, 133.9, 128.9, 125.5, 120.5, 109.6, 55.9. Data are consistent with those reported in the literature.[13]

6,7-Dimethoxy-1,4-naphthoquinone (1i)

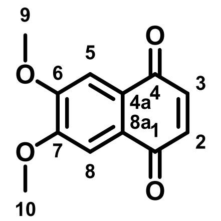

6,7-Dimethoxy-3,4-dihydronaphthalen-1(2*H*)-one (1.00 mmol, 206 mg) was used. Purification by FCC (hexane/ethyl acetate 5:1) afforded **1i** (137 mg, 63% yield) as an orange powder; **m.p. (°C)** = 231.3-231.9 (Petrol/CH_2Cl_2); **^{1}H NMR (400 MHz, $CDCl_3$) *δ*:** 7.48 (s, 2H), 6.87 (s, 2H), 4.01 (s, 6H); **^{13}C NMR (100 MHz, $CDCl_3$) *δ*:** 184.5, 153.5, 138.3, 126.7, 107.8, 56.5. Data are consistent with those reported in the literature.[9]

General microwave procedure for the iodination reactions:

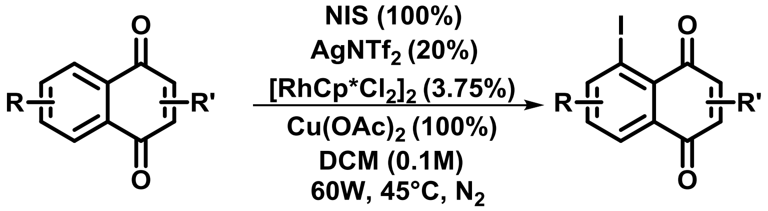

In a glovebox, an oven dried re-sealable reaction tube was charged with the corresponding naphthoquinone (0.10 mmol), $[RhCp^*Cl_2]_2$ (3.75 mol %, 2.3 mg), silver bis(trifluoromethanesulfonyl)imide (20 mol %, 7.8 mg), *N*-iodosuccinimide (100 mol %, 0.10 mmol, 22.5 mg) and anhydrous copper acetate (100 mol %, 0.10 mmol, 18.2 mg). The tube was removed from the glovebox and an inert atmosphere was maintained. Anhydrous CH_2Cl_2 (1.0 mL) was added via syringe and the tube was sealed. The mixture was irradiated in a CEM Discover microwave apparatus in open flask mode (60 W) and the nitrogen flow was adjusted to maintain a reaction temperature of 45 °C. After cooling, the reaction mixture was filtered through a pad of celite and concentrated under reduced pressure. The residue was purified by FCC, under the conditions noted.

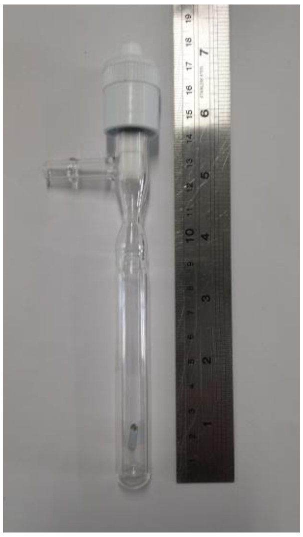 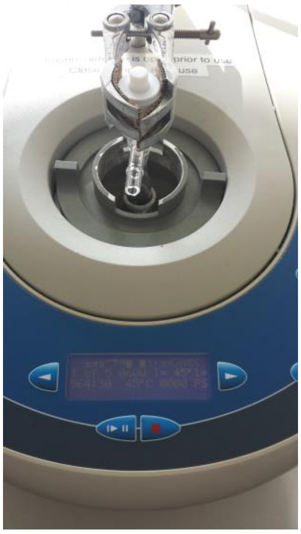

LHS: customized re-sealable reaction tube. **RHS:** Typical reaction set-up

5-Iodo-1,4-naphthoquinone (2a)

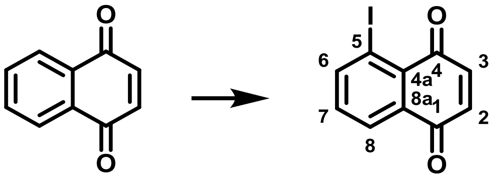

The product was obtained by the general microwave procedure described above. The reaction was conducted for 2h and purification by FCC (toluene) afforded iodinated product **2a** (19.6 mg, 69% yield) as red crystals; **m.p. (°C)** = 167.8-168.6 (Petrol/CH_2Cl_2); **IR (solid, cm^{-1}) *ν*:** 3071 (w), 1665 (s), 1318 (m), 782 (m); **HRMS (EI$^+$):** 283.9335 $[M]^+$. Cald. for $[C_{10}H_5IO_2]$: 283.9334; **^{1}H NMR (400 MHz, $CDCl_3$) *δ*:** 8.36 (d, J = 7.8 Hz, C**6**-H), 8.14 (d, J = 7.8 Hz, C**8**-H), 7.35 (t, J = 7.8 Hz, C7-H), 7.02 (d, J = 10.3 Hz, C**3**-H), 6.94 (d, J = 10.3 Hz, C**2**-H); **^{13}C NMR (100 MHz, $CDCl_3$) *δ*:** 183.6 (C**1)**, 183.2 (C**4**), 148.2 (C**6**), 139.7 (C**2**), 137.1 (C**3**), 134.3 (C**8a**), 133.7 (C**7**), 130.7 (C**4a**), 127.6 (C**8**), 92.7 (C**5**-I). *The structure of product **2a** was confirmed by X-ray diffraction, as shown below.*

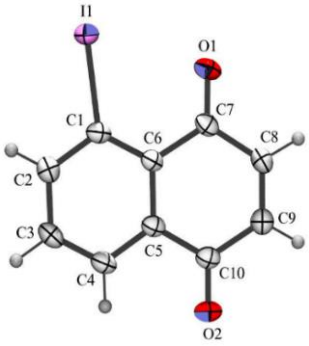

Figure 4: Crystal structure of compound **2a**.

5,8-Diiodo-1,4-naphthoquinone (4)

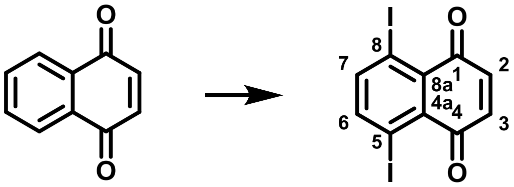

In addition to **2a**, bis-iodinated by-product **4** (2.0 mg, 5% yield) was obtained as deep red crystals; **m.p. (°C)**: 161.8-162.3 (Petrol/CH_2Cl_2); **IR (solid, cm^{-1}) *ν*:** 2922 (w), 1660 (s), 1051 (m), 820 (m); **HRMS (EI$^+$):** 409.8290 $[M]^+$. Cald. for $[C_{10}H_4I_2O_2]$: 409.8301; **^{1}H NMR (400 MHz, $CDCl_3$) *δ*:** 7.94 (s, C**6**-H, C**7**-H), 6.99 (s, C**2**-H, C**3**-H); **^{13}C NMR (101 MHz, $CDCl_3$) *δ*:** 182.3 (C**1**, C**4**), 147.7 (C**6**, C**7**), 138.0 (C**2**, C**3**), 133.0 (C**4a**, C**8a**), 93.8 (C**5**-I, C**8**-I). *The structure of by-product **4** was confirmed by X-ray diffraction, as shown below.*

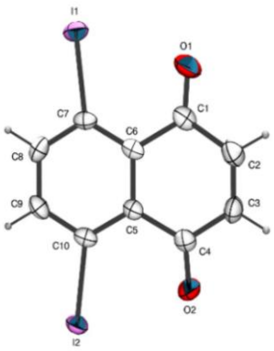

Figure 5: Crystal structure of compound **4**.

5-Iodo-8-methoxy-1,4-naphthoquinone (2b)

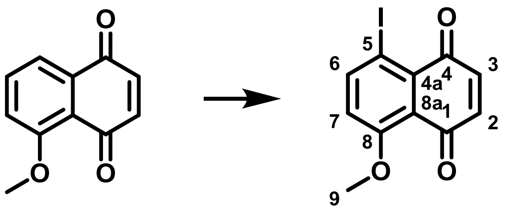

The product was obtained by the general microwave procedure described above. The reaction was conducted for 2h and purification by FCC (toluene) afforded iodinated product **2b** (23.9 mg, 76% yield) as red crystals; **m.p. (°C) =** 184.6-185.4 (Petrol/CH_2Cl_2); **IR (solid, cm^{-1}) *ν*:** 2924 (w), 1656 (s), 1285 (m), 1026 (m); **HRMS (ESI$^+$):** 314.9518 $[M+H]^+$. Cald. for $[C_{11}H_8IO_3]^+$: 314.9518; **^{1}H NMR (400 MHz, $CDCl_3$) *δ*:** 8.27 (d, *J* = 9.0 Hz, C**6**-H), 7.00 (d, *J* = 9.0 Hz, C**7**-H), 6.91 (d, *J* = 10.2 Hz, C**3**-H), 6.82 (d, *J* = 10.2 Hz, C**2**-H), 3.99 (s, C**9**-H_3); **^{13}C NMR (100 MHz, $CDCl_3$) *δ*:** 183.6 (C**4**), 183.3 (C**1**), 160.4 (C**8**), 148.8 (C**6**), 139.2 (C**2**), 136.9 (C**3**), 132.4 (C**4a**), 122.4 (C**8a**), 118.8 (C**7**), 81.6 (C**5**-I), 56.7 (C**9**). *The structure of product **2b** was confirmed by X-ray diffraction, as shown below.*

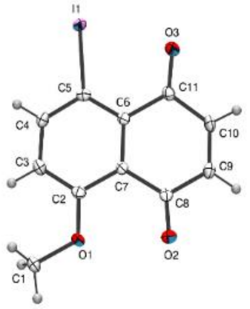

Figure 6: Crystal structure of compound **2b**.

8-Iodo-5-methoxy-2-methyl-1,4-naphthoquinone (2c)

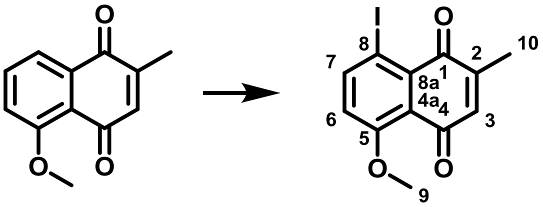

The product was obtained by the general microwave procedure described above. The reaction was conducted for 2h and purification by FCC (toluene/EtOAc 97:3) afforded iodinated product **2c** (28.9 mg, 88% yield) as red crystals; **m.p. (°C) =** 163.5-163.9

(Petrol/CH_2Cl_2); **IR (solid, cm^{-1}) *ν*:** 2978 (w), 1647 (s), 1223 (m), 962 (m); **HRMS (ESI$^+$):** 350.9492 $[M+Na]^+$. Cald. for $[C_{12}H_9IO_3Na]^+$: 350.9489; **^{1}H NMR (400 MHz, $CDCl_3$) *δ*:** 8.17 (d, *J* = 9.0 Hz, C**7**-H), 6.91 (d, *J* = 9.0 Hz, C**6**-H), 6.63 (s, C**3**-H), 3.93 (s, C**9**-H_3), 2.11 (s, C**10**-H_3); **^{13}C NMR (100 MHz, $CDCl_3$) *δ*:** 184.2 (C**1**), 182.9 (C**4**), 160.1 (C**5**), 148.4 (C**7**), 146.1 (C**2**), 136.4 (C**3**), 132.7 (C**8a**), 122.3 (C**4a**), 118.6 (C**6**), 81.8 (C**8**-**I**), 56.7 (C**9**), 16.3 (C**10**). *The structure of product **2c** was confirmed by X-ray diffraction, as shown below.*

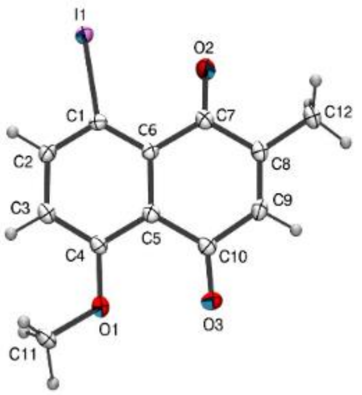

Figure 7: Crystal structure of compound **2c**.

Benzyl (4-iodo-5,8-dioxo-5,8-dihydronaphthalen-1-yl)carbamate (2d)

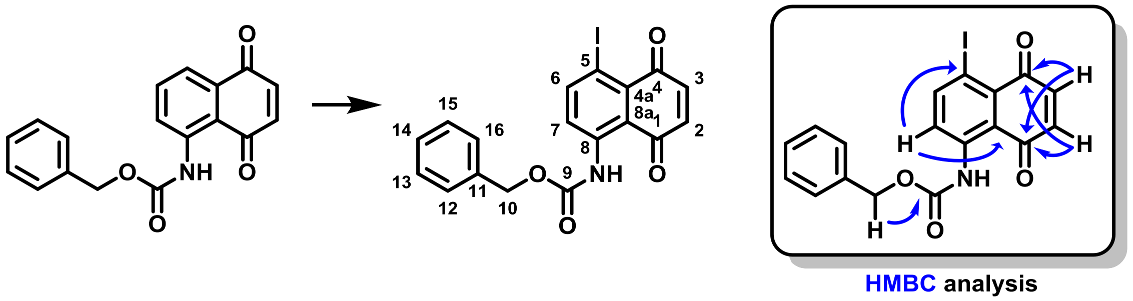

The product was obtained by the general microwave procedure described above. The reaction was conducted for 3h and purification by FCC (toluene) afforded iodinated product **2d** (29.9 mg, 69% yield) as a red powder; **m.p (°C)** = 121.4-122.5 (Petrol/CH_2Cl_2); **IR (solid, cm^{-1}) *ν*:** 2922 (w), 1679 (s), 1540 (m), 1311 (m); **HRMS (EI$^+$):** 432.9820 $[M]^+$. Cald. for $[C_{18}H_{12}NIO_4]$: 432.9811; **^{1}H NMR (500 MHz, $CDCl_3$) *δ*:** 11.74 (s, N-H), 8.53 (d, *J* = 9.2 Hz, C**7**-H), 8.29 (d, *J* = 9.2 Hz, C**6**-H), 7.52-7.30 (m, C**12**,**13**,**14**,**15**,**16**-H), 6.98 (d, *J* = 10.2 Hz, C**2**-H), 6.86 (d, *J* = 10.2 Hz, C**3**-H), 5.25 (s, C-H_2); **^{13}C NMR (125 MHz, $CDCl_3$) *δ*:** 187.7 (C**4**), 183.0 (C**1**), 153.5 (C**9**), 149.5 (C**6**), 142.8 (C**8**), 138.4 (C**2**), 138.3 (C**3**), 135.6 (C**11**) , 131.1 (C**4a**), 128.6 (C**12**, C**16**),

128.5 (C**14**), 128.4 (C**13**, C**15**), 124.9 (C**7**), 118.0 (C**8a**), 85.4 (C**5**-I), 67.5 (C**10**), *The structural assignment of this product was supported by HMBC analysis, as indicated above.*

5-Iodo-8-methylcarboxylate-1,4-naphthoquinone (2e)

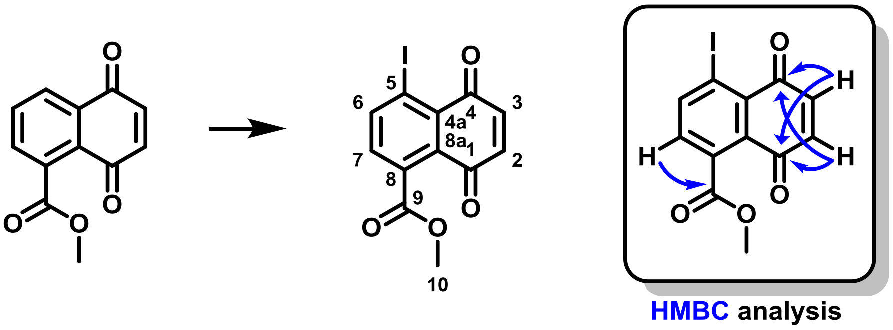

The product was obtained by the general microwave procedure described above, with minor modifications [$AgNTf_2$ (27 mol %, 10.5 mg), $[RhCp^*Cl_2]_2$ (5 mol %, 3.1 mg)]. The reaction was conducted for 8h and purification by FCC (toluene) afforded iodinated product **2e** (19.5 mg, 57% yield) as an orange powder; **m.p. (°C)** = 123.9-124.7 (Petrol/CH_2Cl_2); **IR (solid, cm^{-1}) *ν*:** 2922 (w), 1731 (s), 1667 (s), 1322 (m), 1078 (s); **HRMS (EI$^+$):** 341.9388 $[M]^+$. Cald. for $[C_{12}H_7IO_4]$: 341.9389; **^{1}H NMR (400 MHz, $CDCl_3$) *δ*:** 8.42 (d, *J* = 8.2 Hz, C**6**-H), 7.27 (d, *J* = 8.2 Hz, C**7**-H), 7.05 (d, *J* = 10.3 Hz, C**3**-H), 6.95 (d, *J* = 10.3 Hz, C**2**-H), 3.97 (s, C**10**-H_3); **^{13}C NMR (100 MHz, $CDCl_3$) *δ*:** 182.8 (C**1**), 182.4 (C**4**), 169.0 (C**9**), 148.0 (C**6**), 139.4 (C**3**), 137.2 (C**2**), 134.9 (C**8**), 131.9 (C**7**), 131.4 (C**8a**), 131.0 (C**4a**), 94.1 (C**5**-I), 53.2 (C**10**). *The structural assignment of this product was supported by HMBC analysis, as indicated above.*

5-Iodo-8-nitro-1,4-naphthoquinone (2f)

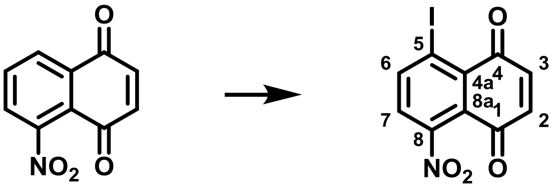

The product was obtained by the general microwave procedure described above, with minor modifications [$AgNTf_2$ (27 mol %, 10.5 mg), $[RhCp^*Cl_2]_2$ (5 mol %, 3.1 mg)]. The reaction was conducted for 8h and purification by FCC (toluene) afforded iodinated

product **2f** (7.89 mg, 24% yield) as deep orange crystals; **m.p. (°C)** = 174.6-175.5 (Petrol/CH_2Cl_2); **IR (solid, cm^{-1}) *v*:** 2922 (w), 1679 (s), 1540 (m), 1311 (m); **HRMS (EI$^+$):** 328.9190 $[M]^+$. Cald. for $[C_{10}H_4NIO_4]$: 328.9185; **^{1}H NMR (400 MHz, $CDCl_3$) *δ*:** 8.55 (d, *J* = 8.4 Hz, C**6**-H), 7.36 (d, *J* = 8.4 Hz, C**7**-H), 7.12 (d, *J* = 10.2 Hz, C**3**-H)), 7.02 (d, *J* = 10.2 Hz, C**2**-H); **^{13}C NMR (100 MHz, $CDCl_3$) *δ*:** 181.3 (C**4**), 180.3 (C**1**), 149.0 (C**6**), 139.2 (C**3**), 137.5 (C**2**), 133.9 (C**8**), 131.4 (C**4a**), 127.1 (C**7**), 125.2 (C**8a**), 95.2 (C**5**-I). *The structure of product **2f** was confirmed by X-ray diffraction, as shown below.*

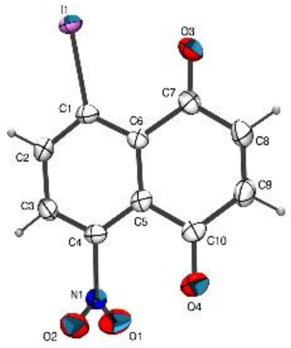

Figure 8: Crystal structure of compound **2f**.

5-Iodo-6-methoxy-1,4-naphthoquinone (2g)

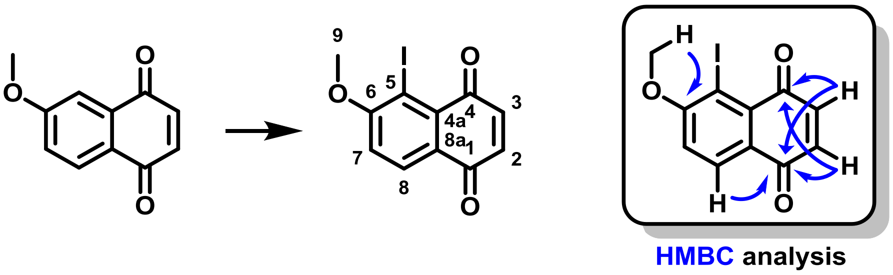

The product was obtained by the general microwave procedure described above. The reaction was conducted for 2h and purification via by FCC (toluene) afforded iodinated product **2g** (23.6 mg, 75% yield) as orange crystals; **m.p. (°C)** = 213.6-214.8 (Petrol/CH_2Cl_2); **IR (solid, cm^{-1}) *v*:** 2988 (w), 1662 (s), 1279 (m), 1020 (m); **HRMS (ESI$^+$):** 336.9344 $[M+Na]^+$. Cald. for $[C_{11}H_7IO_3Na]^+$: 336.9332; **^{1}H NMR (400 MHz, $CDCl_3$) *δ*:** 8.16 (d, *J* = 8.6 Hz, C**8**-H), 7.12 (d, *J* = 8.6 Hz, C**7**-H), 6.99 (d, *J* = 10.2 Hz, C**3**-H), 6.88 (d, *J* = 10.2 Hz, C**2**-H), 4.03 (s, C**9**-H_3); **^{13}C NMR (101 MHz, $CDCl_3$) *δ*:** 184.0 (C**4**), 183.0 (C**1**), 163.2 (C**6**), 139.7 (C**3**), 136.9 (C**2**), 133.0 (C**4a)**, 129.9 (C**8**),

127.8 (C**8a**), 113.6 (C**7**), 87.2 (C**5**-I), 57.2 (C**9**). *The structural assignment of this product was supported by HMBC analysis, as indicated above.*

5,8-Diiodo-6-methoxy-1,4-naphthoquinone

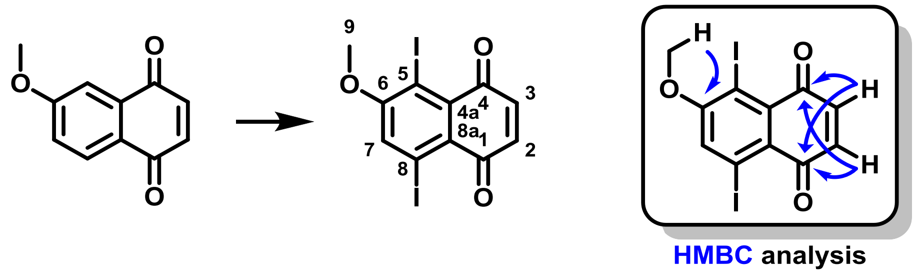

In addition to **2g**, a C5/8 bis-iodinated by-product (1.8 mg, 4% yield) was obtained as a brown powder; **m.p (°C)** = 244.3-245.5 (Petrol/CH_2Cl_2); **IR (solid, cm^{-1}) *ν*:** 3337 (w), 1657 (s), 1307 (m), 1048 (m); **HRMS (EI$^+$):** 439.8403. Cald. for [$C_{11}H_6I_2O_3$]: 439.8406; **^{1}H NMR (500 MHz, $CDCl_3$) *δ*:** 7.71 (s, C**7**-H), 6.99 (d, *J* = 10.2 Hz, C**2**-H), 6.93 (d, *J* = 10.2 Hz, C**3**-H), 4.05 (s, C**9**-H_3); **^{13}C NMR (125 MHz, $CDCl_3$) *δ*:** 183.3 (C**1**), 181.5 (C**4**), 161.8 (C**6)**, 138.0 (C**3**), 137.9 (C**2**), 134.9 (C**4a**), 127.9 (C**7**), 126.2 (C**8a**), 95.2 (C**8**-I), 89.2 (C**5**-I), 57.5 (C**9**). *The structural assignment of this product was supported by HMBC analysis, as indicated above.*

5-Iodo-7-methyl-1,4-naphthoquinone (2h)

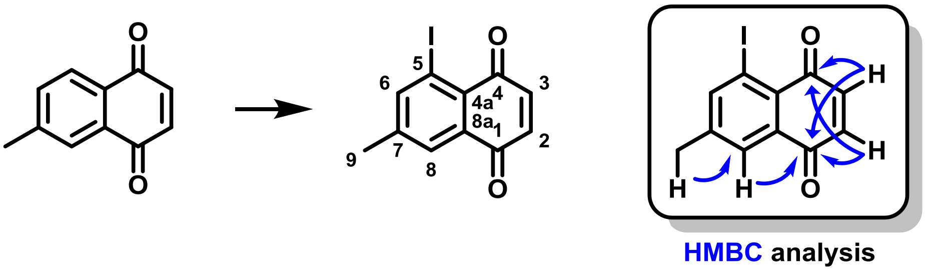

The product was obtained by the general microwave procedure described above. The reaction was conducted for 3h and purification by FCC (toluene) afforded iodinated product **2h** (18.8 mg, 63% yield) as a deep orange powder; **m.p. (°C)** = 127.1-128.4 (Petrol/CH_2Cl_2); **IR (solid, cm^{-1}) *ν*:** 2923 (w), 1665 (s), 1313 (m), 844 (m); **HRMS (EI$^+$):** 297.9491 [M]$^+$. Cald. for [$C_{10}H_5IO_2$]: 297.9492; **^{1}H NMR (400 MHz, $CDCl_3$) *δ*:** 8.17 (d, *J* = 1.8 Hz, C**6**-H), 7.92 (d, *J* = 1.8 Hz, C**8**-H), 6.97 (d, *J* = 10.3 Hz, C**3**-H),

6.89 (d, J = 10.3 Hz, C**2**-H), 2.42 (s, C**9**-H_3); **^{13}C NMR (101 MHz, $CDCl_3$) *δ*:** 183.9 (C**1**), 182.9 (C**4**), 148.6 (C**6**), 145.1 (C**7**), 139.8 (C**3**), 136.9 (C**2**), 133.9 (C**8a**), 128.4 (C**4a**), 128.2 (C**8**), 93.0 (C**5**-I), 21.1 (C**9**). *The structural assignment of this product was supported by HMBC analysis, as indicated above.*

5,8-Diiodo-6-methyl-1,4-naphthoquinone

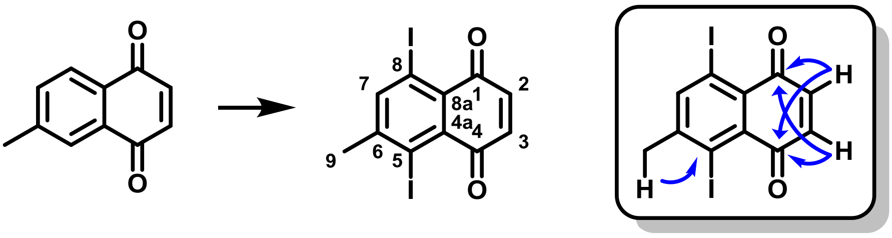

In addition to **2h**, a C5/8 bis-iodinated by-product (1.3 mg, 3% yield) was obtained as a deep orange powder; **m.p. (°C)** = 178.2-179.5 (Petrol/CH_2Cl_2); **IR (solid, cm^{-1}) *ν*:** 2987 (w), 1663 (s), 1047 (m), 854 (m); **HRMS (EI$^+$):** 423.8456 $[M]^+$. Cald. for $[C_{11}H_6I_2O_2]$: 423.8457; **^{1}H NMR (500 MHz, $CDCl_3$) *δ*:** 8.24 (s, C**7**-H), 6.97 (d, J = 10.2 Hz, C**3**-H), 6.93 (d, J = 10.2 Hz, C**2**-H), 2.61 (s, C**9**-H_3); **^{13}C NMR (125 MHz, $CDCl_3$) *δ*:** 183.6 (C**4**), 182.2 (C**1**), 151.2 (C**6**), 147.3 (C**7**), 138.2 (C**3**), 137.5 (C**2**), 134.1 (C**4**a), 130.7 (C**8**a), 101.5 (C**5**-I), 93.1 (C**8**-I), 30.6 (C**9**). *The structural assignment of this product was supported by HMBC analysis, as indicated above.*

5-Iodo-6,7-dimethoxy-1,4-naphthoquinone (2i)

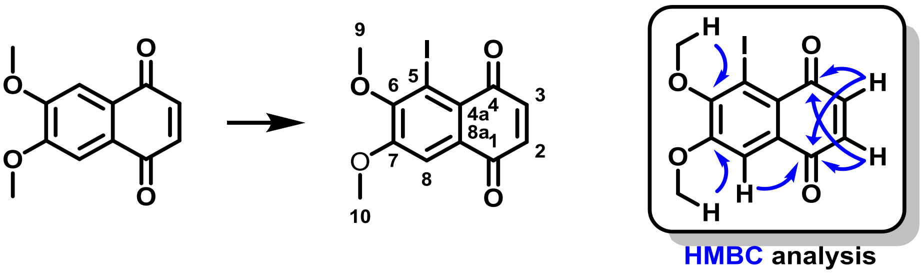

The product was obtained by the general microwave procedure described above. The reaction was conducted for 3h and purification by FCC (toluene) afforded iodinated product **2i** (23.1 mg, 67% yield) as an orange powder; **m.p (°C)** = 167.4-168.6 (Petrol/CH_2Cl_2); **IR (solid, cm^{-1}) *ν*:** 2924 (w), 1567 (s), 1316 (m), 1035 (m); **HRMS**

(ESI$^+$): 366.9438 [M+Na]$^+$. Cald. for [$C_{12}H_9IO_4Na$]$^+$: 366.9450; **^{1}H NMR (400 MHz, CDCl$_3$) δ:** 7.64 (s, C**8**-H), 6.94 (d, *J* = 10.2 Hz, C**3**-H), 6.86 (d, *J* = 10.2 Hz, C**2**-H), 4.02 (s, C**10**-H_3), 3.90 (s, C**9**-H_3); **^{13}C NMR (100 MHz, CDCl$_3$) δ:** 183.4 (C**1**), 182.7 (C**4**), 155.6 (C**7**), 154.2 (C**6**), 140.1 (C**3**), 136.2 (C**2**), 131.6 (C**8a**), 125.4 (C**4a**), 110.5 (C**8**), 93.6 (C**5**-I), 60.6 (C**9**), 56.5 (C**10**). *The structural assignment of this product was supported by HMBC analysis, as indicated above.*

2-Bromo-5-iodo-1,4-naphthoquinone (2j)

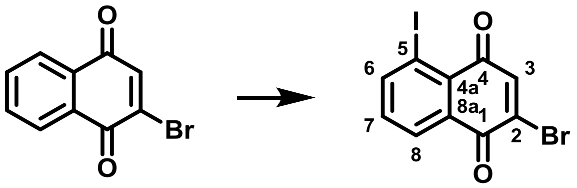

The product was obtained by the general microwave procedure described above. The reaction was conducted for 2h and purification by FCC (toluene) afforded iodinated product **2j** (21.4 mg, 59% yield) as orange crystals; **m.p (°C)** = 159.5-160.6 (Petrol/CH_2Cl_2); **IR (solid, cm^{-1}) *ν*:** 2923 (w), 1677 (s), 1232 (m), 1062 (m); **HRMS (EI$^+$):** 361.8454 [M]$^+$. Cald. for [$C_{10}H_5IO_2$]: 361.8439; **^{1}H NMR (400 MHz, CDCl$_3$) δ:** 8.40 (d, *J* = 7.2 Hz, C**6**-H), 8.25 (d, *J* = 7.7 Hz, C**8**-H), 7.55 (s, 1H), 7.38 (t, J = 7.8 Hz, C**7**-H); **^{13}C NMR (101 MHz, CDCl$_3$) δ:** 180.5 (C**4**), 177.0 (C**1**), 148.7 (C**6**), 141.2 (C**3**), 137.9 (C**2**), 133.8 (C**7**), 133.6 (C**8a**), 130.5 (C**4a**), 128.9 (C**8**), 93.3 (C**5**-I). *The structure of product **2j** was confirmed by X-ray diffraction, as shown below.*

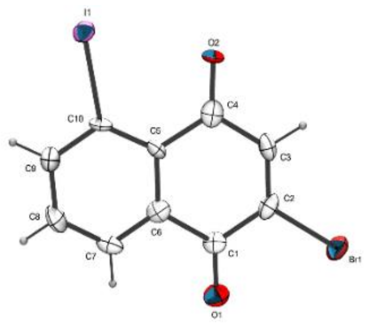

Figure 9: Crystal structure of compound **2j**.

2,3-Dibromo-5-iodo-1,4-naphthoquinone (2k)

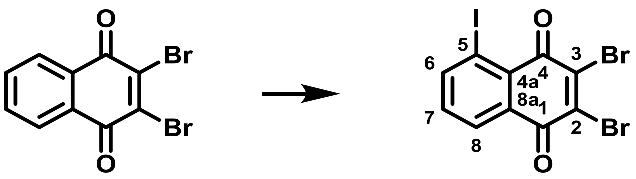

The product was obtained by the general microwave procedure described above, with minor modifications [$AgNTf_2$ (27 mol %, 10.5 mg), $[RhCp^*Cl_2]_2$ (5 mol %, 3.1 mg), 65°C and 75W]. The reaction was conducted for 4h and purification by FCC (toluene) afforded iodinated product **2k** (26.9 mg, 61% yield) as orange crystals; **m.p. (°C)**: 185.2-186.8 (Petrol/CH_2Cl_2); **IR (solid, cm^{-1}) *ν*:** 2923 (w), 1674 (s), 1231 (m), 719 (m); **HRMS (EI$^+$):** 439.7540. Cald. for [$C_{10}H_3IBr_2O_2$]: 439.7545; **^{1}H NMR (400 MHz, $CDCl_3$) *δ*:** 8.42 (d, *J* = 7.9 Hz, C**6**-H), 8.26 (d, *J* = 7.7 Hz, C**8**-H), 7.38 (t, *J* = 7.8 Hz, C**7**-H); **^{13}C NMR (100 MHz, $CDCl_3$) *δ*:** 174.9 (C**1**), 174.1 (C**4**), 148.9 (C**6**), 143.2 (C**3**), 140.7 (C**2**), 134.1 (C**7**), 133.3 (C**8a**), 130.1 (C**4a**), 129.3 (C**8**), 94.9 (C**5**-I). *The structure of product **2k** was confirmed by X-ray diffraction, as shown below.*

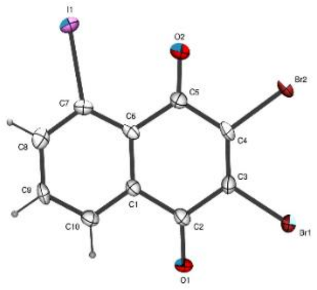

Figure 10: Crystal structure of compound **2k**.

2,3-Dichloro-5-iodo-1,4-naphthoquinone (2l)

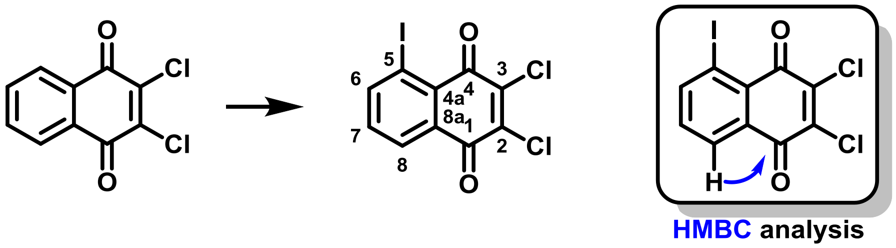

The product was obtained by the general microwave procedure described above, with minor modifications [$AgNTf_2$ (27 mol %, 10.5 mg), $[RhCp^*Cl_2]_2$ (5 mol %, 3.1 mg)].

The reaction was conducted for 8h and purification by FCC (toluene) afforded iodinated product **2l** (20.3 mg, 51% yield) as orange crystals; **m.p. (°C)** = 194.2-195.6 (Petrol/CH_2Cl_2); **IR (solid, cm^{-1}) *ν*:** 3070 (w), 1676 (s), 1149 (m), 723 (m); **HRMS (EI$^+$):** 351.8555 $[M]^+$. Cald. for [$C_{10}H_5IO_2$]: 351.8538; **^{1}H NMR (500 MHz, CDCl$_3$) *δ*:** 8.44 (d, *J* = 7.9 Hz, C**6**-H), 8.28 (d, *J* = 7.7 Hz, C**8**-H), 7.41 (t, *J* = 7.8 Hz, C**7**-H); **^{13}C NMR (125 MHz, CDCl$_3$) *δ*:** 175.0 (C**1**), 174.2 (C**4**), 149.1 (C**6**), 144.0 (C**3**), 142.1 (C**2**), 134.2 (C**7**), 133.5 (C**8a**), 130.1 (C**4a**), 128.8 (C**8**), 94.5 (C**5**-I). *The structural assignment of this product was supported by HMBC analysis, as indicated above.*

5-Bromo-1,4-naphthoquinone (6)

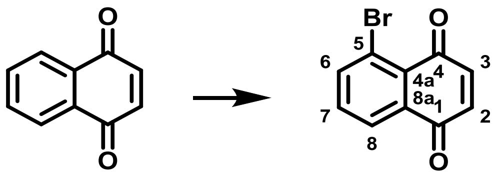

The product was obtained by the general microwave procedure described above, with minor modifications [1,3-dibromo-5,5-dimethylhydantoin (DBH) (150 mol %, 0.15 mmol, 42.9 mg), 65°C and 75W]. The reaction was conducted for 5h and purification by FCC (toluene) afforded brominated product **6** (12.1 mg, 51% yield) as yellow crystals; **m.p. (°C)**: 153.9-154.8 (Petrol/CH_2Cl_2); **IR (solid, cm^{-1}) *ν*:** 3069 (w), 1667 (s), 1319 (m), 779 (m); **HRMS (EI$^+$):** 235.9479 $[M]^+$. Cald. for [$C_{10}H_5BrO_2$]: 235.9473; **^{1}H NMR (400 MHz, CDCl$_3$) *δ*:** 8.12 (dd, *J* = 8.0, 1.3 Hz, C**8**-H), 7.99 (dd, J = 8.1, 1.3 Hz, C**6**-H), 7.54 (t, *J* = 8.0 Hz, C**7**-H), 6.99 (d, *J* = 10.3 Hz, C**3**-H), 6.94 (d, J = 10.3 Hz, C**2**-H); **^{13}C NMR (100 MHz, CDCl$_3$) *δ*:** 183.7 (C**1**), 183.3 (C**4**), 141.0 (C**6**), 140.1 (C**3**), 136.7 (C**2**), 134.6 (C**8a**), 133.7 (C**7**), 129.0 (C**4a**), 126.7 (C**8**), 121.9 (C**5**-Br). *The structure of product* ***6*** *was confirmed by X-ray diffraction, as shown below.*

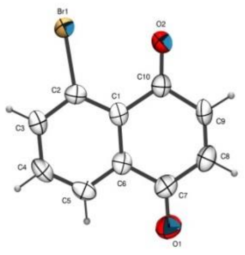

Figure 11: Crystal structure of compound **6**.

General thermal procedure for halogenation at the 2-position:

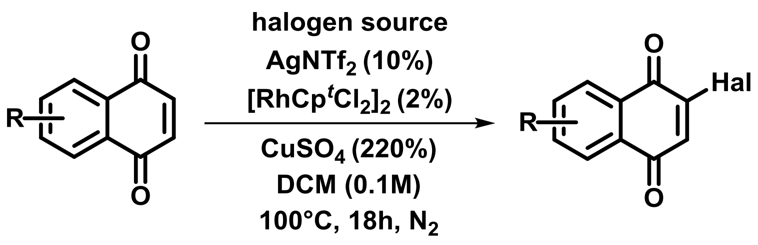

In a glovebox, oven dried re-sealable tube was charged with the corresponding naphthoquinone (0.10 mmol), $[RhCp^tCl_2]_2$ (2 mol %, 1.4 mg), silver bis(trifluoromethanesulfonyl)imide (10 mol %, 3.9 mg), the halogen source (see below) and anhydrous copper sulphate (220 mol %, 0.22 mmol, 34.9 mg). The tube was removed from the glovebox and an inert atmosphere was maintained. Anhydrous CH_2Cl_2 (1 mL) was added via syringe and tube was sealed. The mixture was heated at 100 °C for 18h. After cooling, the mixture was filtered through a pad of celite and purified by FCC, under the conditions noted.

2-Bromo-1,4-naphthoquinone (7)

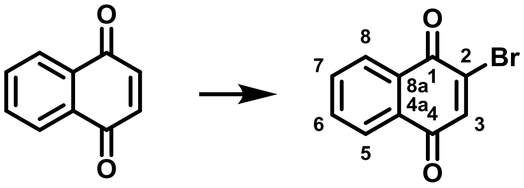

The product was obtained by the general thermal procedure described above using 1,3-dibromo-5,5-dimethylhydantoin (DBH) (150 mol %, 0.15 mmol, 42.9 mg) and a reaction time of 18h. Purification by FCC (hexane/ethyl acetate 98:2) afforded brominated product **7** (21 mg, 88% yield) as light yellow crystals; **m.p. (°C) =** 130.8-131.6 (Petrol/CH_2Cl_2); **^{1}H NMR (400 MHz, $CDCl_3$) *δ*:** 8.16 (dd, *J* = 5.9, 3.1 Hz, 1H), 8.07 (dd, J = 5.9, 3.0 Hz, 1H), 7.83 – 7.70 (m, 2H), 7.51 (s, 1H); **^{13}C NMR (100 MHz, $CDCl_3$) *δ*:** 182.4, 177.8, 140.3, 140.1, 134.4, 134.1, 131.7, 130.9, 127.8, 126.9. Data are consistent with those reported in the literature.[14]

2-Iodo-1,4-naphthoquinone (3a)

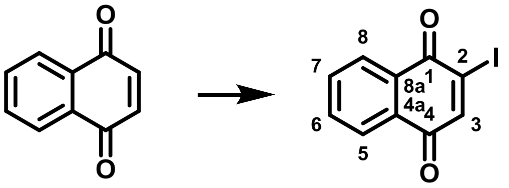

The product was obtained by the general thermal procedure described above using 1,3-diiodo-5,5-dimethylhydantoin (DIH) (100 mol %, 0.10 mmol, 37.9 mg) and a reaction time of 18h. Purification by FCC (hexane/ethyl acetate 99:1) afforded iodinated product **3a** (26 mg, 90% yield) as orange crystals; **m.p. (°C):** 111.4-112.9 (Petrol/CH_2Cl_2); **IR (solid, cm^{-1}) *ν*:** 3043 (w), 1671 (s), 1652 (s), 1320 (m); **HRMS (EI^+):** 283.9326 $[M]^+$. Cald. for [$C_{10}H_5IO_2$]: 283,9334; **^1H NMR (400 MHz, $CDCl_3$) *δ*:** 8.18 (d, *J* = 7.0 Hz, C**8**-H), 8.09 (d, *J* = 9.1 Hz, C**5**-H), 7.91 (s, C**3**-H), 7.83 – 7.72 (m, C**7**-H, C**6**-H); **^{13}C NMR (100 MHz, $CDCl_3$) *δ*:** 182.0 (C**4**), 178.8 (C**1**), 148.4 (C**3**), 134.3 (C**6**), 134.0 (C**7**), 131.7 (C**4a**), 129.7 (C**8a**), 128.2 (C**8**), 127.0 (C**5**), 123.0 (C**2**-I). *The structure of product **3a** was confirmed by X-ray diffraction, as shown below.*

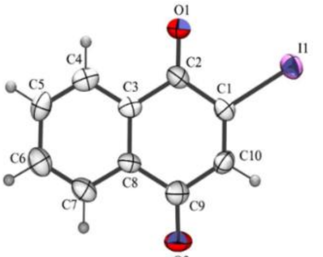

Figure 12: Crystal structure of compound **3a**.

2-Iodo-7-methyl-1,4-naphthoquinone and 2-Iodo-6-methyl-1,4-naphthoquinone (3h)

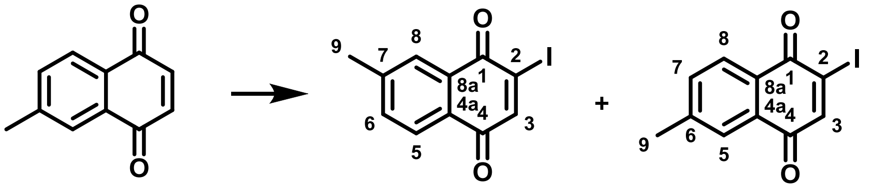

a **b**

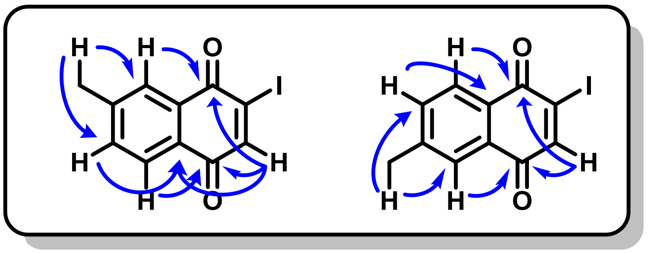

HMBC analysis

The products were obtained by the general thermal procedure described above using 1,3-diiodo-5,5-dimethylhydantoin (DIH) (100 mol %, 0.10 mmol, 37.9 mg) and a reaction time of 18h. Purification by FCC (hexane/ethyl acetate 99:1) afforded iodinated product **3h** as a mixture of isomers **a** and **b** (23.0 mg, 81% yield, 1.3:1 **a:b**) as a yellow powder; **HRMS (EI$^+$):** 297.9477 [M]$^+$. Cald. for [$C_{11}H_7IO_2$]: 297.9491; **^{1}H NMR (400 MHz, $CDCl_3$) *δ*:** 8.05 (d, *J* = 7.9 Hz, C**8**-H for **b**), 7.99 – 7.92 (m, C**5**-H, C**8**-H for **a**), 7.87 – 7.83 (m, C**5**-H, C**3**-H for **b** + C**3**-H for **a**), 7.56 (d, *J* = 7.8 Hz, C**6**-H for **a**), 7.52 (d, *J* = 8.2 Hz, C**7**-H for **b**), 2.50 (s, C**9**-H_3 for **b** + C**9**-H_3 for **a**); **^{13}C NMR (100 MHz, $CDCl_3$) *δ*:** 182.3 (C**4**, **b**), 181.9 (C**4**, **a**), 179.0 (C**1**, **a**), 178.5 (C**1**, **b**), 148.5 (C**3**, **a**), 148.2 (C**3**, **b**), 145.7 (C**7**, **a**), 145.3 (C**7, b**), 135.0 (C**6**, **a**), 134.7 (C**6**, **b**), 131.6 (C**5**, **b**), 129.6 (C**4a**, **a**), 129.5 (C**4a**, **b**), 128.5 (C**8**, **a**), 128.4 (C**8**, **b**), 127.4 (C**8a** for **b**, C**8a** for **a**), 127.2 (C**5**, a), 123.4 (C**2**-I, b), 122.7 (C**2**-I, **a**) , 21.9 (C**9**, **b** and **a**). *Structural assignments of both products were supported by HMBC analysis, as indicated above.*

Procedures for derivatization reactions:

5-Phenyl-1,4-naphthoquinone (5a)

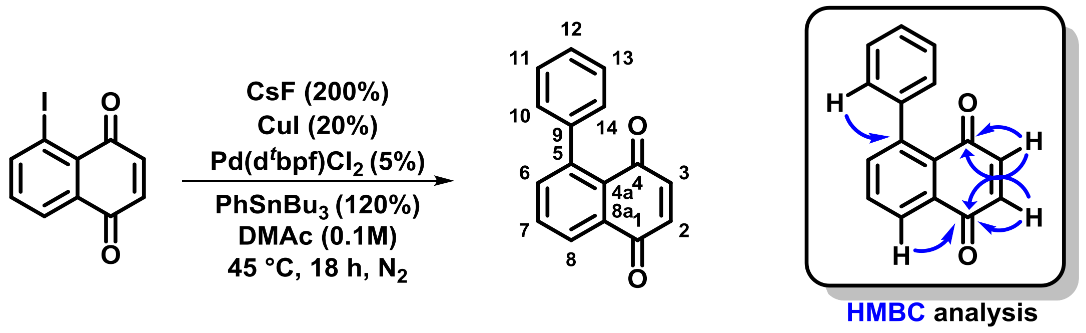

A oven dried re-sealable tube was charged with **2a** (0.10 mmol, 28.4 mg), Pd(d^{t}bpf)Cl$_2$ (5 mol %, 3.7 mg), CuI (20 mol %, 4.0 mg) and CsF (0.20 mmol, 30.0 mg). The tube was purged with N_2 and PhSnBu$_3$ (0.12 mmol, 40.0 µL) and *N*,*N*-dimethylacetamide (1 mL) were added via syringe. The tube was sealed and the mixture was heated at 45 °C for 18h. After cooling, the solvent was removed under reduced pressure. The residue was dissolved in CH_2Cl_2 (5 mL) and filtered through a pad of celite. The filtrate was concentrated under reduced pressure and the residue was purified by FCC (hexane/EtOAc 5:1) to afford **5a** (17.6 mg, 75% yield) as an orange powder; **m.p. (°C)** = 165.5-166.9 (Petrol/CH_2Cl_2); **IR (solid, cm^{-1}) *ν*:** 3050 (w), 1658 (s), 1279 (s), 698 (s); **HRMS (EI$^+$):** 234.0672 [M]$^+$. Cald. for [$C_{16}H_{10}O_2$]: 234.0681; **^{1}H NMR (400 MHz, CDCl$_3$) *δ*:** 8.17 (dd, *J* = 7.8, 1.3 Hz, C**8**-H), 7.73 (t, *J* = 7.8 Hz, C**7**-H), 7.56 (dd, *J* = 7.8, 1.3 Hz, C**6**-H), 7.48 – 7.36 (m, C**11**-H, C**12**-H, C**13**-H), 7.29 – 7.21 (m, C**10**-H, C**14**-H), 6.94 (d, *J* = 10.3 Hz, C**2**-H), 6.82 (d, *J* = 10.3 Hz, C**3**-H); **^{13}C NMR (100 MHz, CDCl$_3$) *δ*:** 185.2 (C**4**), 185.1 (C**1**), 143.7 (C**5**), 141.0 (C**9**), 140.3 (C**3**), 137.5 (C**6**), 137.0 (C**2**), 133.2 (C**8a**), 132.8 (C**7**), 129.1 (C**4a**), 128.1 (C**10**, C**11**, C**13**, C**14**), , 127.4 (C**12**), 126.4 (C**8**). *The structural assignment of this product was supported by HMBC analysis, as indicated above.*

Ethyl (*E*)-3-(5,8-dioxo-5,8-dihydronaphthalen-1-yl)acrylate (5b)

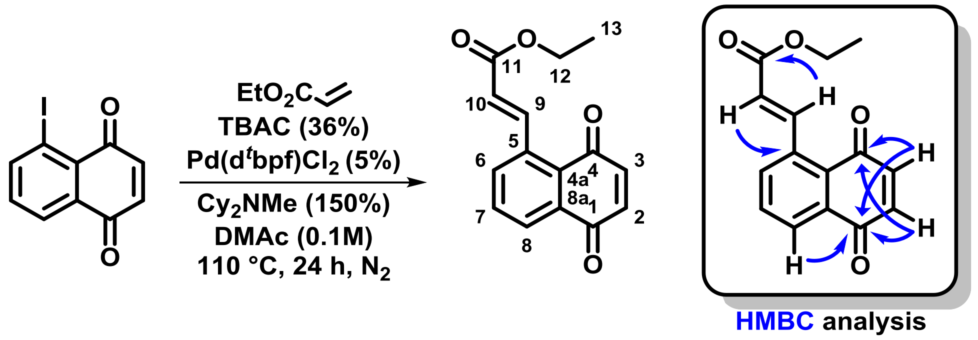

A oven dried re-sealable tube was charged with **2a** (0.10 mmol, 28.4 mg), Pd(d^{t}bpf)Cl_2 (5 mol %, 3.7 mg) and tetrabutylammonium chloride (36 mol %, 10.0 mg). The tube was purged with N_2 and ethyl acrylate (0.30 mmol, 32.6 µL), Cy_2NMe (150 mol %, 0.15 mmol, 32.0 µL) and N,N-dimethylacetamide (1.0 mL) were added via syringe. The tube was sealed and the mixture was heated at 110 °C for 24h. After cooling, the solvent was removed under reduced pressure. The residue was dissolved in CH_2Cl_2 (5 mL) and filtered through a pad of celite. The filtrate was concentrated and the residue was purified by FCC (hexane/EtOAc 5:1) to afford **5b** (17.8 mg, 66% yield) as a yellow powder; **m.p. (°C)** = 133.8-134.9 (Petrol/CH_2Cl_2); **IR (solid, cm^{-1}) *ν*:** 2909 (w), 1717 (s), 1654 (s), 1322 (m), 1193 (m), 773 (m); **HRMS (ESI$^+$):** 279.0629 [M+Na]$^+$. Cald. for $[C_{15}H_{12}NaO_4]^+$: 279.0628; **^{1}H NMR (400 MHz, $CDCl_3$) *δ*:** 8.59 (d, J = 15.9 Hz, C**9**-H), 8.17 (dd, J = 7.2, 1.9 Hz, C**8**-H), 7.87 – 7.68 (m, C**6**-H, C**7**-H), 6.96 (s, C**2**-H, C**3**-H), 6.27 (d, J = 15.9 Hz, C**10**-H), 4.30 (q, J = 7.1 Hz, C**12**-H_2), 1.36 (t, J = 7.1 Hz, C**13**-H_3). **^{13}C NMR (100 MHz, $CDCl_3$) *δ*:** 186.2 (C**4**), 184.6 (C**1**), 166.2 (C**11**), 144.0 (C**9**), 140.0 (C**3**) , 137.2 (C**2**), 137.2 (C**8a**), 134.1 (C**6**), 133.6 (C**7**), 133.1 (C**5**), 129.0 (C**4a**), 128.1 (C**8**), 122.8 (C**10**), 60.7 (C**12**), 14.3 (C**13**). *The structural assignment of this product was supported by HMBC analysis, as indicated above.*

5-(Phenylethynyl)-1,4-naphthoquinone (5c)

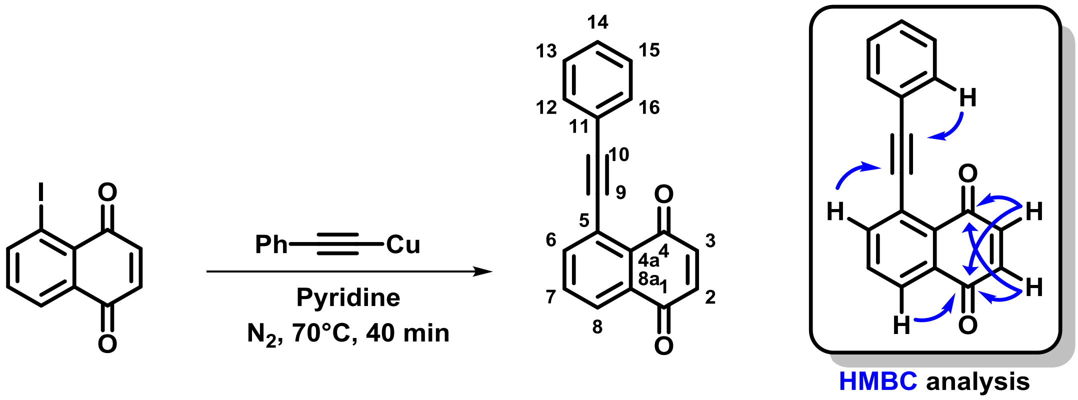

A oven dried re-sealable tube was charged with **2a** (28.4 mg, 0.10 mmol) and (phenylethynyl)copper (26.4 mg, 0.16 mmol). The tube was purged with N_2 and pyridine (1.0 mL) was added via syringe. The tube was sealed and the mixture was heated at 70 °C for 40 minutes. The mixture was cooled to room temperature and extracted with ether (10 mL). The organic extract were combined, dried with anhydrous Na_2SO_4 and concentrated *in vacuo*. Purification of the residue by FCC (toluene) afforded **5c** (16.5 mg, 64% yield) as a green powder; **m.p. (°C)** = 101.8-102.9 (Petrol/CH_2Cl_2); **IR (solid, cm^{-1}) *ν*:** 3059 (w), 1667 (s), 1289 (m), 756 (m); **HRMS (EI$^+$):** 258.0685 $[M]^+$. Cald. for $[C_{18}H_{10}IO_2]$: 258.0681; **^{1}H NMR (400 MHz, $CDCl_3$) *δ*:** 8.10 (d, *J* = 7.8 Hz, C**8**-H), 7.93 (d, *J* = 7.8 Hz, C**6**-H), 7.76 – 7.65 (m, C**12**-H, C**16**-H, C**7**-H), 7.45 – 7.34 (m, (C**13**-H, C**14**-H, C**15**-H), 7.00 (d, *J* = 10.3 Hz, C**3**-H), 6.96 (d, *J* = 10.3 Hz, C**2**-H).; **^{13}C NMR (101 MHz, $CDCl_3$) *δ*:** 184.6 (C**1**), 183.8 (C**4**), 139.9 (C**3**), 139.7 (C**6**), 137.2 (C**2**), 132.9 (C**8a**), 132.7 (C**7**), 132.1 (C**12**, C**16**), 131.4 (C**4a**), 129.0 (C**14**), 128.4 (C**13**, C**15**), 126.6 (C**8**), 123.0 (C**5**), 123.0 (C**11**), 95.8 (C**10**), 88.3 (C**9**). *The structural assignment of this product was supported by HMBC analysis, as indicated above.*

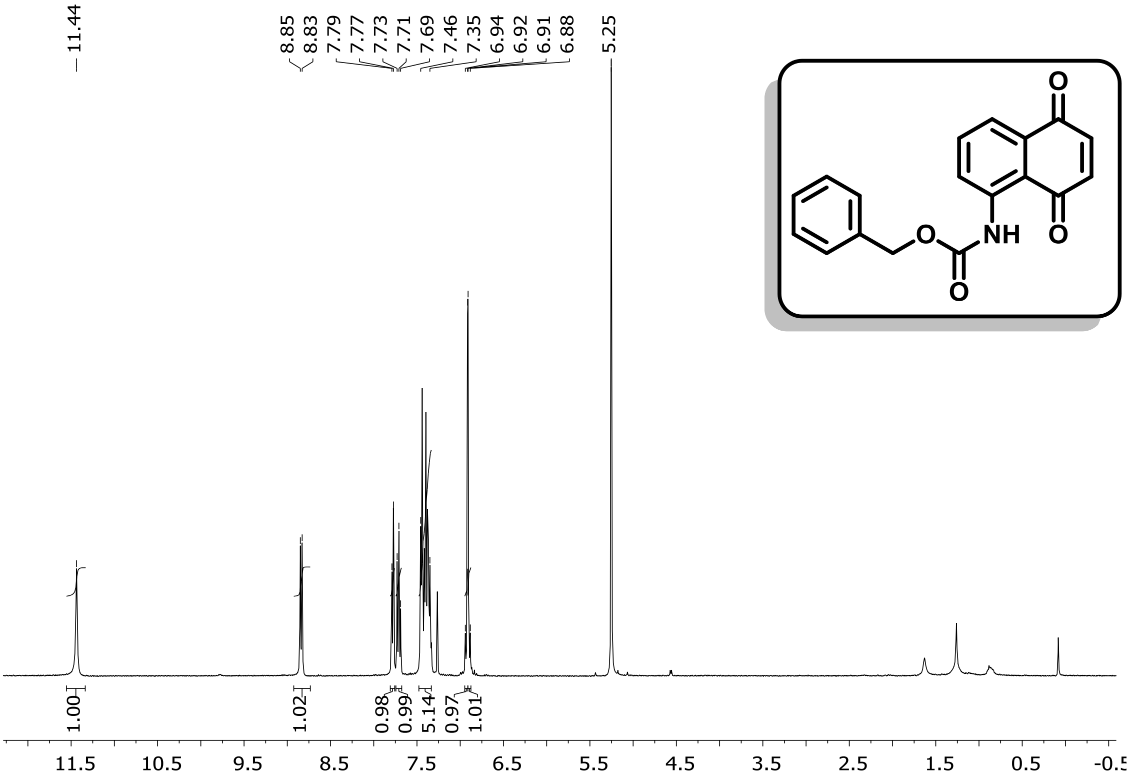
11.44
8.85
8.83
7.79
7.77
7.73
7.71
7.69
7.46
7.35
6.94
6.92
6.91
6.88
5.25
1.00
1.02
0.98
0.99
5.14
0.97
1.01
11.5 10.5 9.5 8.5 7.5 6.5 5.5 4.5 3.5 2.5 1.5 0.5
O
O
O
NH
O

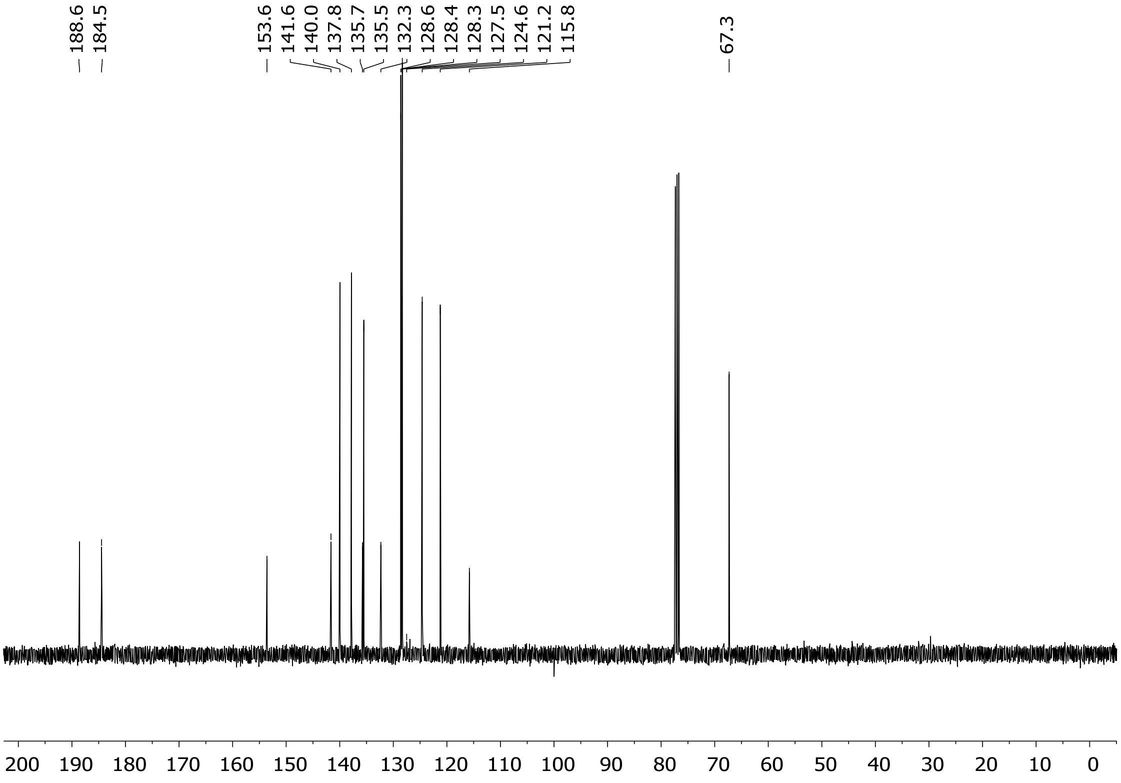
188.6
184.5
153.6
141.6
140.0
137.8
135.7
135.5
132.3
128.6
128.4
128.3
127.5
124.6
121.2
115.8
67.3
200 190 180 170 160 150 140 130 120 110 100 90 80 70 60 50 40 30 20 10 0

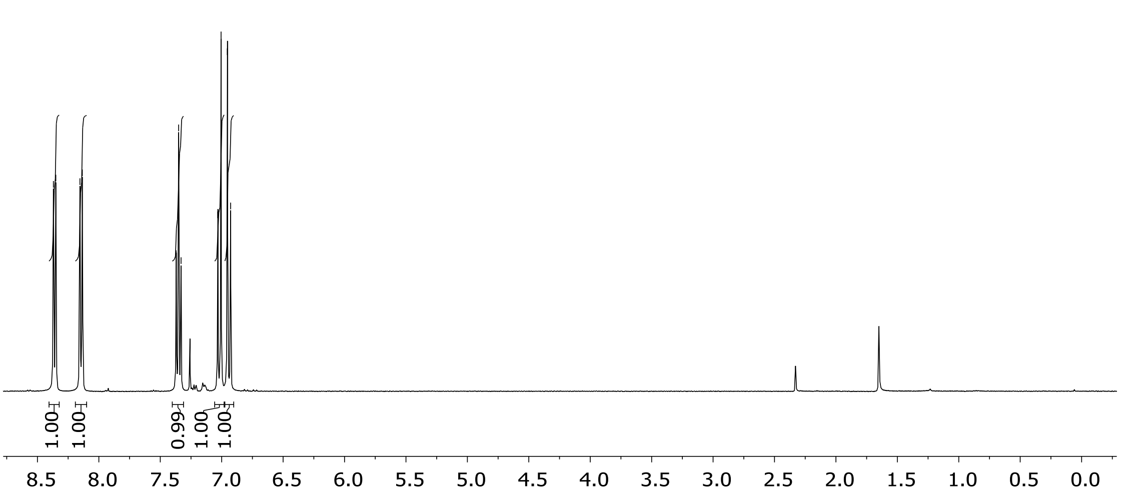
8.37
8.35
8.15
8.13
7.37
7.35
7.33
7.03
7.01
6.95
6.93
1.00
1.00
0.99
1.00
1.00
8.5 8.0 7.5 7.0 6.5 6.0 5.5 5.0 4.5 4.0 3.5 3.0 2.5 2.0 1.5 1.0 0.5 0.0

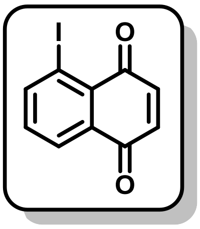
I
O
O

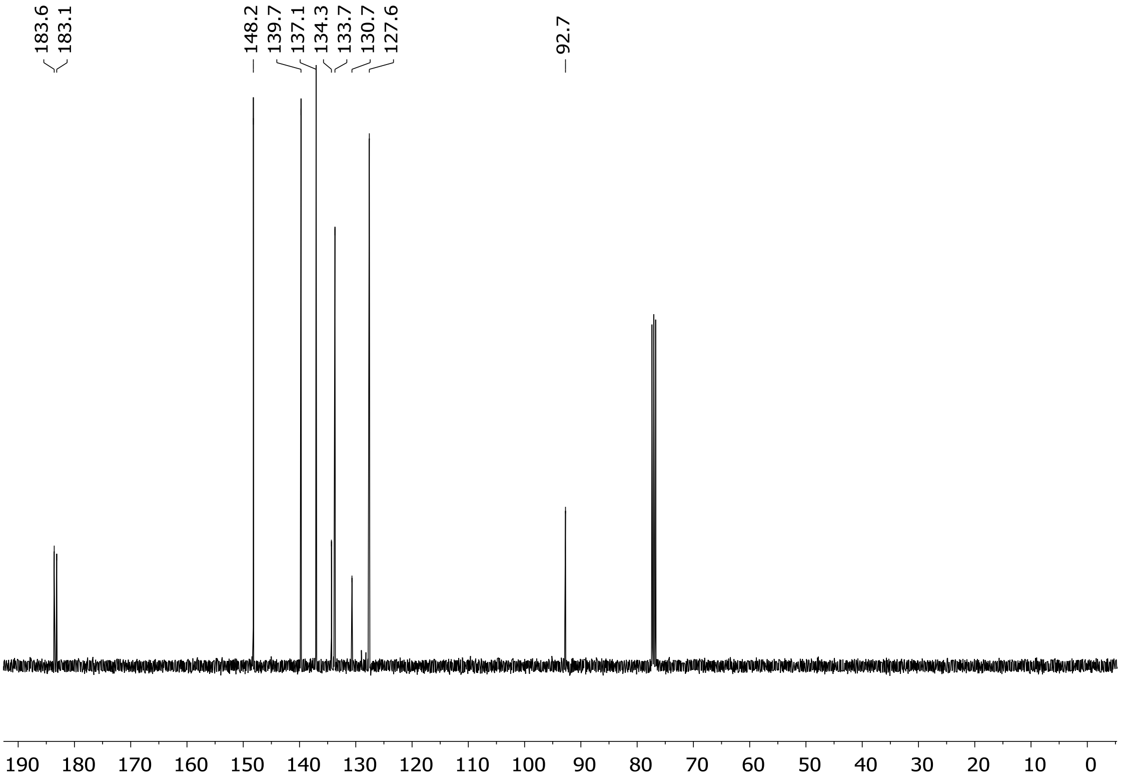
183.6
183.1
148.2
139.7
137.1
134.3
133.7
130.7
127.6
92.7
190 180 170 160 150 140 130 120 110 100 90 80 70 60 50 40 30 20 10 0

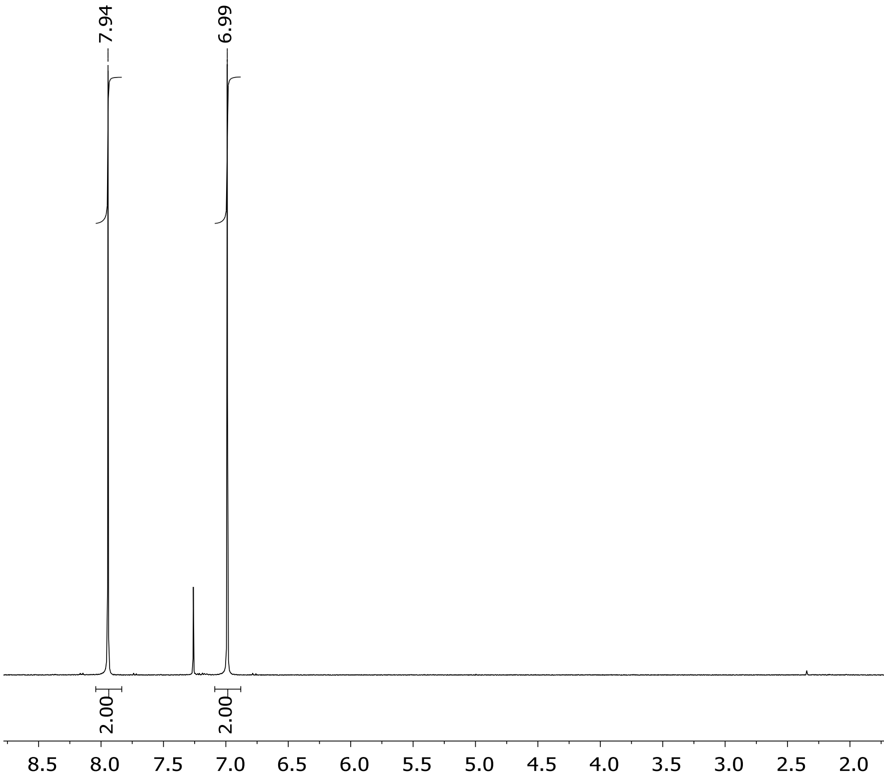

7.94
6.99
2.00
2.00
8.5 8.0 7.5 7.0 6.5 6.0 5.5 5.0 4.5 4.0 3.5 3.0 2.5 2.0 1.5 1.0 0.5 0.0

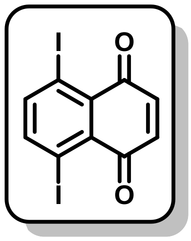

I
O
I
O

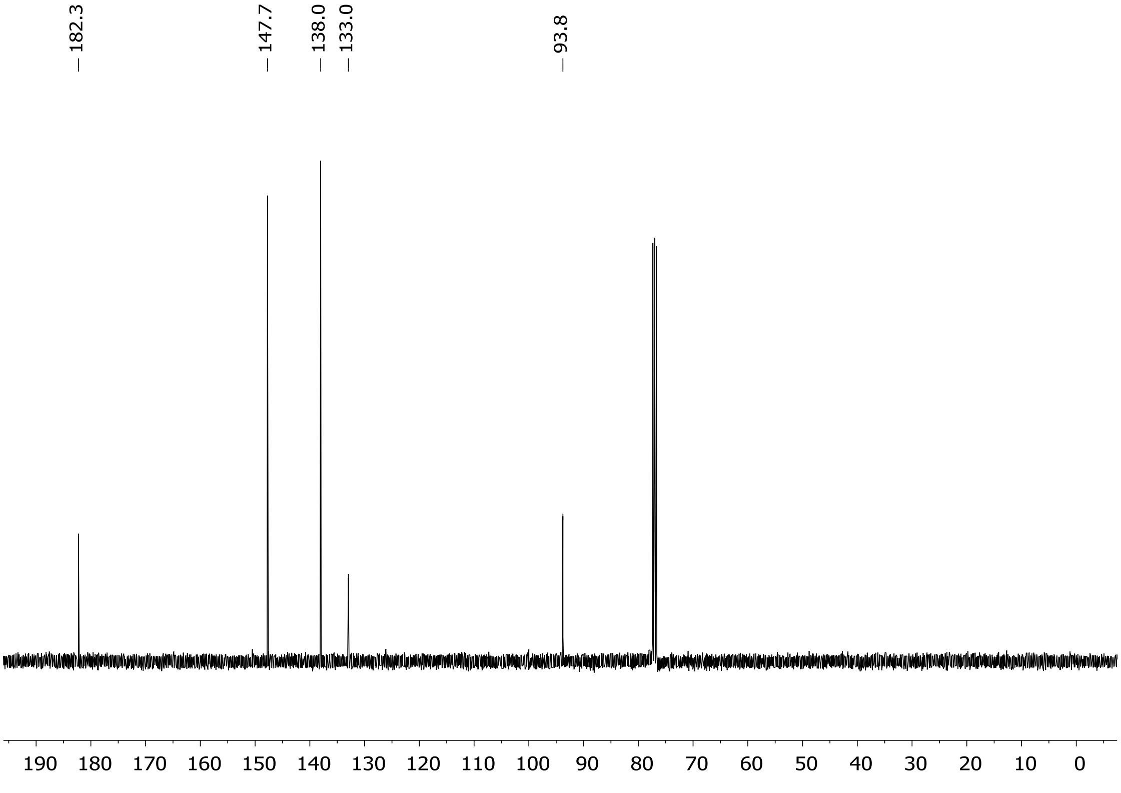

182.3
147.7
138.0
133.0
93.8
190 180 170 160 150 140 130 120 110 100 90 80 70 60 50 40 30 20 10 0

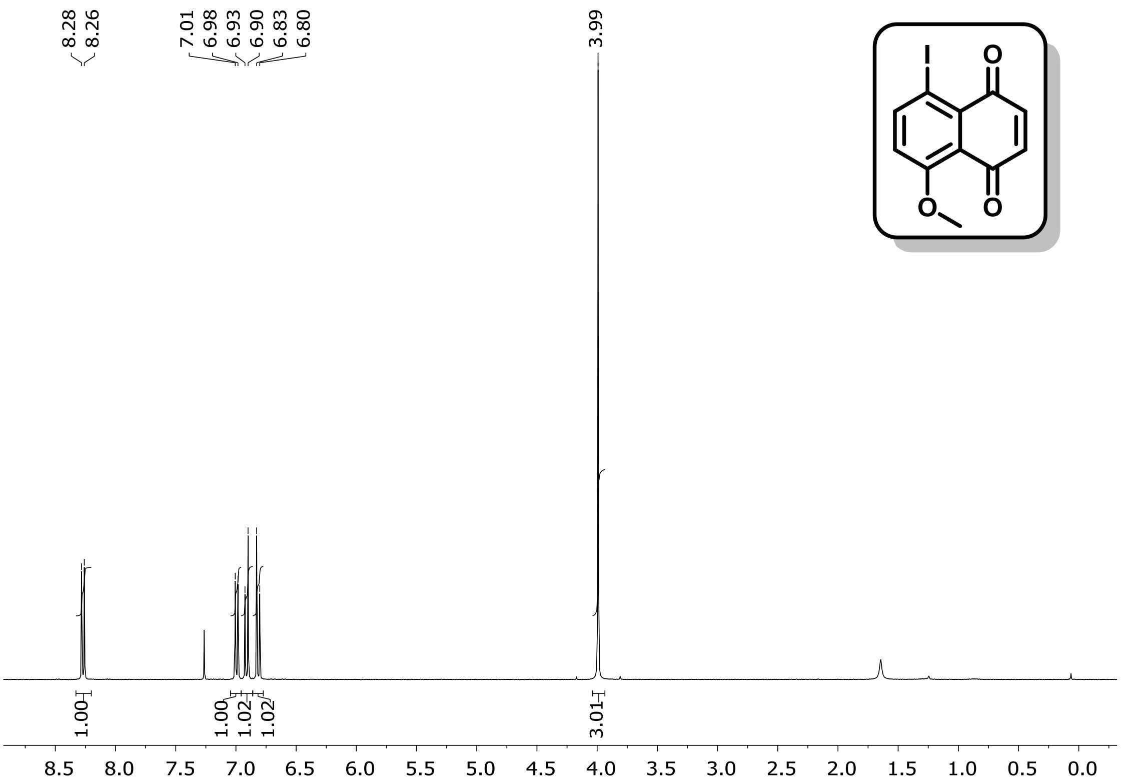
8.28
8.26
7.01
6.98
6.93
6.90
6.83
6.80
3.99
I
O
O
O
1.00
1.00
1.02
1.02
3.01
8.5 8.0 7.5 7.0 6.5 6.0 5.5 5.0 4.5 4.0 3.5 3.0 2.5 2.0 1.5 1.0 0.5 0.0

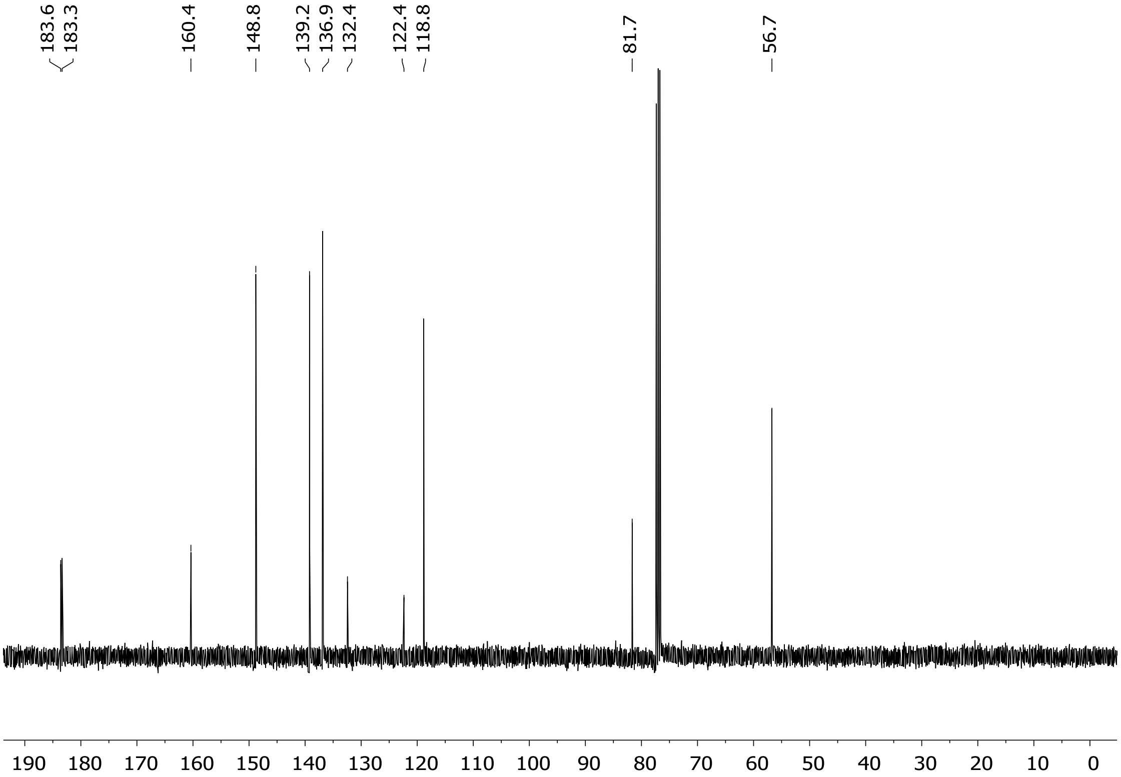
183.6
183.3
160.4
148.8
139.2
136.9
132.4
122.4
118.8
81.7
56.7
190 180 170 160 150 140 130 120 110 100 90 80 70 60 50 40 30 20 10 0

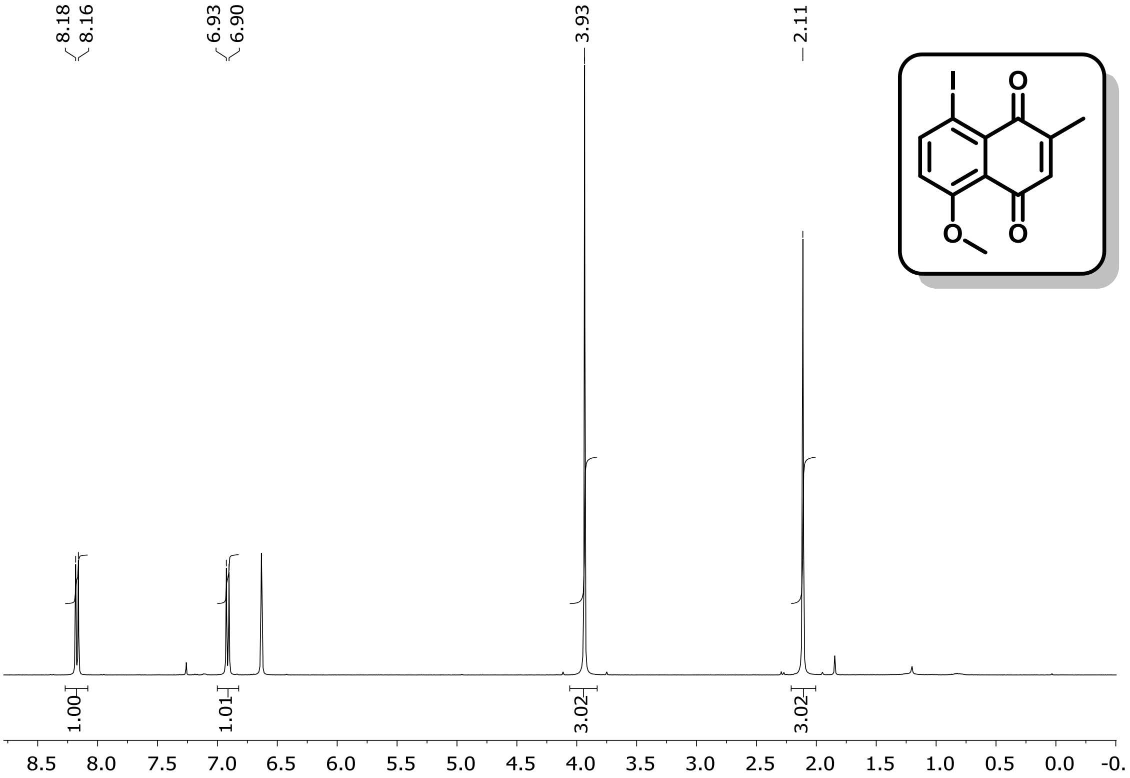
8.18
8.16
6.93
6.90
3.93
2.11
1.00
1.01
3.02
3.02
8.5 8.0 7.5 7.0 6.5 6.0 5.5 5.0 4.5 4.0 3.5 3.0 2.5 2.0 1.5 1.0 0.5 0.0 -0.

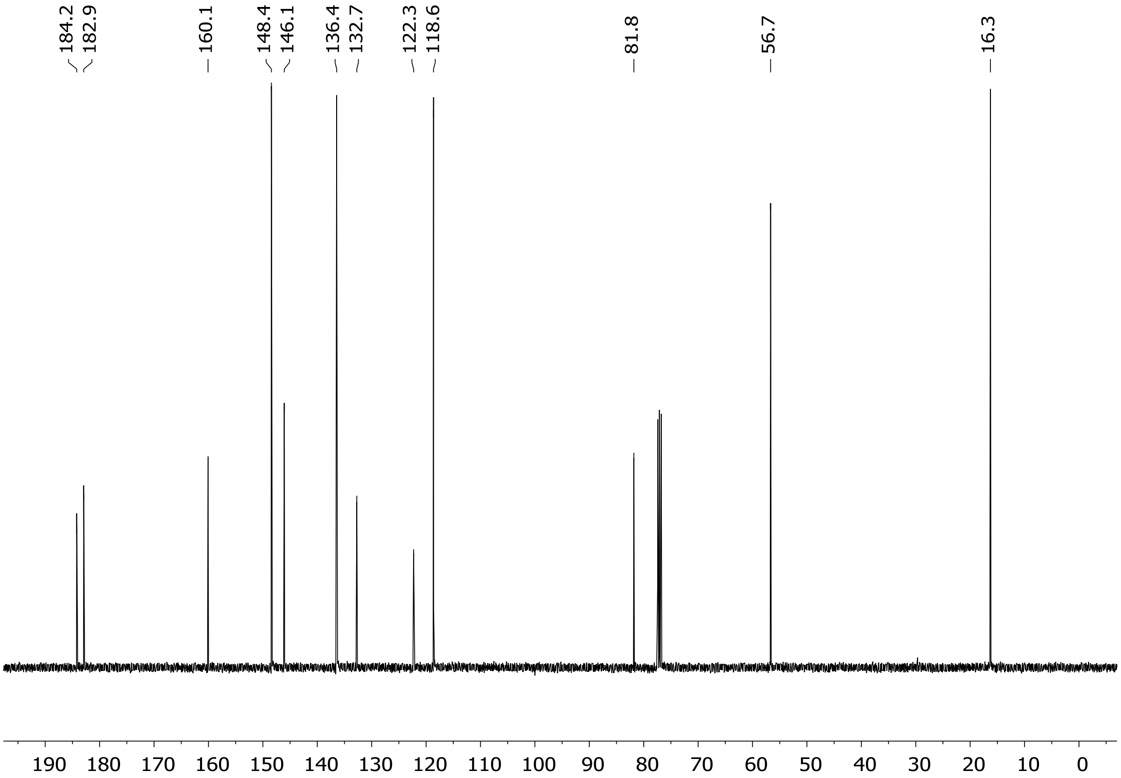
184.2
182.9
160.1
148.4
146.1
136.4
132.7
122.3
118.6
81.8
56.7
16.3
190 180 170 160 150 140 130 120 110 100 90 80 70 60 50 40 30 20 10 0

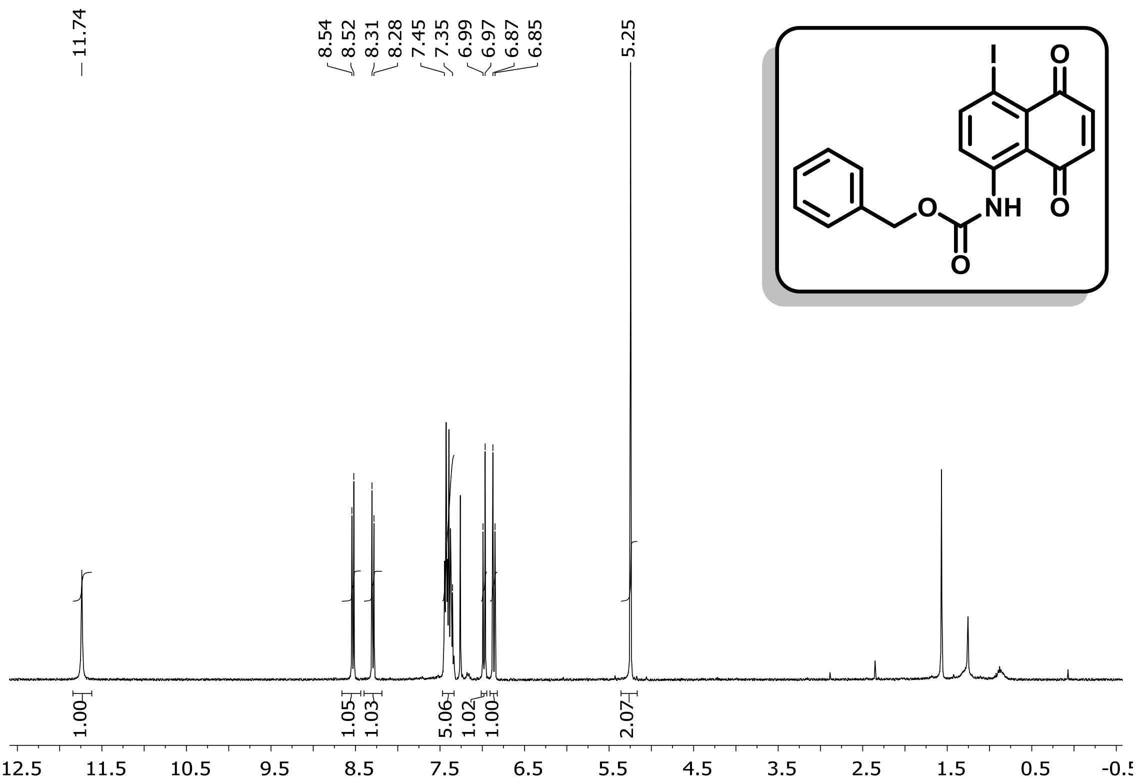
11.74
8.54
8.52
8.31
8.28
7.45
7.35
6.99
6.97
6.87
6.85
5.25
I
O
O
O
NH
O
1.00
1.05
1.03
5.06
1.02
1.00
2.07
12.5 11.5 10.5 9.5 8.5 7.5 6.5 5.5 4.5 3.5 2.5 1.5 0.5

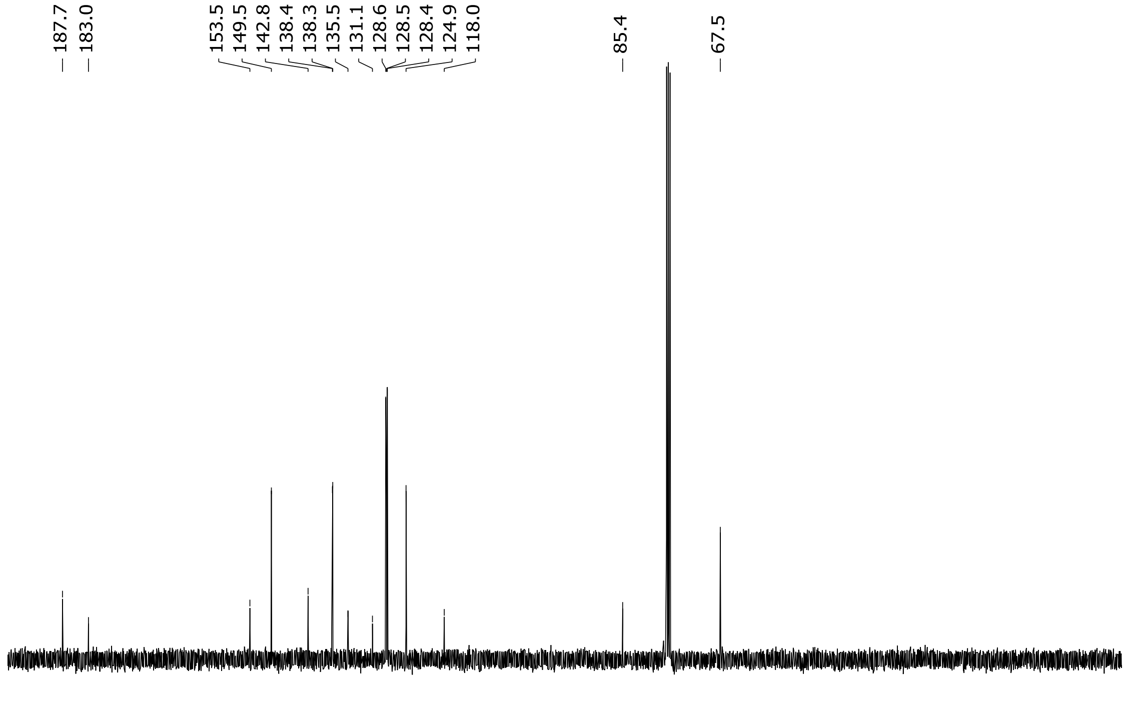
187.7
183.0
153.5
149.5
142.8
138.4
138.3
135.5
131.1
128.6
128.5
128.4
124.9
118.0
85.4
67.5
190 180 170 160 150 140 130 120 110 100 90 80 70 60 50 40 30 20 10 0

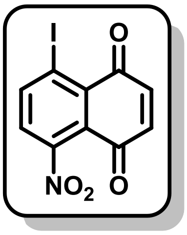
I
O
NO2
O

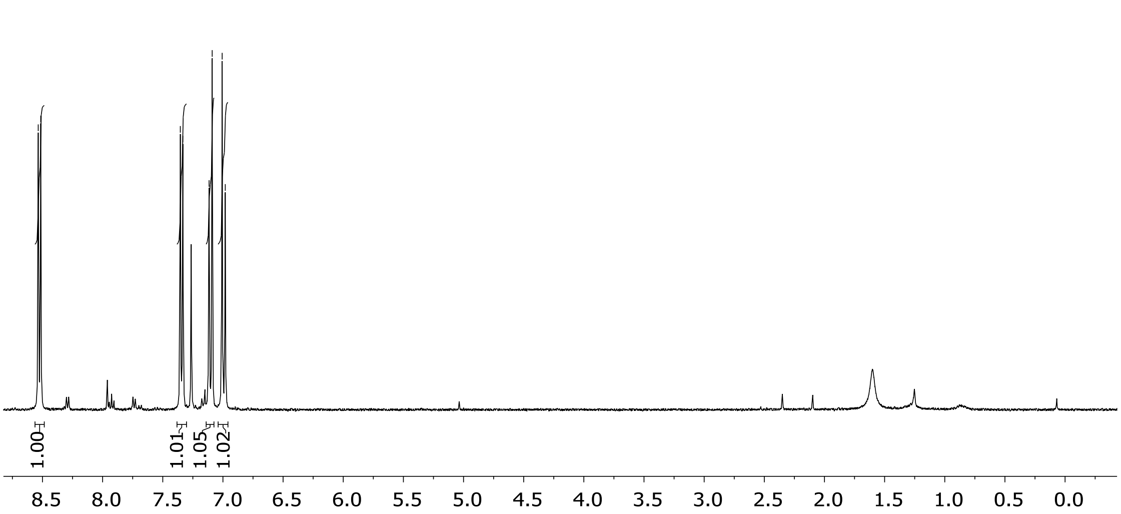
8.54
8.51
7.35
7.33
7.12
7.09
7.01
6.98
1.00
1.01
1.05
1.02
8.5 8.0 7.5 7.0 6.5 6.0 5.5 5.0 4.5 4.0 3.5 3.0 2.5 2.0 1.5 1.0 0.5 0.0

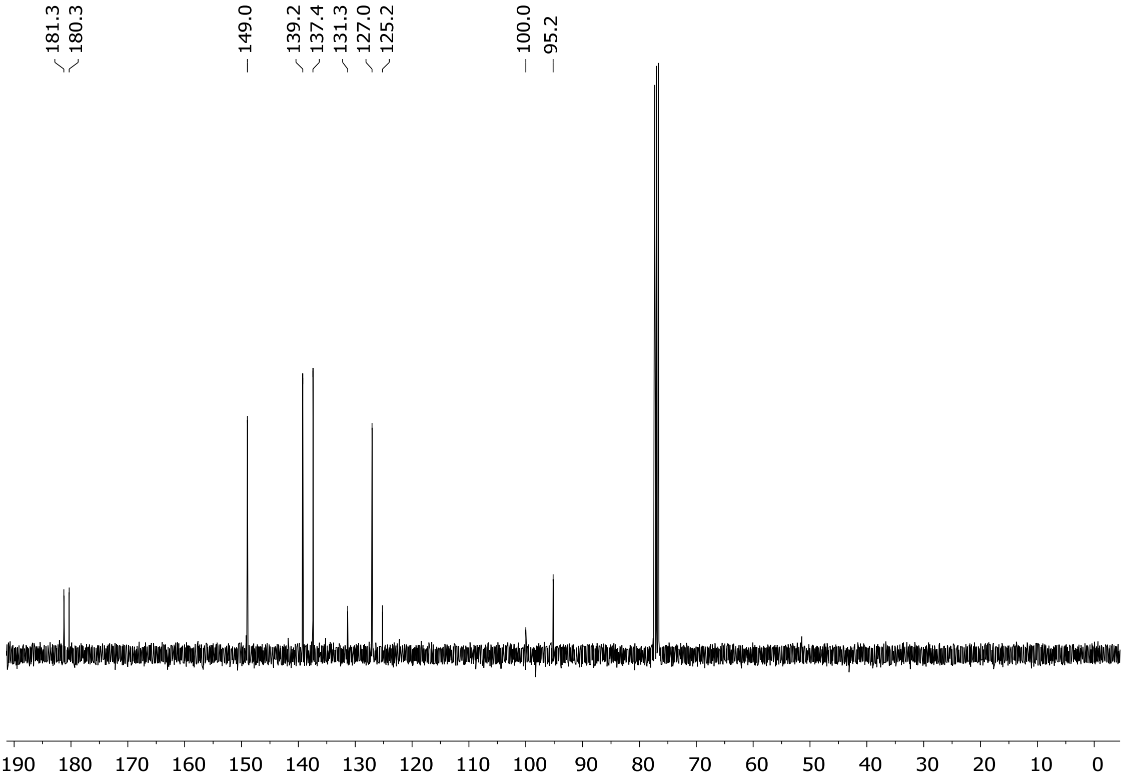
181.3
180.3
149.0
139.2
137.4
131.3
127.0
125.2
100.0
95.2
190 180 170 160 150 140 130 120 110 100 90 80 70 60 50 40 30 20 10 0

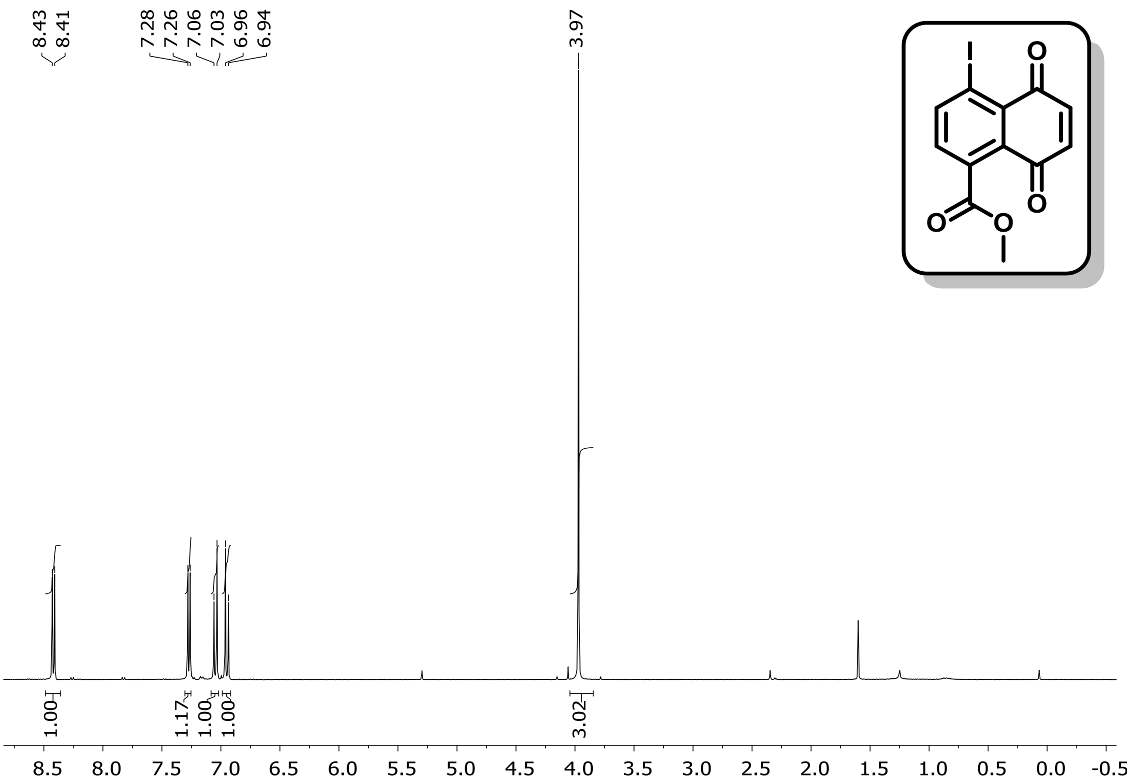
8.43
8.41
7.28
7.26
7.06
7.03
6.96
6.94
3.97
1.00
1.17
1.00
1.00
3.02
8.5 8.0 7.5 7.0 6.5 6.0 5.5 5.0 4.5 4.0 3.5 3.0 2.5 2.0 1.5 1.0 0.5 0.0 -0.5

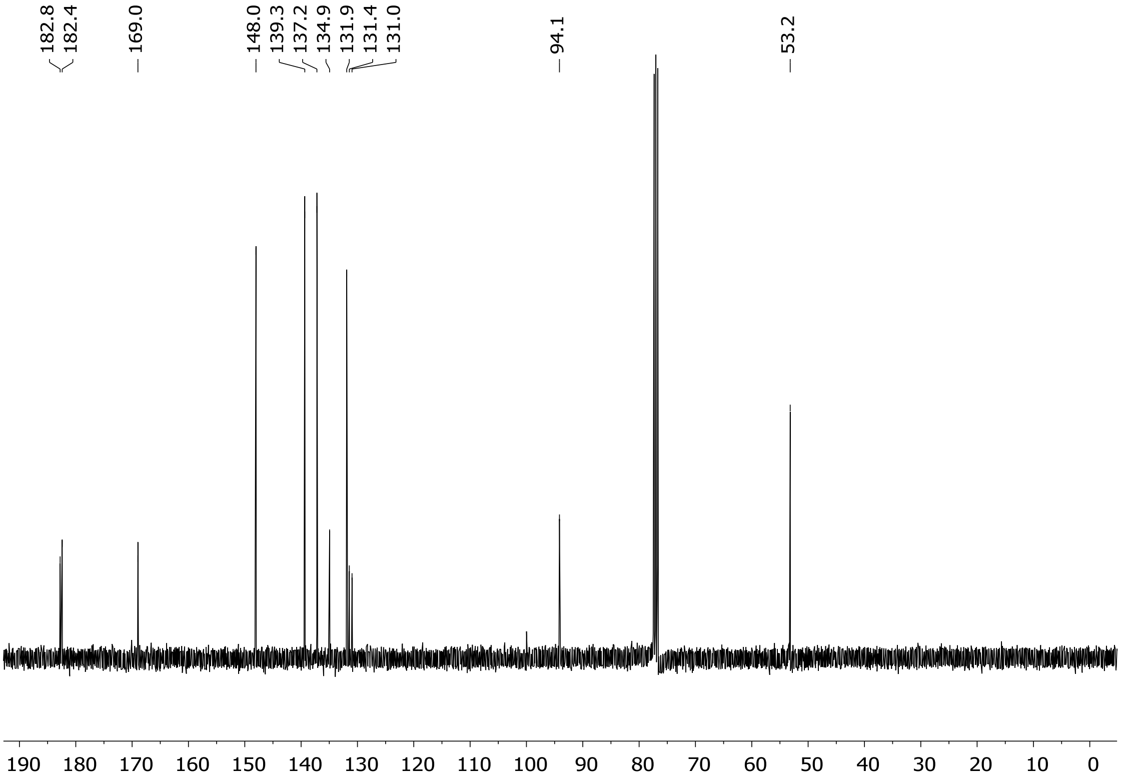
182.8
182.4
169.0
148.0
139.3
137.2
134.9
131.9
131.4
131.0
94.1
53.2
190 180 170 160 150 140 130 120 110 100 90 80 70 60 50 40 30 20 10 0

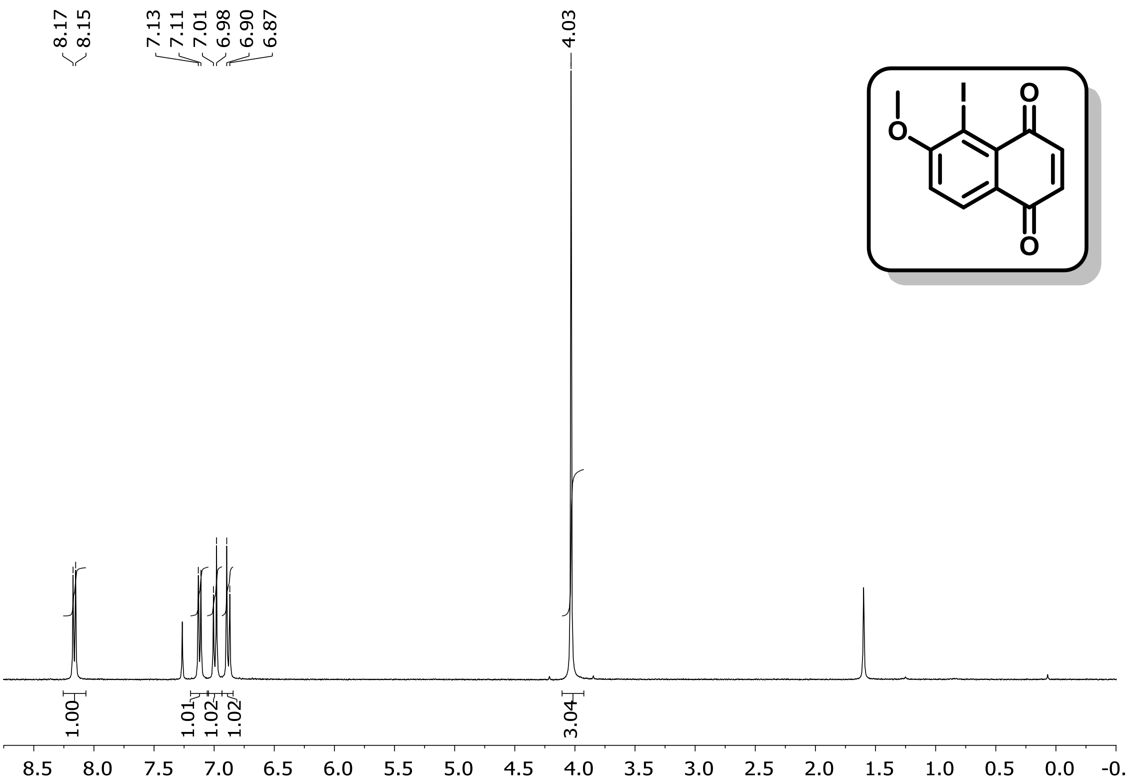
8.17
8.15
7.13
7.11
7.01
6.98
6.90
6.87
4.03
I
O
O
O
1.00
1.01
1.02
1.02
3.04
8.5 8.0 7.5 7.0 6.5 6.0 5.5 5.0 4.5 4.0 3.5 3.0 2.5 2.0 1.5 1.0 0.5 0.0 -0.

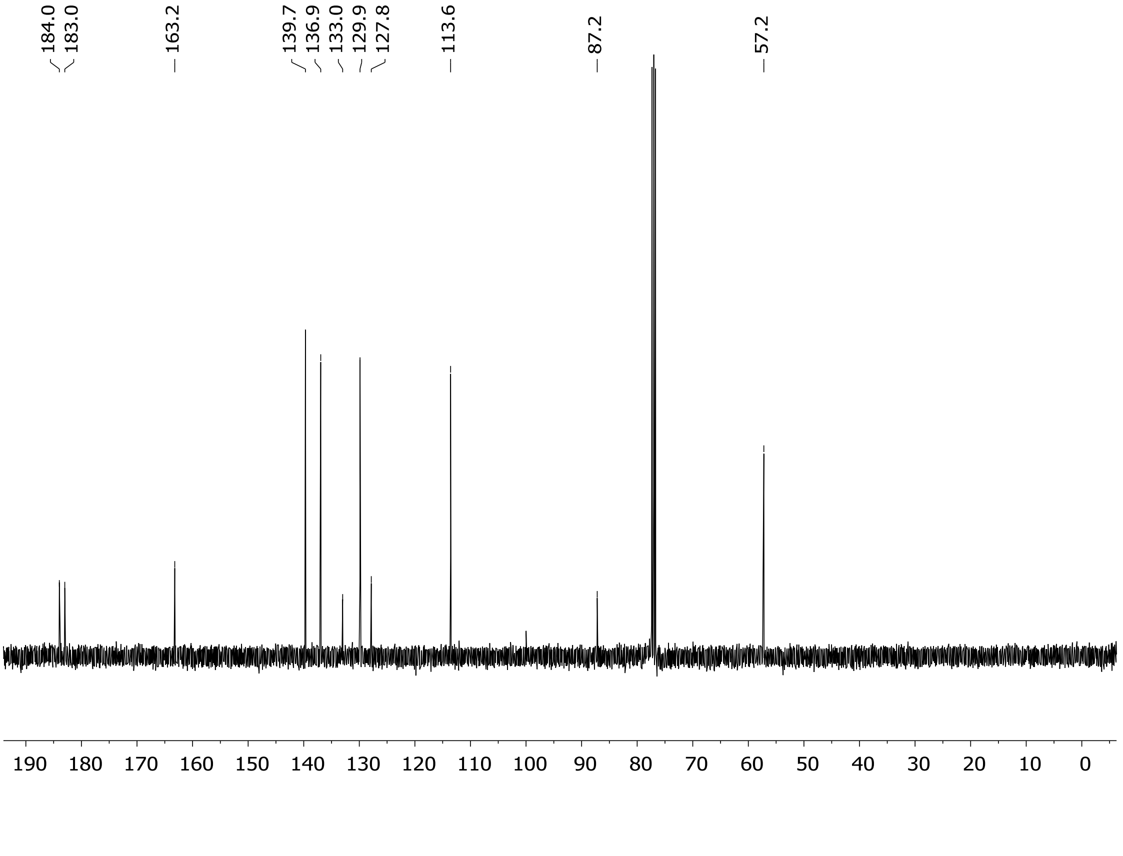
184.0
183.0
163.2
139.7
136.9
133.0
129.9
127.8
113.6
87.2
57.2
190 180 170 160 150 140 130 120 110 100 90 80 70 60 50 40 30 20 10 0

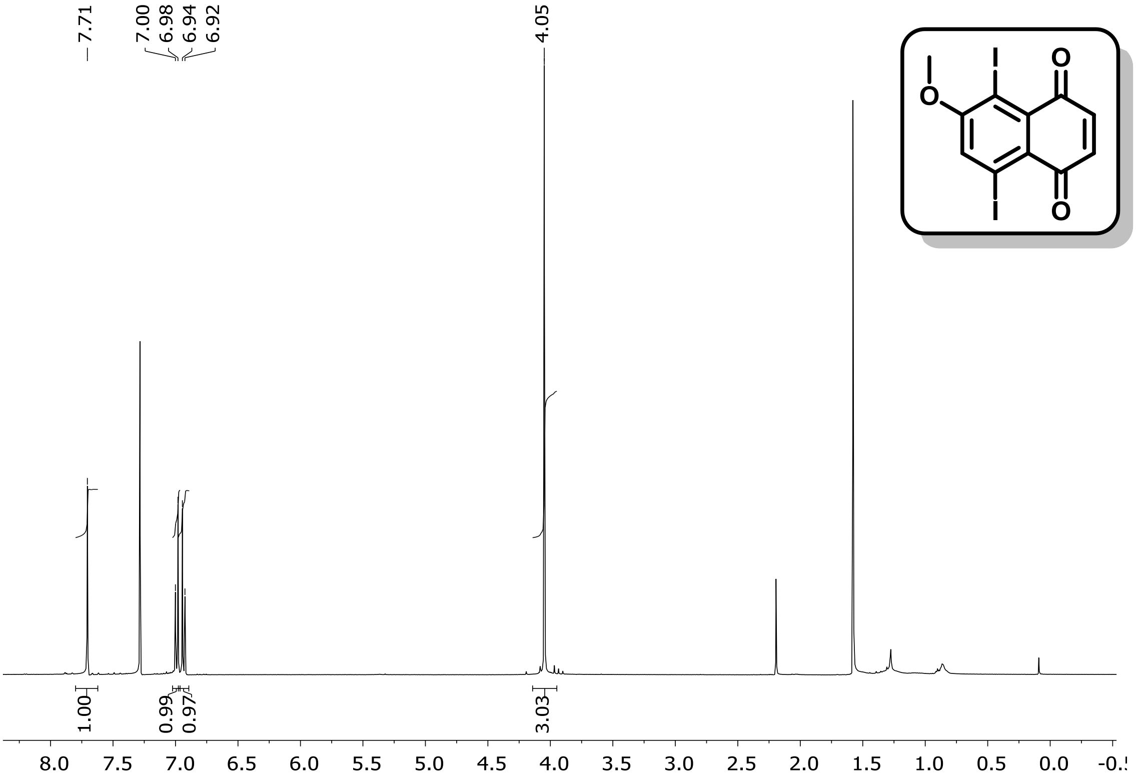
7.71
7.00
6.98
6.94
6.92
4.05
1.00
0.99
0.97
3.03
8.0 7.5 7.0 6.5 6.0 5.5 5.0 4.5 4.0 3.5 3.0 2.5 2.0 1.5 1.0 0.5 0.0
O
I
O
O
I

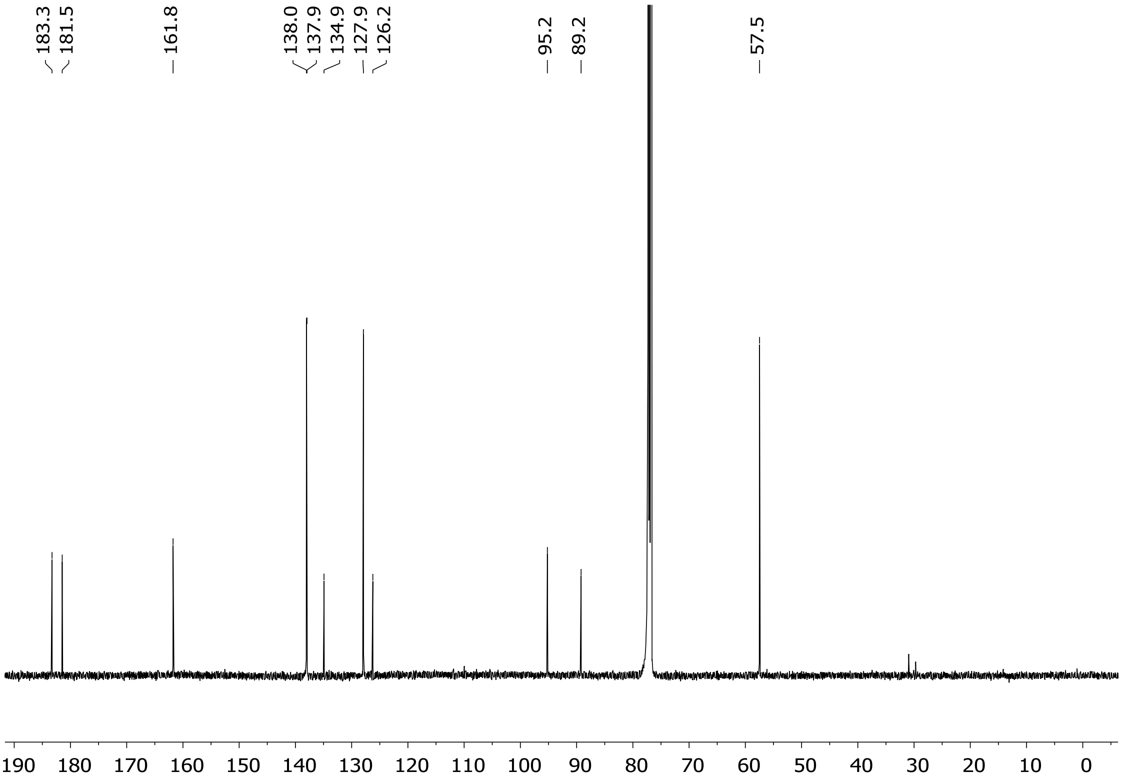
183.3
181.5
161.8
138.0
137.9
134.9
127.9
126.2
95.2
89.2
57.5
190 180 170 160 150 140 130 120 110 100 90 80 70 60 50 40 30 20 10 0

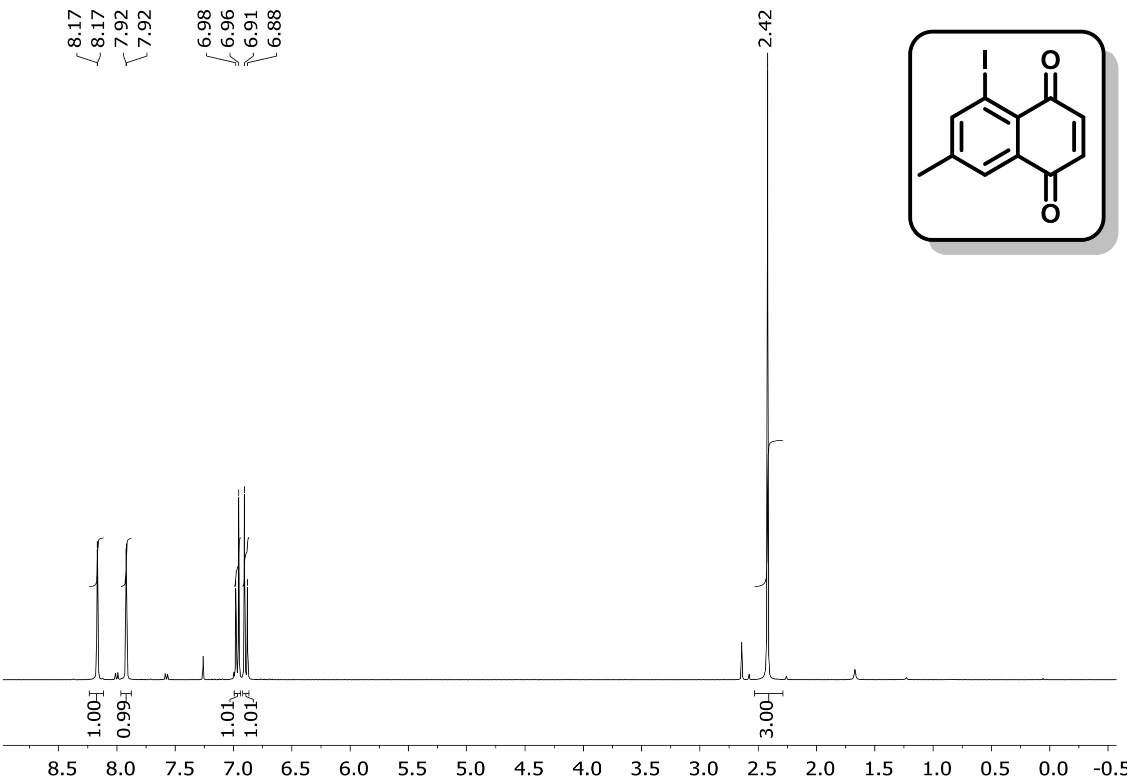
8.17
8.17
7.92
7.92
6.98
6.96
6.91
6.88
2.42
1.00
0.99
1.01
1.01
3.00
8.5 8.0 7.5 7.0 6.5 6.0 5.5 5.0 4.5 4.0 3.5 3.0 2.5 2.0 1.5 1.0 0.5 0.0 -0.5

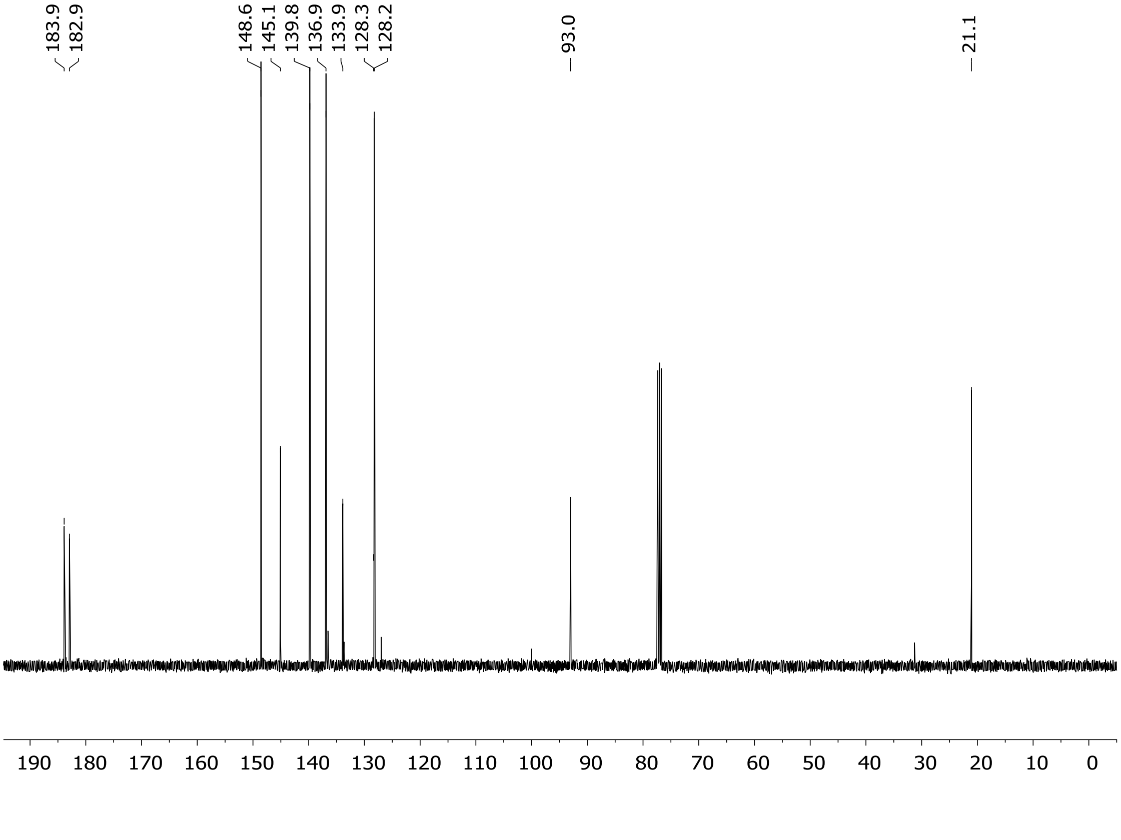
183.9
182.9
148.6
145.1
139.8
136.9
133.9
128.3
128.2
93.0
21.1
190 180 170 160 150 140 130 120 110 100 90 80 70 60 50 40 30 20 10 0

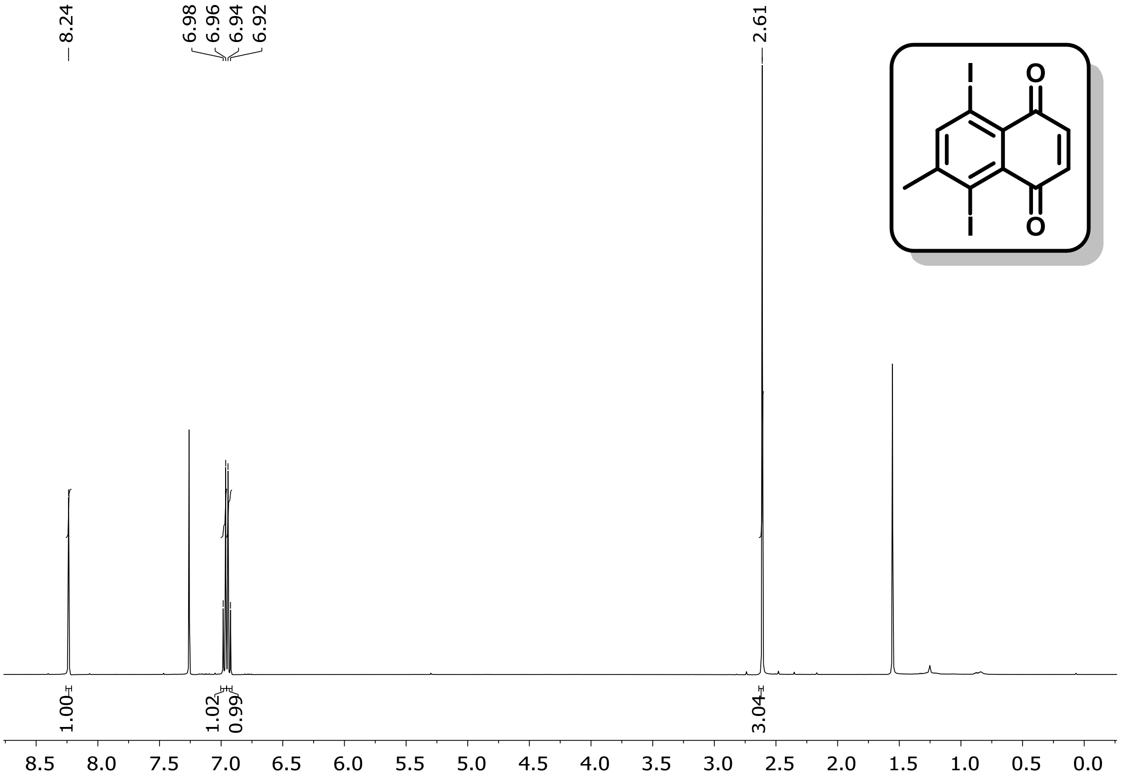
8.24
6.98
6.96
6.94
6.92
2.61
I
O
I
O
1.00
1.02
0.99
3.04
8.5 8.0 7.5 7.0 6.5 6.0 5.5 5.0 4.5 4.0 3.5 3.0 2.5 2.0 1.5 1.0 0.5 0.0

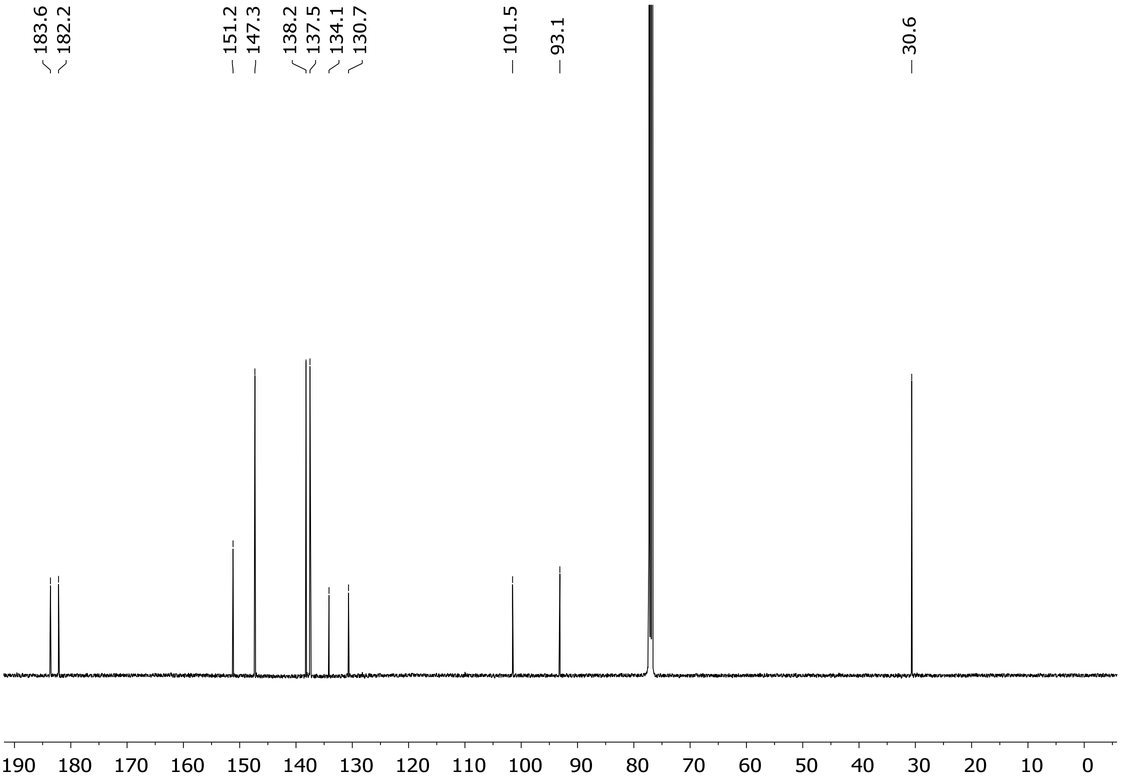
183.6
182.2
151.2
147.3
138.2
137.5
134.1
130.7
101.5
93.1
30.6
190 180 170 160 150 140 130 120 110 100 90 80 70 60 50 40 30 20 10 0

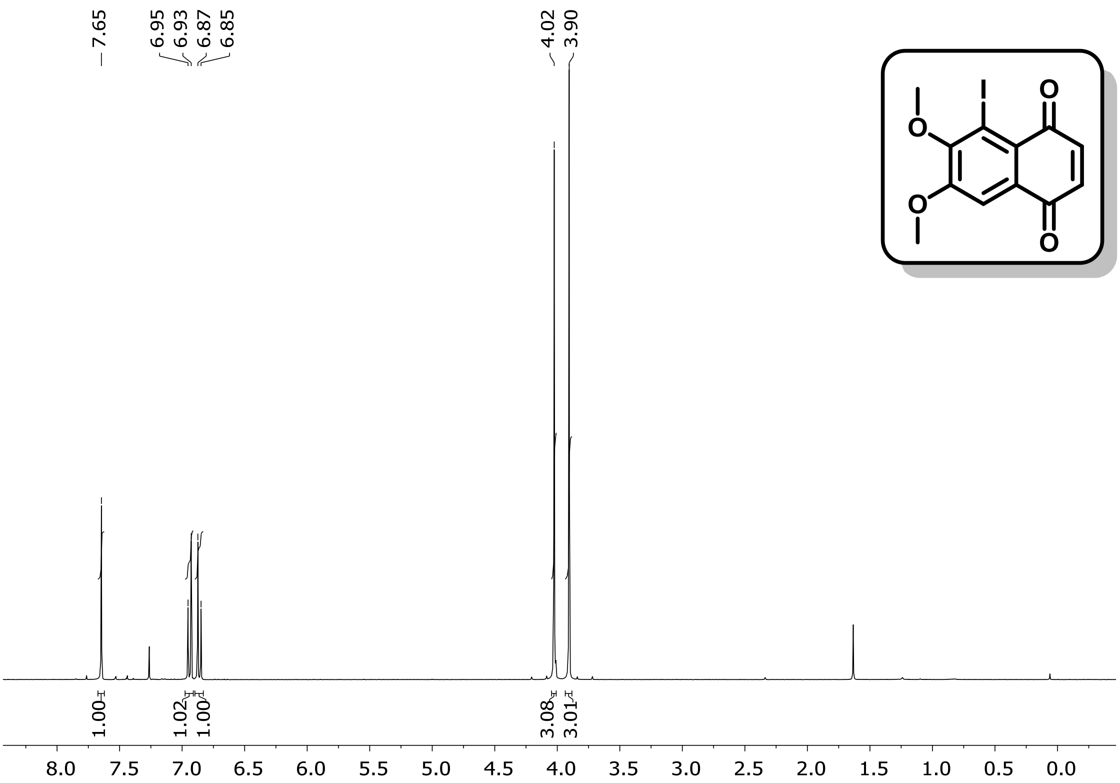
7.65
6.95
6.93
6.87
6.85
4.02
3.90
1.00
1.02
1.00
3.08
3.01
8.0 7.5 7.0 6.5 6.0 5.5 5.0 4.5 4.0 3.5 3.0 2.5 2.0 1.5 1.0 0.5 0.0
I
O
O
O
O

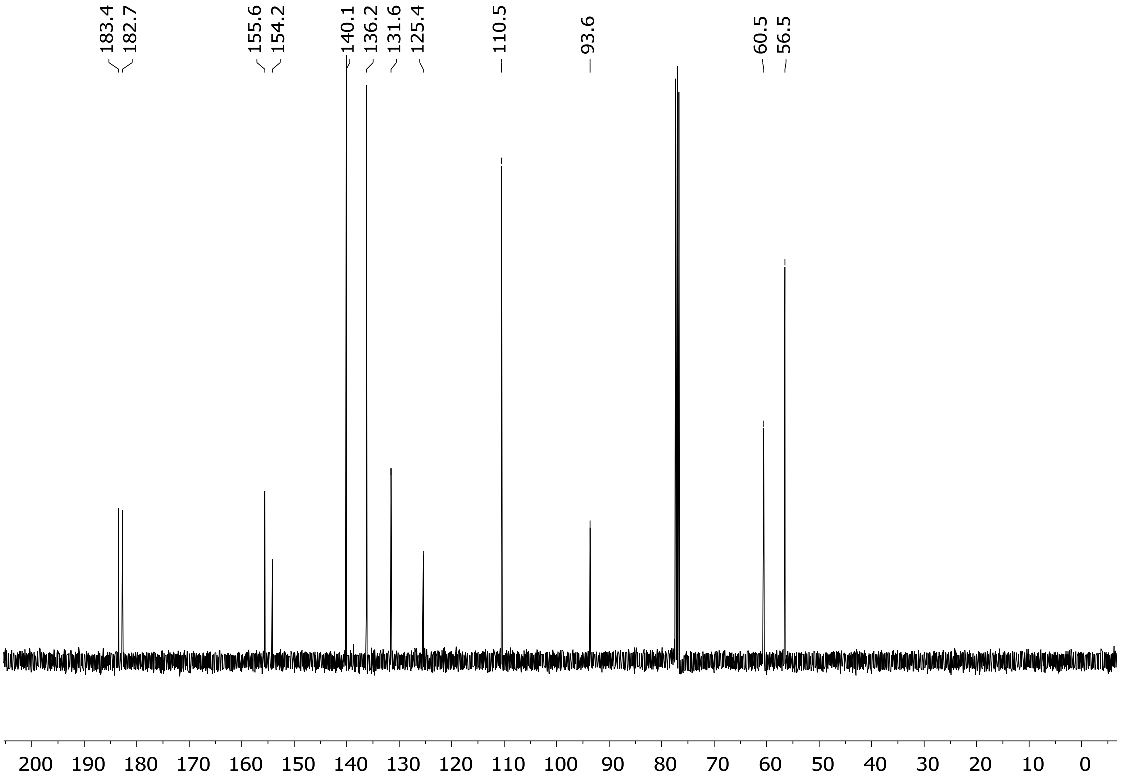
183.4
182.7
155.6
154.2
140.1
136.2
131.6
125.4
110.5
93.6
60.5
56.5
200 190 180 170 160 150 140 130 120 110 100 90 80 70 60 50 40 30 20 10 0

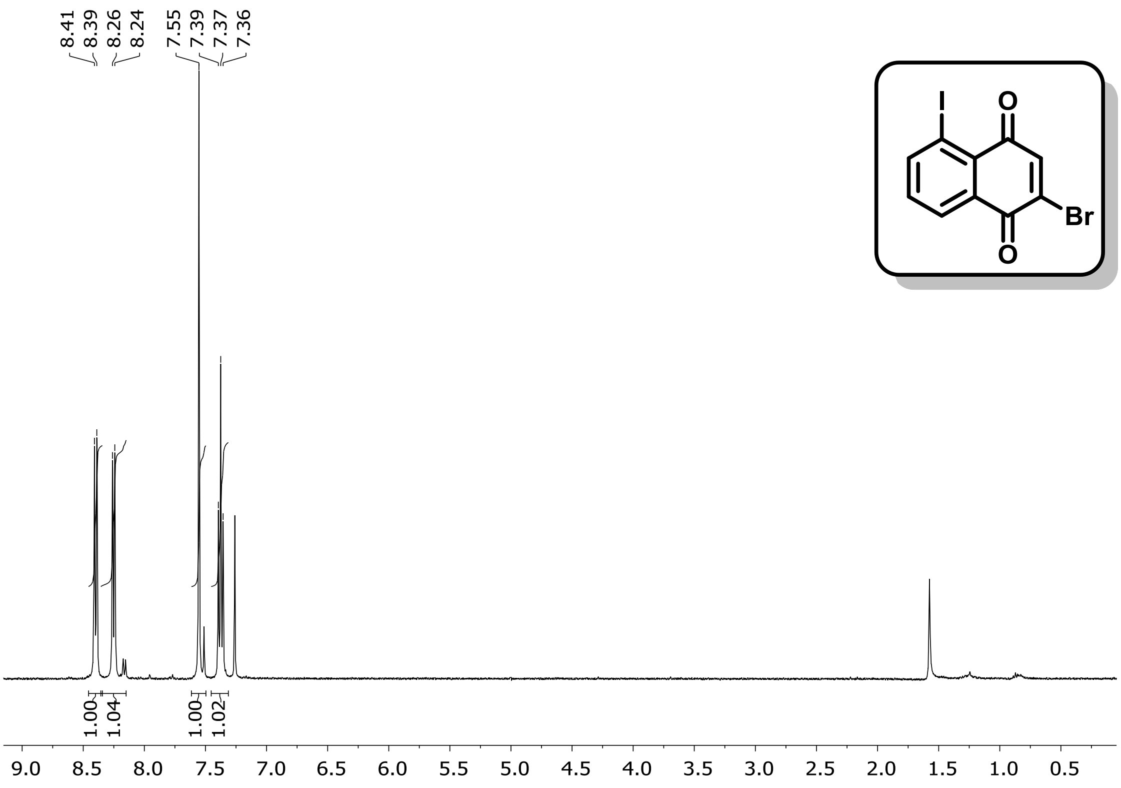

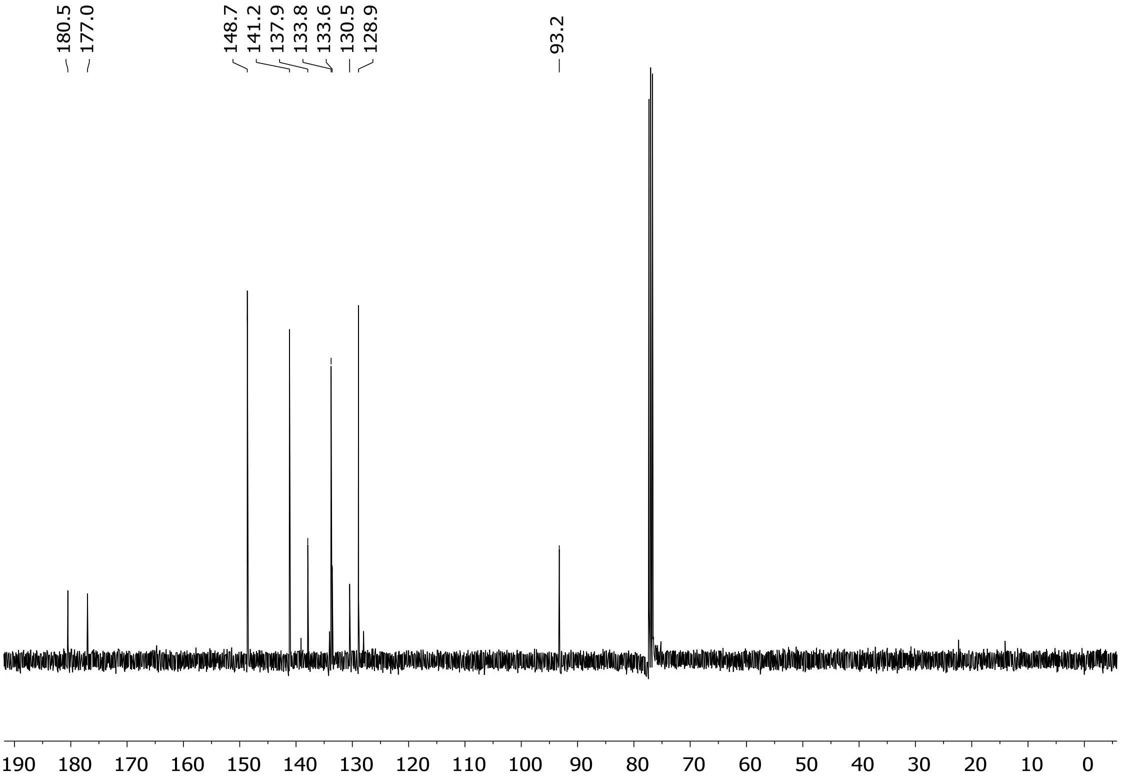

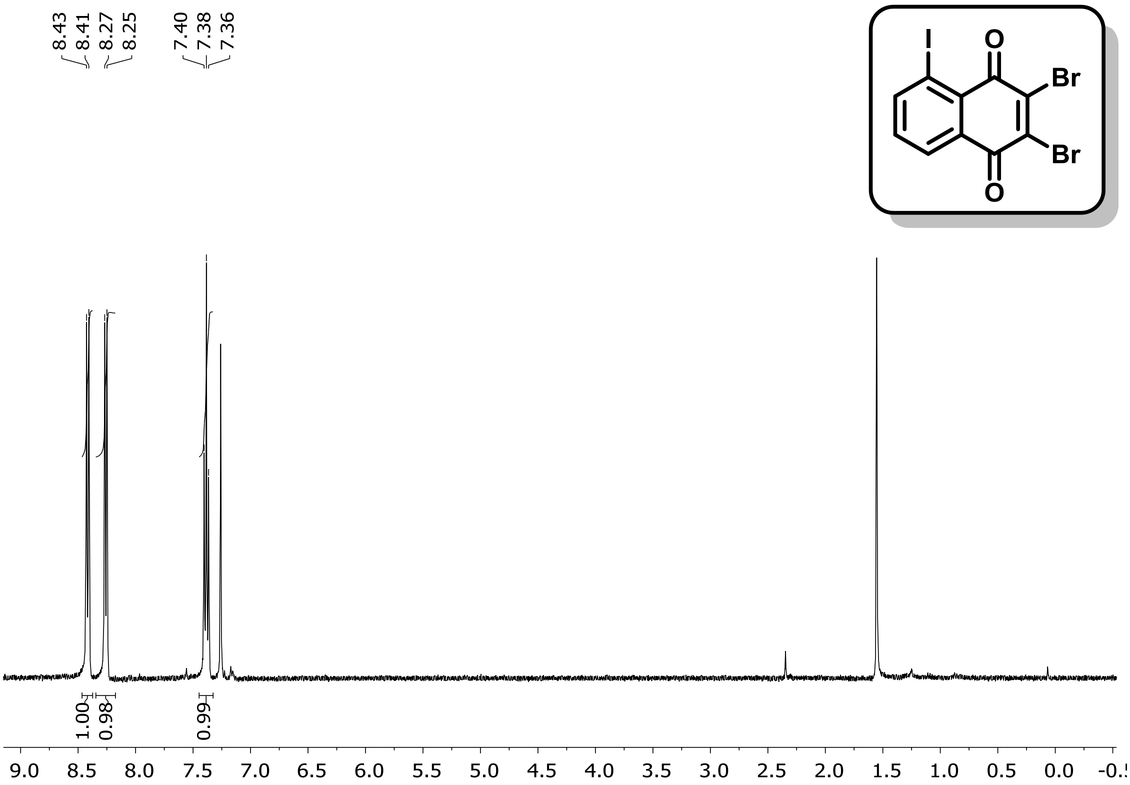
8.43
8.41
8.27
8.25
7.40
7.38
7.36
I
O
Br
Br
O
1.00
0.98
0.99
9.0 8.5 8.0 7.5 7.0 6.5 6.0 5.5 5.0 4.5 4.0 3.5 3.0 2.5 2.0 1.5 1.0 0.5 0.0

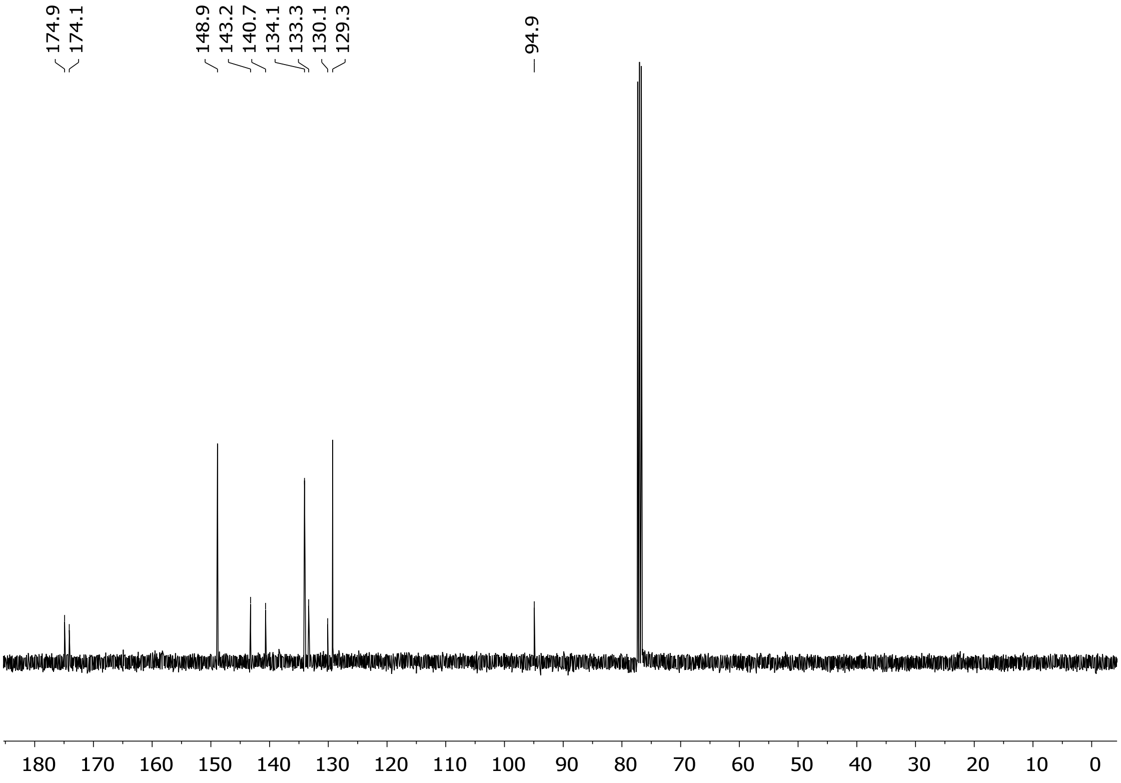
174.9
174.1
148.9
143.2
140.7
134.1
133.3
130.1
129.3
94.9
180 170 160 150 140 130 120 110 100 90 80 70 60 50 40 30 20 10 0

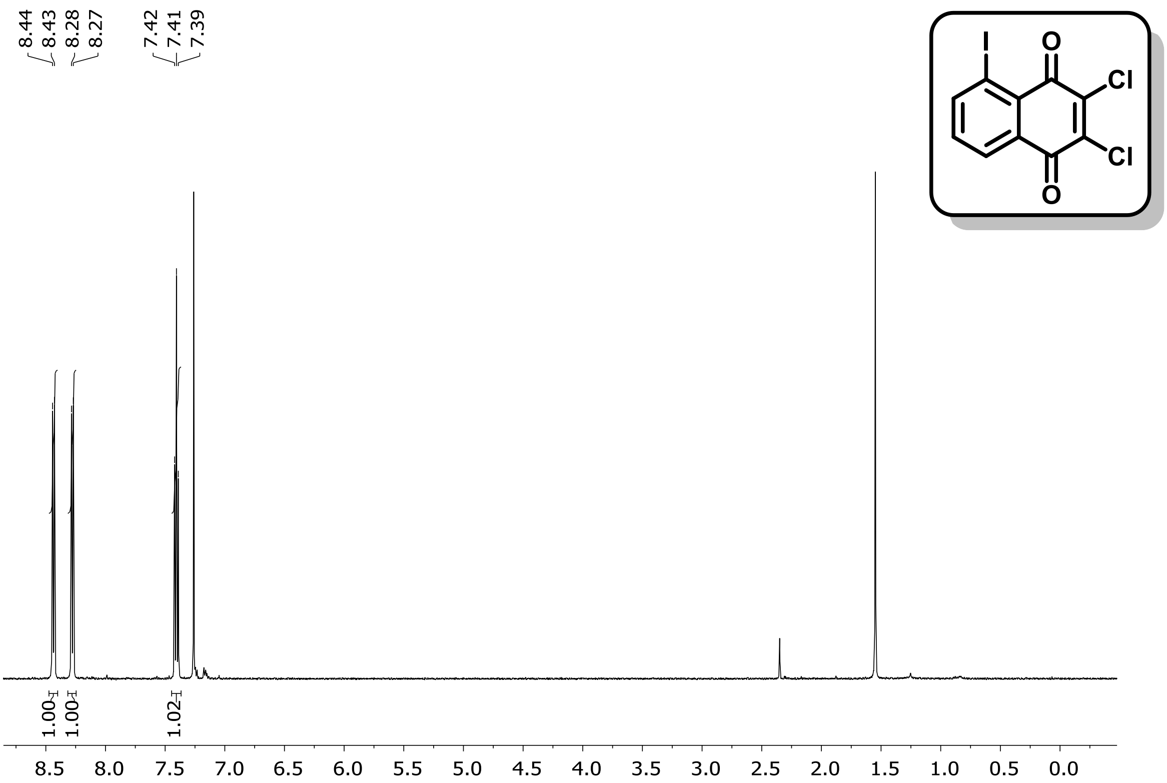
8.44
8.43
8.28
8.27
7.42
7.41
7.39
1.00
1.00
1.02
8.5 8.0 7.5 7.0 6.5 6.0 5.5 5.0 4.5 4.0 3.5 3.0 2.5 2.0 1.5 1.0 0.5 0.0
I
O
Cl
Cl
O

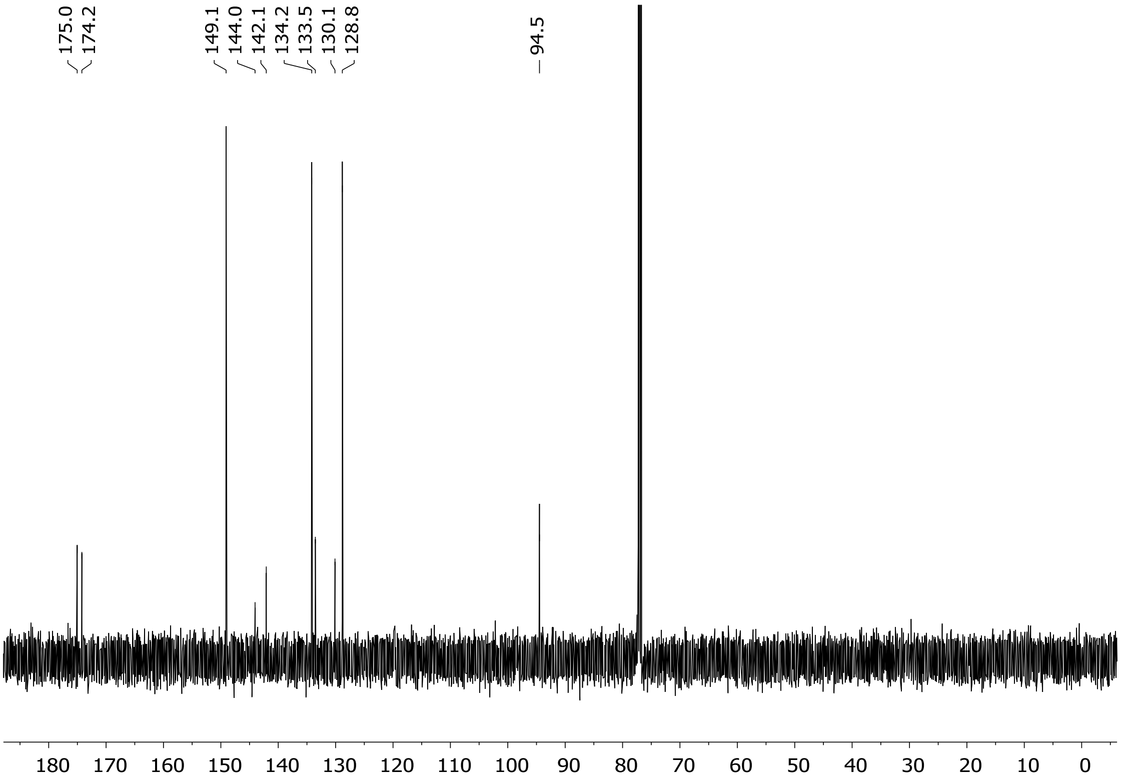
175.0
174.2
149.1
144.0
142.1
134.2
133.5
130.1
128.8
94.5
180 170 160 150 140 130 120 110 100 90 80 70 60 50 40 30 20 10 0

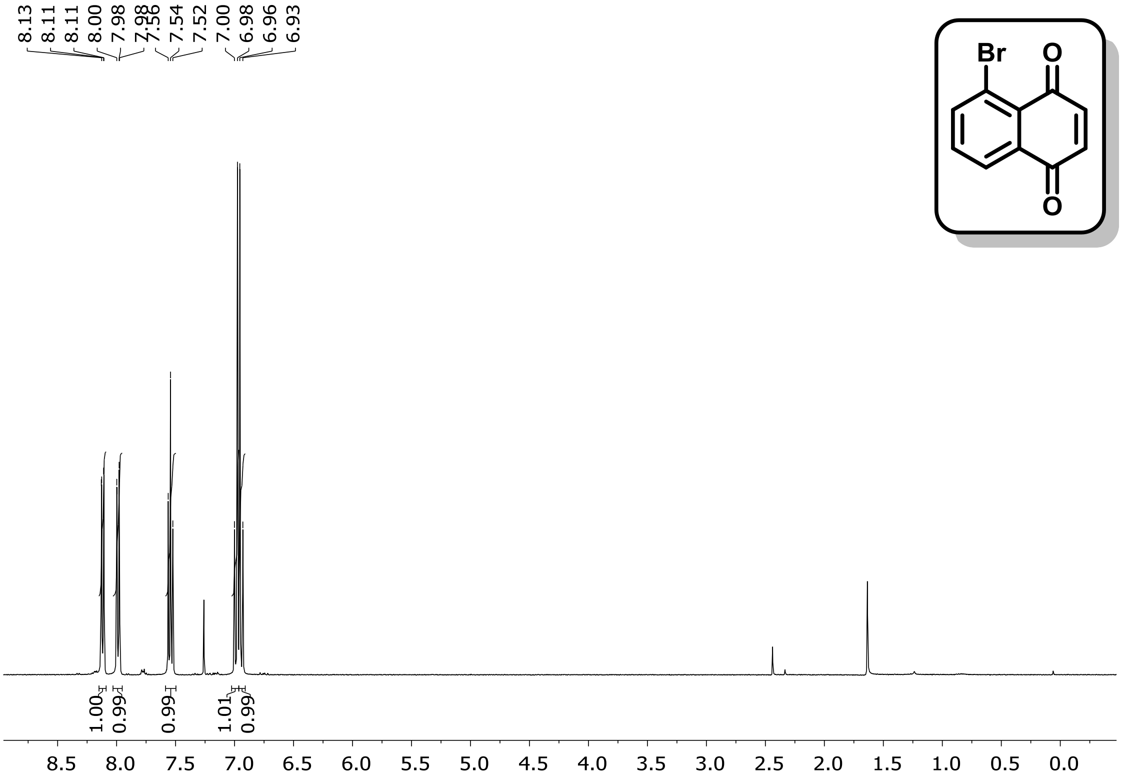
8.13
8.11
8.11
8.00
7.98
7.98
7.56
7.54
7.52
7.00
6.98
6.96
6.93
Br
O
O
1.00
0.99
0.99
1.01
0.99
8.5 8.0 7.5 7.0 6.5 6.0 5.5 5.0 4.5 4.0 3.5 3.0 2.5 2.0 1.5 1.0 0.5 0.0

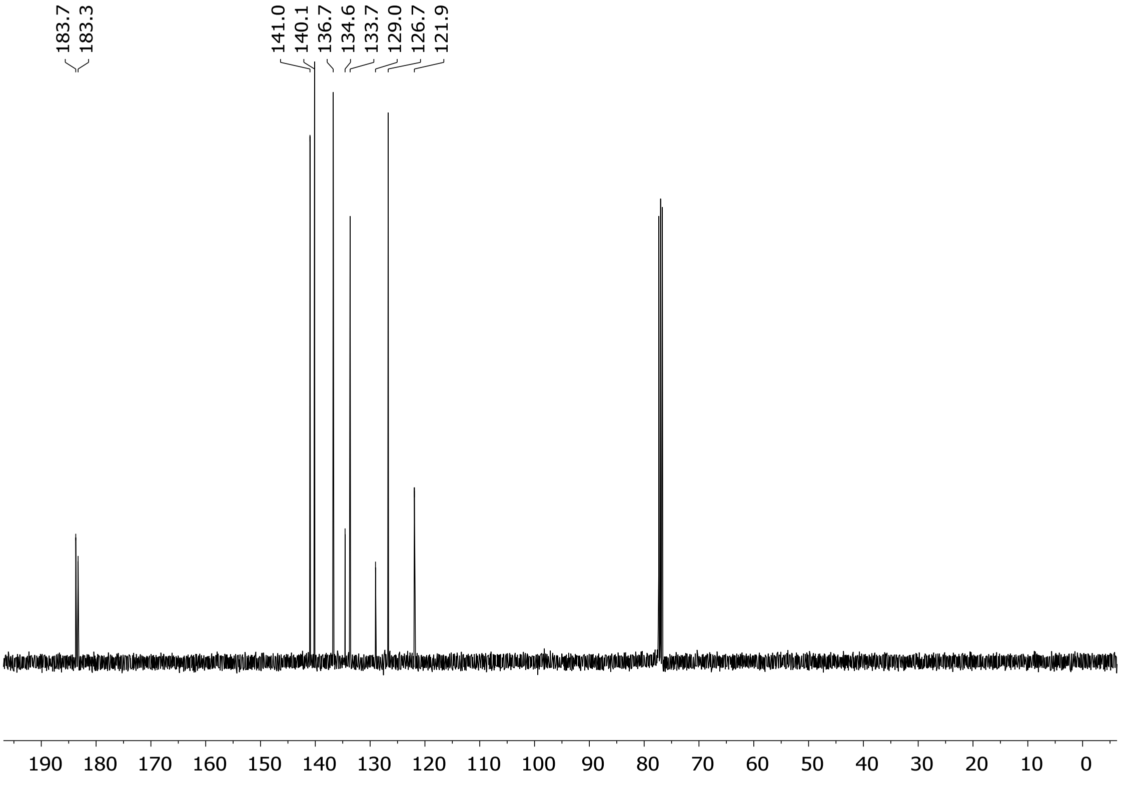
183.7
183.3
141.0
140.1
136.7
134.6
133.7
129.0
126.7
121.9
190 180 170 160 150 140 130 120 110 100 90 80 70 60 50 40 30 20 10 0

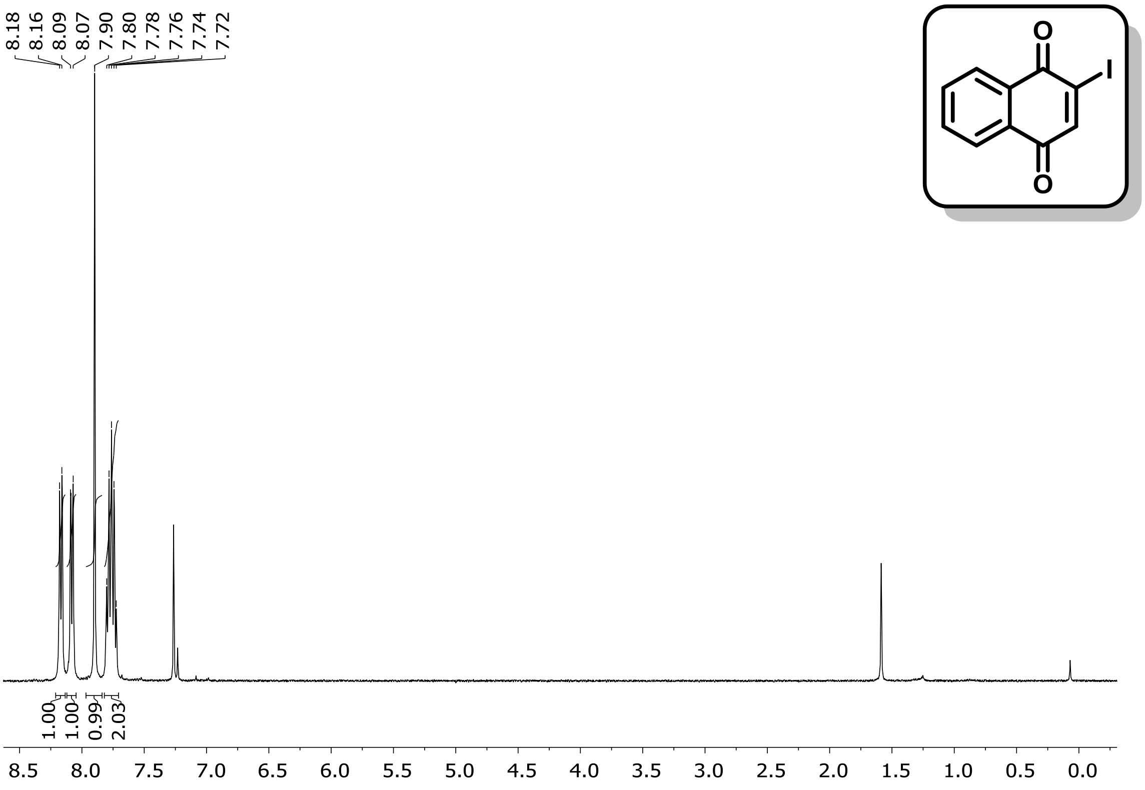

8.18
8.16
8.09
8.07
7.90
7.80
7.78
7.76
7.74
7.72
O
I
O
1.00
1.00
0.99
2.03
8.5 8.0 7.5 7.0 6.5 6.0 5.5 5.0 4.5 4.0 3.5 3.0 2.5 2.0 1.5 1.0 0.5 0.0

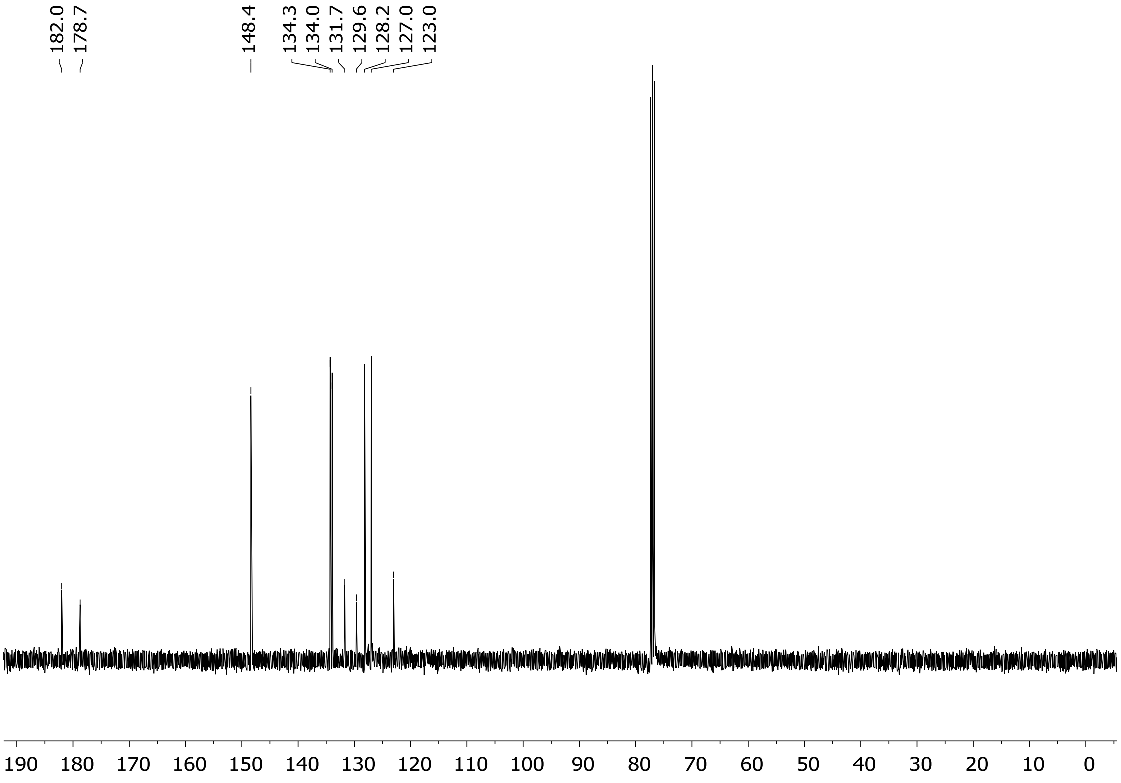

182.0
178.7
148.4
134.3
134.0
131.7
129.6
128.2
127.0
123.0
190 180 170 160 150 140 130 120 110 100 90 80 70 60 50 40 30 20 10 0

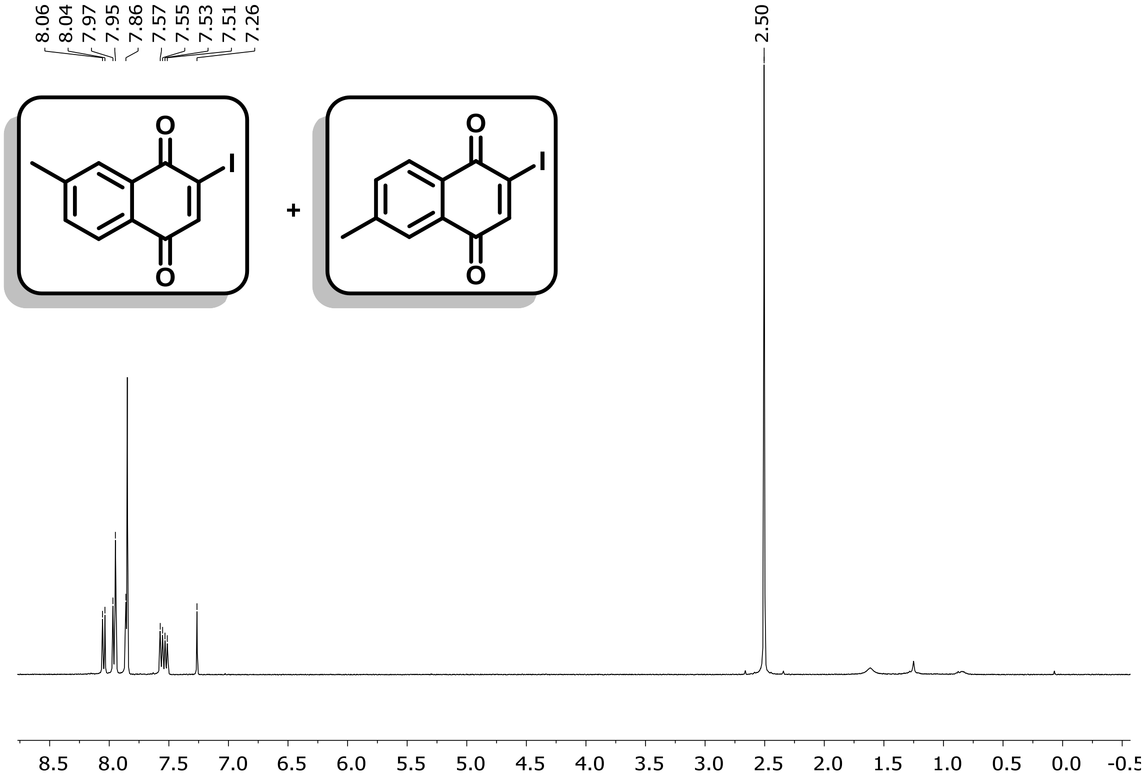
8.06
8.04
7.97
7.95
7.86
7.57
7.55
7.53
7.51
7.26
2.50
+
8.5 8.0 7.5 7.0 6.5 6.0 5.5 5.0 4.5 4.0 3.5 3.0 2.5 2.0 1.5 1.0 0.5 0.0

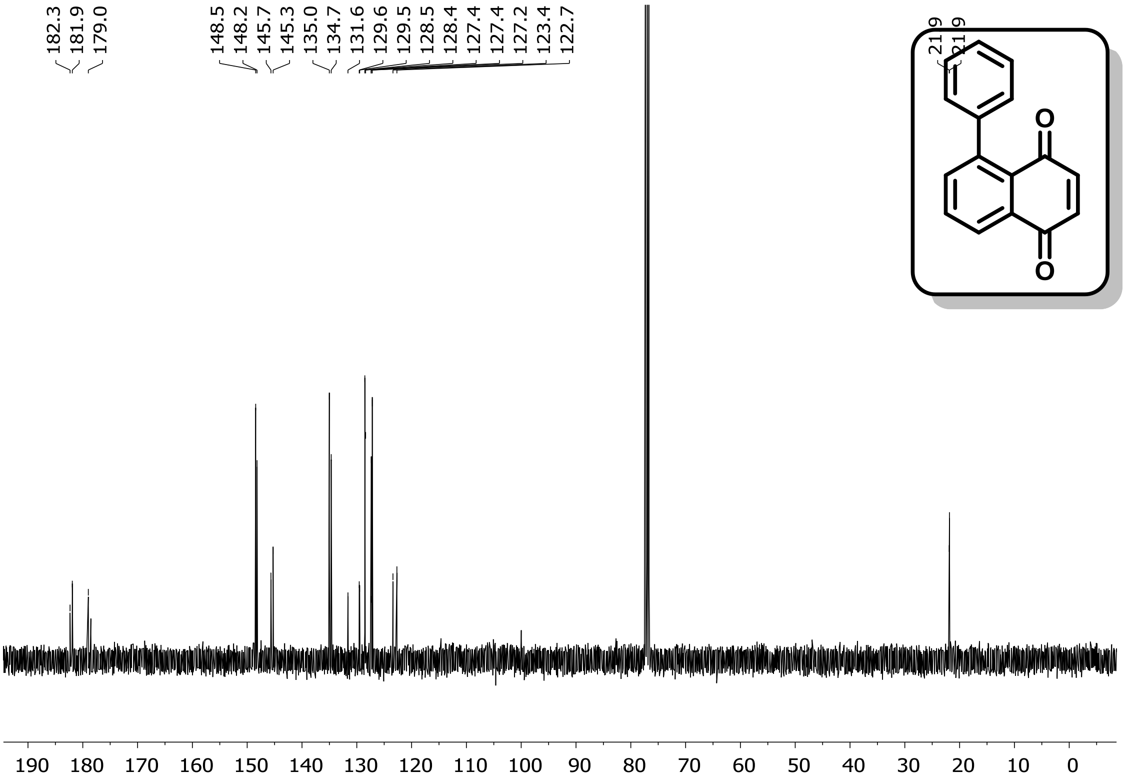
182.3
181.9
179.0
148.5
148.2
145.7
145.3
135.0
134.7
131.6
129.6
129.5
128.5
128.4
127.4
127.4
127.2
123.4
122.7
190 180 170 160 150 140 130 120 110 100 90 80 70 60 50 40 30 20 10 0

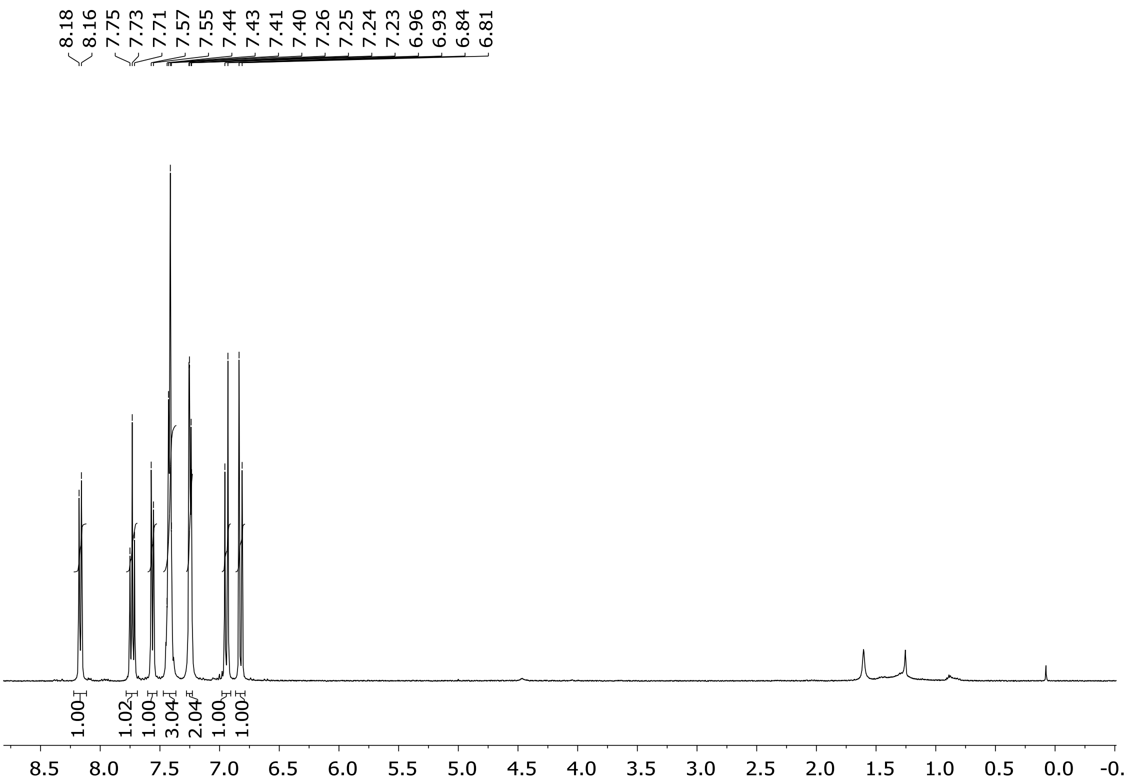
8.18
8.16
7.75
7.73
7.71
7.57
7.55
7.44
7.43
7.41
7.40
7.26
7.25
7.24
7.23
6.96
6.93
6.84
6.81
1.00
1.02
1.00
3.04
2.04
1.00
1.00
8.5 8.0 7.5 7.0 6.5 6.0 5.5 5.0 4.5 4.0 3.5 3.0 2.5 2.0 1.5 1.0 0.5 0.0 -0.

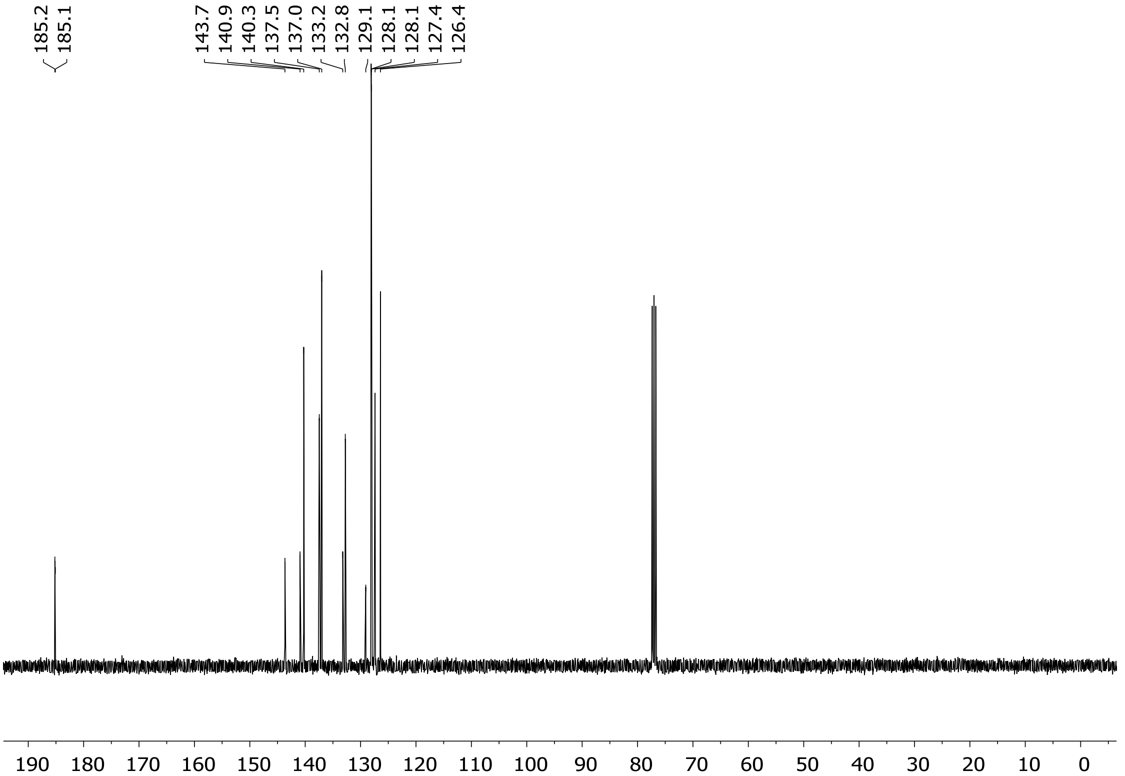
185.2
185.1
143.7
140.9
140.3
137.5
137.0
133.2
132.8
129.1
128.1
128.1
127.4
126.4
190 180 170 160 150 140 130 120 110 100 90 80 70 60 50 40 30 20 10 0

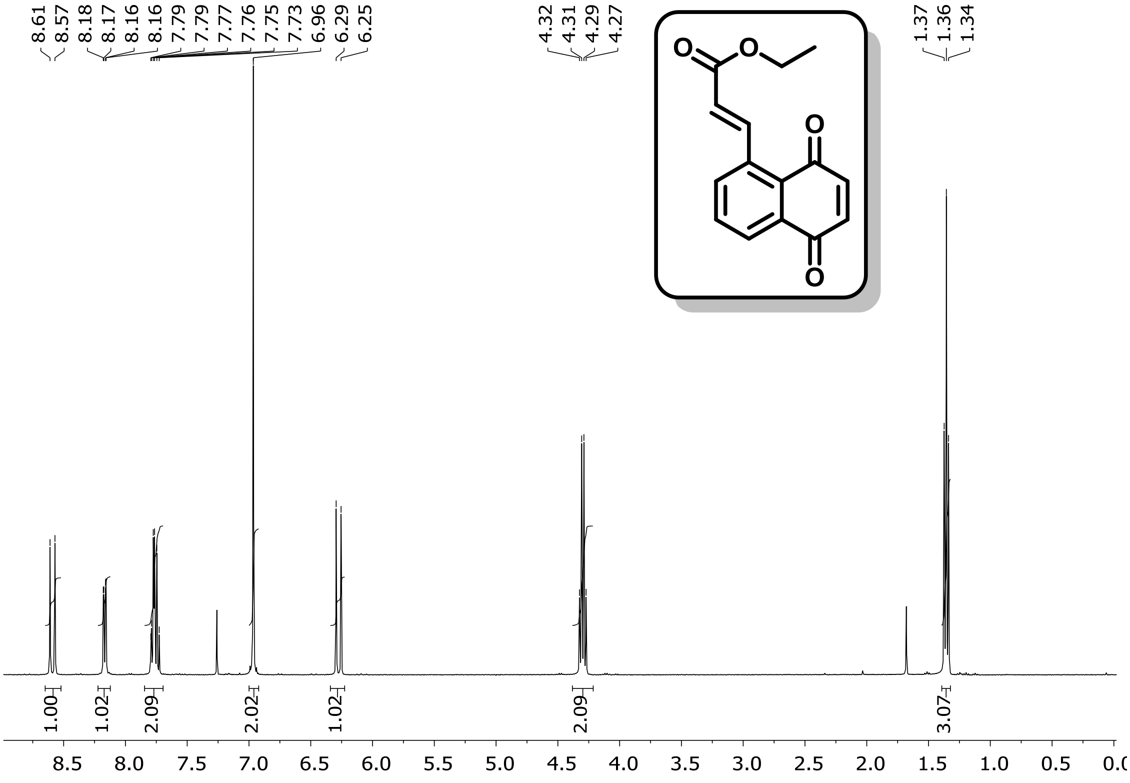
8.61
8.57
8.18
8.17
8.16
8.16
7.79
7.79
7.77
7.76
7.75
7.73
6.96
6.29
6.25
4.32
4.31
4.29
4.27
1.37
1.36
1.34
1.00
1.02
2.09
2.02
1.02
2.09
3.07
8.5 8.0 7.5 7.0 6.5 6.0 5.5 5.0 4.5 4.0 3.5 3.0 2.5 2.0 1.5 1.0 0.5
O
O
O
O

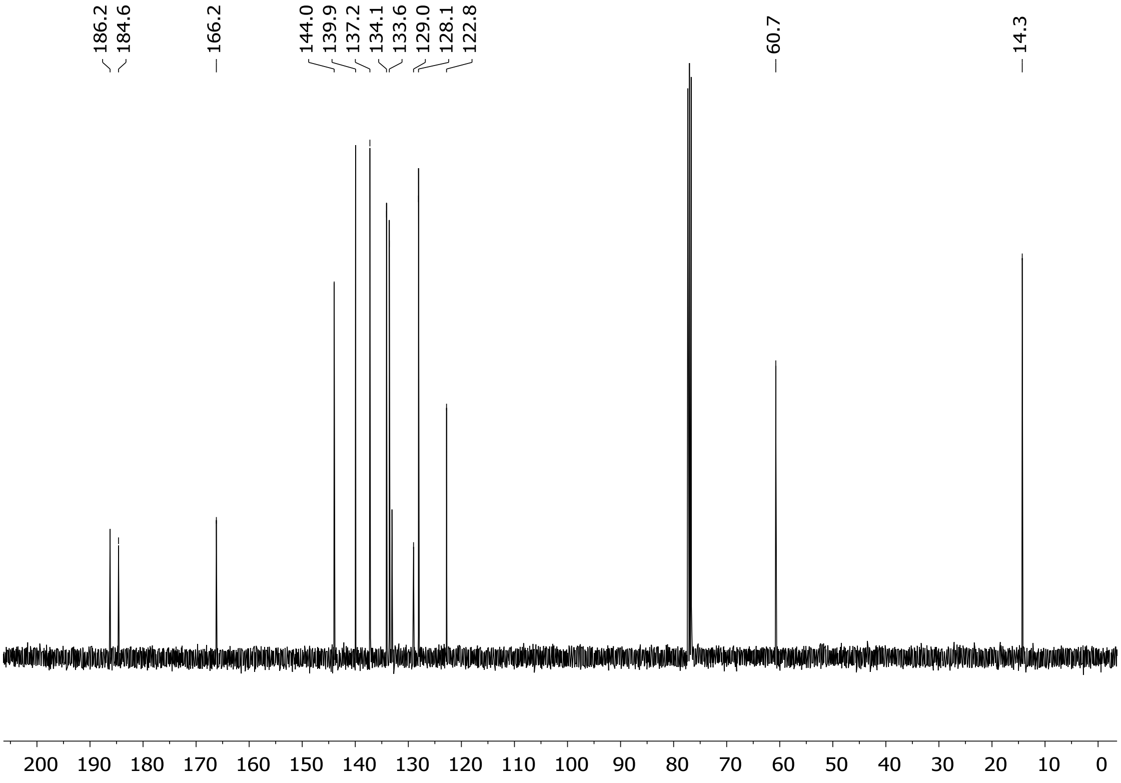
186.2
184.6
166.2
144.0
139.9
137.2
134.1
133.6
129.0
128.1
122.8
60.7
14.3
200 190 180 170 160 150 140 130 120 110 100 90 80 70 60 50 40 30 20 10 0

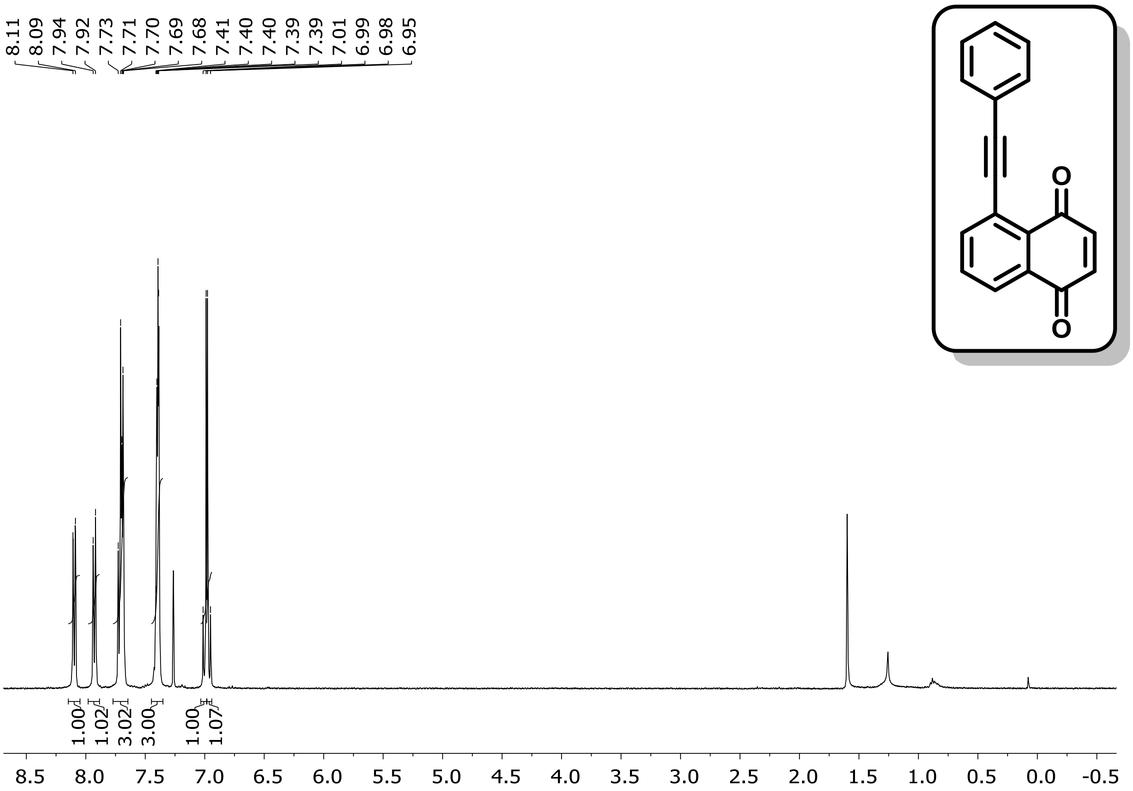
8.11
8.09
7.94
7.92
7.73
7.71
7.70
7.69
7.68
7.41
7.40
7.40
7.39
7.39
7.01
6.99
6.98
6.95
1.00
1.02
3.02
3.00
1.00
1.07
8.5 8.0 7.5 7.0 6.5 6.0 5.5 5.0 4.5 4.0 3.5 3.0 2.5 2.0 1.5 1.0 0.5 0.0 -0.5
O
O

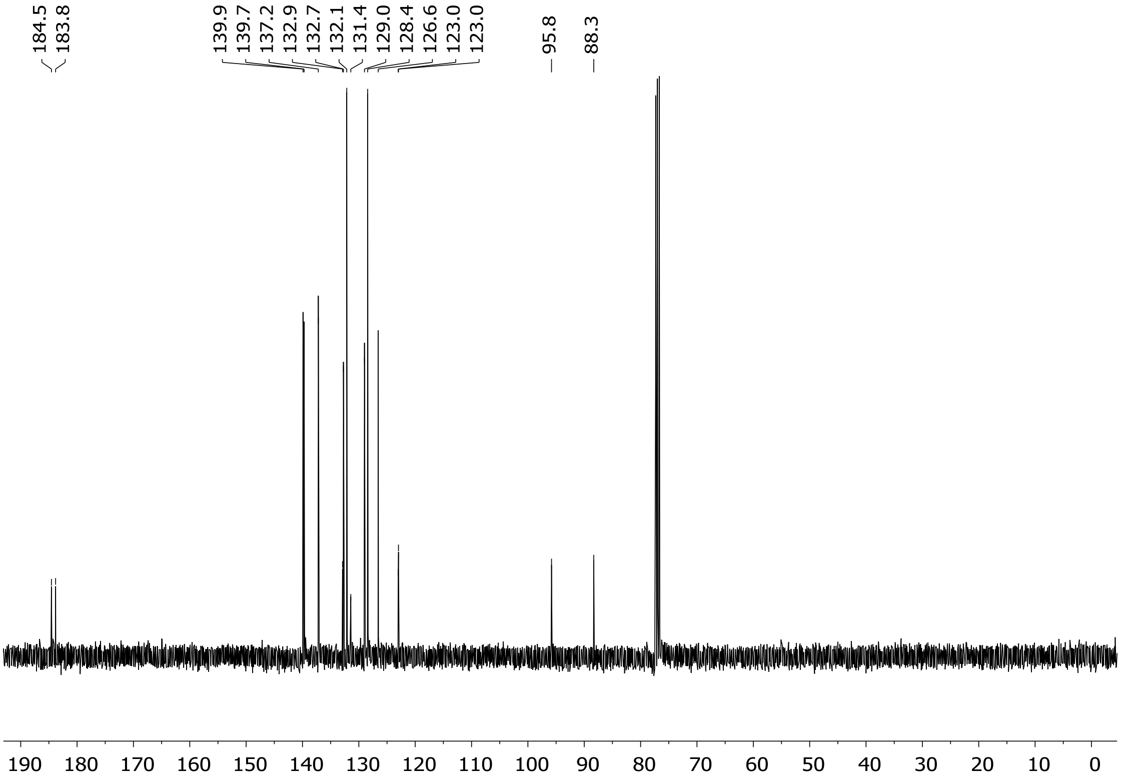
184.5
183.8
139.9
139.7
137.2
132.9
132.7
132.1
131.4
129.0
128.4
126.6
123.0
123.0
95.8
88.3
190 180 170 160 150 140 130 120 110 100 90 80 70 60 50 40 30 20 10 0

References

1. Hastings, C.J.; Pluth, M.D.; Bergman, R.G.; Raymond, K.N.; *J. Am. Chem. Soc.*, **2010**, *132*, 6938.

2. Schumann, H.; Keitsch, M.R.; Winterfeld, J.; Muhle, S.; Molander, G.A.; *J. Organomet. Chem.* **1998**, *559*, 181.

3. Gassman, P.G.; Mickelson, J.W.; Sowa Jr., J.R.; *J. Am. Chem. Soc.*, **1992**, *114*, 6943.

4. Romanov-Michailidis, F.; Sedillo, K.F.; Neely, J.M.; Rovis, T.; *J. Am. Chem. Soc.*, **2015**, *137*, 8892.

5. Boyer, P.M.; Roy, C.P.; Bielski, J.M.; Merola, J.S.; *Inorg. Chim. Acta*, **1996**, *245*, 7.

6. Tietze, L.F.; Güntner, C.; Gericke, K.M.; Schuberth, I.; Bunkoczi, G.; *Eur. J. Org. Chem.* **2005**, 2459.

7. Bendiabdellah, Y.; Rahman, K.M.; Uranchimeg, B.; Nahar, K.S.; Antonow, D.; Shoemaker, R.H.; Melillo, G.; Zinzalla, G.; Thurston, D.E.; *Med. Chem. Commun.*, **2014**, *5*, 923.

8. Bergman, J.; Romero, I.; *Arkivoc*, **2009**, *6*, 191.

9. Cai, J.; Li, Y.; Chen, J.; Wang, P.; Ji, M.; *Res. Chem. Intermed.*, **2015**, *41*, 1.

10. Newman, M.S.; Choudhary, A.R.; *Org. Prep. Proced. Int*, **1989**, *21*, 359.

11. Koike, T.; Tanabe, M.; Takeuchi, N.; Tobinaga, S.; *Chem. Pharm. Bull.*, **1997**, *45*¸ 243.

12. Ivashkina, N.V.; Romanov, V.S.; moroz, A.A.; Shvartsberg, M.S.; *Russ. Chem. B.;* **1984**, *33*, 2345.

13. Ren, J.; Lu, L.; Xu, J.; Yu, T.; Zeng, B.; *Synthesis*, **2015**, *47*, 2270.

14. Crecely, R.W.; Crecely, K.M.; Goldstein, J.H.; *J. Mol. Spectrosc.*, **1969**, *32*, 407.
